# Chemo‐ and Site‐Selective Electro‐Oxidative Alkane Fluorination by C(sp^3^)−H Cleavage

**DOI:** 10.1002/chem.202201654

**Published:** 2022-08-31

**Authors:** Maximilian Stangier, Alexej Scheremetjew, Lutz Ackermann

**Affiliations:** ^1^ Institut für Organische und Biomolekulare Chemie Wöhler Research Institute for Sustainable Chemistry (WISCh) Georg-August-Universität Göttingen Tammannstrasse 2 37077 Göttingen Germany

**Keywords:** C(sp^3^)−H functionalization, electrosynthesis, fluoride source, fluorination

## Abstract

Electrochemical fluorinations of C(sp^3^)−H bonds with a nucleophilic fluoride source have been accomplished in a chemo‐ and site‐selective fashion, avoiding the use of electrophilic F^+^ sources and stoichiometric oxidants. The introduced metal‐free strategy exhibits high functional group tolerance, setting the stage for late‐stage fluorinations of biorelevant motifs. The synthetic utility of the C(sp^3^)−H fluorination was reflected by subsequent one‐pot arylation of the generated benzylic fluorides.

## Graphical abstract

**Metal‐free electrochemical** C(sp^3^)−H fluorination was accomplished without the requirement for directing groups and electrophilic fluorine sources, featuring high levels of site‐ and chemoselectivity.

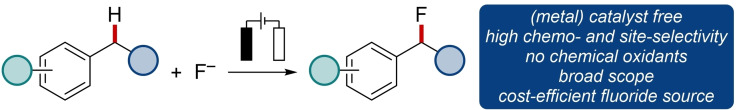

## Introduction

**Scheme 1:**
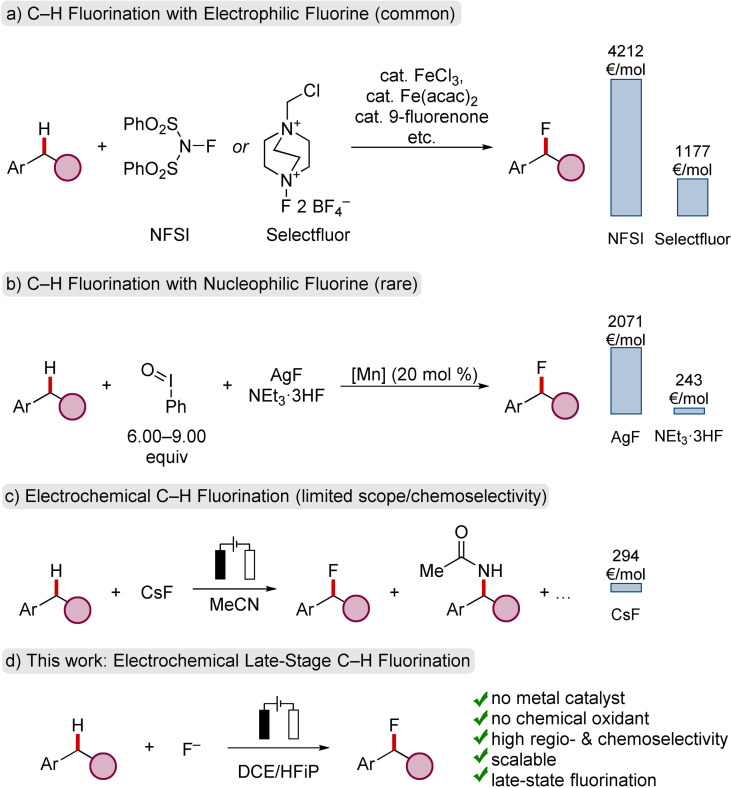
C−H Fluorination with a) electrophilic fluorine reagents, b) nucleophilic fluorides, c) under electrochemical conditions. d) electrochemical late‐stage C−H fluorination.^[13]^

The unique properties of the C−F bond^[1]^ substantially impact the development of medically relevant drugs,^[2]^ crop protection agents^[3]^ and material sciences.^[4]^ Due to the ubiquitous presence of benzylic C(sp^3^)−H bonds in feedstock and fine chemicals, methods for their direct functionalization are in high demand.^[5]^ While the classical construction of C(sp^3^)−F bonds relies on desoxyfluorination of alcohols, direct C−H fluorinations have been developed using electrophilic or radical fluorination agents.^[6]^ With respect to the benzylic fluorination with electrophilic fluorine sources, either organic, inorganic or radical initiators have been utilized (Scheme 1a).^[7]^ For instance, potassium persulfate^[7a]^ or organic (photo)catalysts, such as tetracyanobenzene^[7b]^, *N*,*N*‐dihydroxy‐pyromellitimide^[7c]^ or fluorenone,^[7d]^ among others,^[8]^ were described to facilitate the C(sp^3^)−H fluorination. In terms of metal catalysts, copper,^[9]^ iron^[10]^ and decatungstate^[11]^ were reported to accomplish benzylic fluorination with electrophilic fluorine reagents.

In sharp contrast, the use of hydrogen fluoride sources (e. g. NEt_3_ ⋅ 3HF) or metal fluorides, appear to be significantly more desirable and resource‐economic. However, the direct C−H fluorination with nucleophilic fluorides is considerably more challenging, due to the low nucleophilicity, competitive hydrogen bonding interactions and the low solubility of metal fluorides. Major progress has been achieved by Groves and Doyle as direct oxidative C−H fluorinations were accomplished using (metal) fluorides as fluoride source (Scheme 1b).^[12]^ Despite of these indisputable advances, these methods suffer from the use of expensive metal catalysts and require chemical oxidants with up to six equivalents of cost‐intensive hypervalent iodine(III) reagents.

In recent years, organic electrochemistry has emerged as a transformative platform, providing resource‐economic access to valuable scaffolds.^[14]^ In terms of electrochemically‐enabled benzylic C−H functionalizations, oxygenations,^[15]^ aminations,^[16]^ cyanations,^[17]^ and iodinations^[18]^ have been reported. Although electrochemical C−H fluorinations of benzylic substrates are of major interest using abundant fluoride sources, established protocols are thus far limited in scope, and are characterized by low levels of chemoselectivity, as competitive acetamidations from the acetonitrile solvent cannot be avoided (Scheme 1c).^[19]^ Thus, we wondered whether a chemical oxidant‐ and metal‐free electrochemical fluorination (ECF) manifold could be viable, combining high chemoselectivities, thus far typically observed in fluorination reactions with electrophilic fluorine sources (Scheme 1d).

## Results and Discussion

We initiated our studies by probing the electrochemical fluorination of ibuprofen methyl ester **1 a** using readily available NEt_3_ ⋅ 3HF as the source of nucleophilic fluoride, in acetonitrile with platinum electrodes (Table 1). Under inert atmosphere, the selective functionalization occurred at the methylene group. Unfortunately, competing acetamidation by nucleophilic attack of acetonitrile formed **3 a** in equimolar quantities (entry 1).[^19c^, ^20^] To overcome this limitation, optimization studies with respect to the solvent system were performed. Specifically, reported reaction conditions,^[21]^ with a mixture of acetonitrile and HFIP, and CsF were tested. However, here, only traces of the desired product **2 a** were obtained. In contrast, oxygenation by HFIP was observed as the main competitive reaction (entry 2).

**Table 1:** Optimization for the site‐selective C−H fluorination.

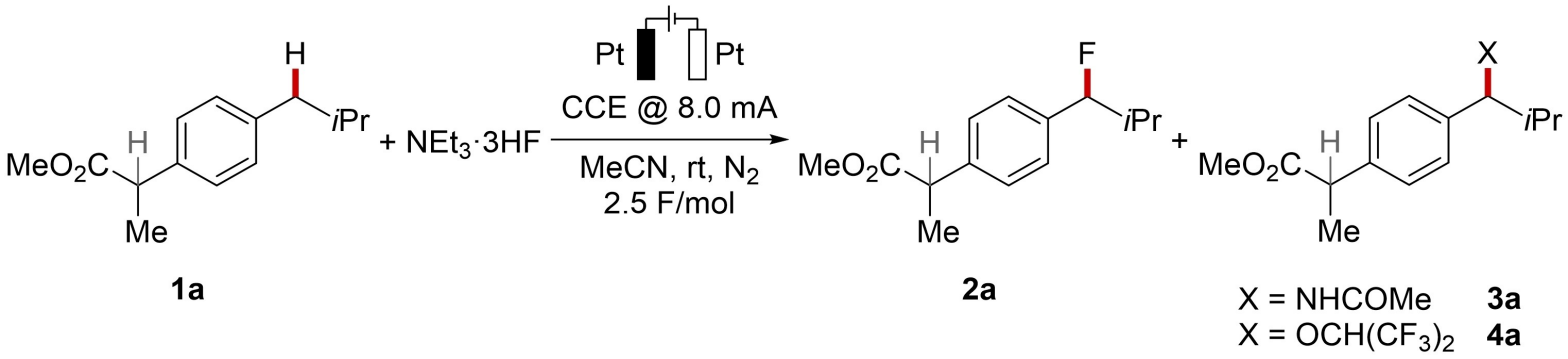				
Entry	Deviation from standard conditions	Yield [%]		
		**2 a**	**3 a**	**4 a**
1	–	19	19	–
2^[a]^	MeCN : HFIP (4 : 1)	<5	–	49
3	MeCN : HFIP (4 : 1)	28	14	6
4	MeCN : HFIP (4 : 1), RVC anode	10	6	7
5	DCE : HFIP (2 : 1), RVC anode	82	–	–
6^[b]^	DCE : HFIP (2 : 1), RVC anode	55	–	–
7^[c]^	DCE : HFIP (2 : 1), RVC anode	66	–	–
8	DCE : HFIP (2 : 1), GC anode	72	–	–
9	DCE : HFIP (2 : 1)	85	–	–
**10**	**DCE : HFIP (2 : 1), GF anode**	**92**	–	–
11	CH_2_Cl_2_ : HFIP (2 : 1), GF anode	90	–	–
12	DCE : TFE (2 : 1), GF anode	37	–	–^[d]^

Undivided cell, platinum anode and platinum cathode, **1 a** (0.50 mmol), NEt_3_ ⋅ 3HF (1.0 mL, 12 equiv), solvents (3.0 mL) under inert atmosphere. ^1^H NMR yields with CH_2_Br_2_ as internal standard are given. [a] With CsF (0.3 M) and solvents (4.0 mL). [b] With NEt_3_ ⋅ 3HF (0.5 mL, 6.1 equiv). [c] With NEt_3_ ⋅ 3HF (0.25 mL, 3.1 equiv) and *n*Bu_4_NBF_4_ (0.1 M). [d] Analogous trifluoroethoxy‐substituted product was formed in 23 % yield.

**Scheme 2:**
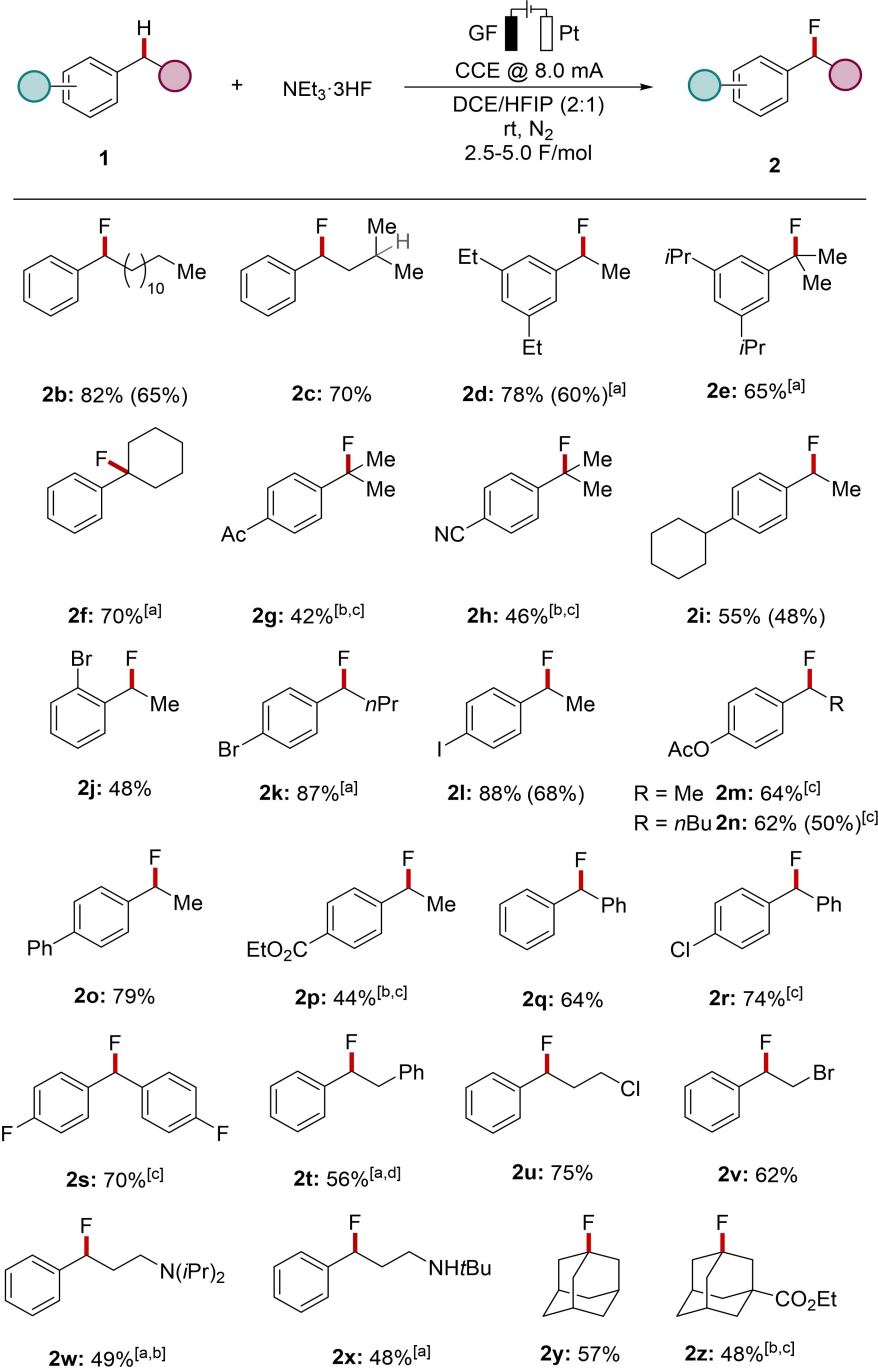
Scope for the site‐selective electrochemical C−H fluorination. ^1^H‐ or ^19^F NMR yields with CH_2_Br_2_ or PhCF_3_ as internal standard are provided. Isolated yields in parentheses. [a] 0 °C. [b] 10 mA CCE. [c] −20 °C. [d] 16 mA.

When the fluoride source was changed to NEt_3_ ⋅ 3HF, mainly product **2 a** was formed, although in an unsatisfactory yield (entry 3). Interestingly, changing the anode material to reticulated vitreous carbon (RVC) led to an overall decrease in conversion and selectivity (entry 4). In contrast, the use of a DCE/HFIP solvent mixture dramatically increased conversion to the desired product and suppressed the undesired competitive oxygenation (entry 5). Notably, employing decreased amounts of the fluoride source slightly decreased the conversion to product **2 a** (entry 6). Nevertheless, a further decrease to only 3.00 equivalents of NEt_3_ ⋅ 3HF could be compensated by the addition of a supporting electrolyte (entry 7). Next, the role of the anode material was further examined. While platinum, glassy carbon (GC) and RVC could be used for the transformation (entries 5,8,9), graphite felt (GF) proved superior (entry 10). Alternative solvent mixtures were tested, and DCE could effectively be substituted by CH_2_Cl_2_ (entry 11). In contrast, the use of TFE furnished larger amounts of an undesired oxygenated product, likely due to its increased nucleophilicity (entry 12).^[22]^ With the optimal conditions (entry 10) for the newly developed fluorination of C(sp^3^)−H bonds in hand, we probed its scope (Scheme 2). First, various linear and branched alkylbenzenes **1 b**–**1 f** were probed under the electro‐oxidative reaction conditions. Substrates with tertiary benzylic carbons proved to be suitable, even in the presence of strongly electron‐withdrawing groups (**1 g**–**1 h**). Substrate **1 i**, featuring both, a tertiary, and a secondary benzylic position, was site‐selectively fluorinated at the arguably more kinetically acidic methylene C−H bond.^[23]^ Haloarenes **1 j**–**1 l** were fully tolerated in the electro‐oxidation, and furnished the corresponding products in good to excellent yields. Scaffolds bearing moderately electron‐donating and withdrawing substituents (**1 m**–**1 p**), such as phenyl, acetoxy, and ester groups, proved to be amenable substrates for the electrochemical fluorination strategy.

Similarly, unsubstituted and moderately electron‐deficient diarylmethanes effectively underwent the transformation (**1 q**–**1 s**). Next, different groups at the aliphatic sidechain were tested towards their capability to undergo electrochemical fluorination. Aryl‐ or halogen‐substituted derivatives furnished the corresponding products **2 t**–**2 v** with excellent chemo‐selectivity. Remarkably, tertiary, and secondary amines **1 w**–**1 x** were compatible within the developed electro‐oxidative fluorination. Furthermore, we were glad to observe that adamantanes^[24]^ **1 y** and **1 z** were likewise fluorinated at the tertiary positions under the investigated conditions. Notably, substrates **1 g**, **1 h**, **1 m**, **1 n**, **1 p**, **1 r**, **1 s**, and **1 z** bearing electron‐withdrawing substituents generally reacted more efficiently at lower temperatures. Unfortunately, multiple electron‐withdrawing substituents or strongly electron‐donating alkoxy and amino substituents on the arene failed to furnish the desired products in satisfactory yields under otherwise identical reaction conditions (Supporting Information, Figure 73).

**Scheme 3:**
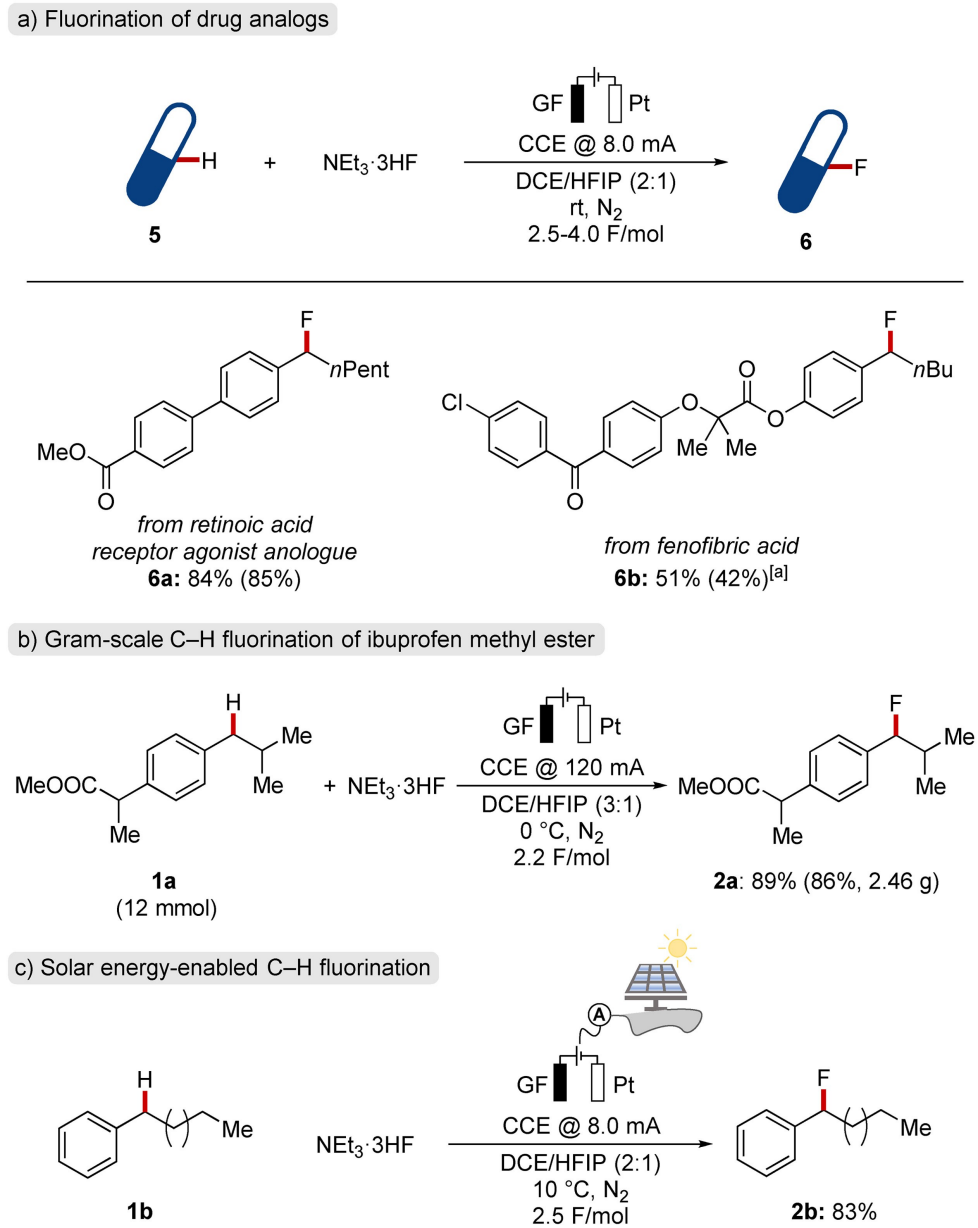
Practical aspects of the ECF. ^1^H NMR yields with CH_2_Br_2_ as internal standard are provided. Isolated yields in parentheses. [a] −20 °C, 20 mA.

Additionally, the synthetic utility of the electrochemical C(sp^3^)−H fluorination was explored towards the late‐stage diversification of biologically active compounds **5** (Scheme 3a). Besides ibuprofen ester **1 a**, retinoic acid receptor agonist analogue **5 a** and fenofibric acid derivative **5 b**,^[25]^ were selectively converted to the desired products **6 a** and **6 b**, respectively. The robustness was further reflected by a large‐scale fluorination of ibuprofen ester **1 a** to obtain 2.46 g (86 %) of the desired product **2 a** (Scheme 3b). High resource economy and reliability was demonstrated by using constant current solely derived from solar energy using a commercially available solar panel, to furnish product **2 b** in comparable yield (Scheme 3c).

**Scheme 4:**
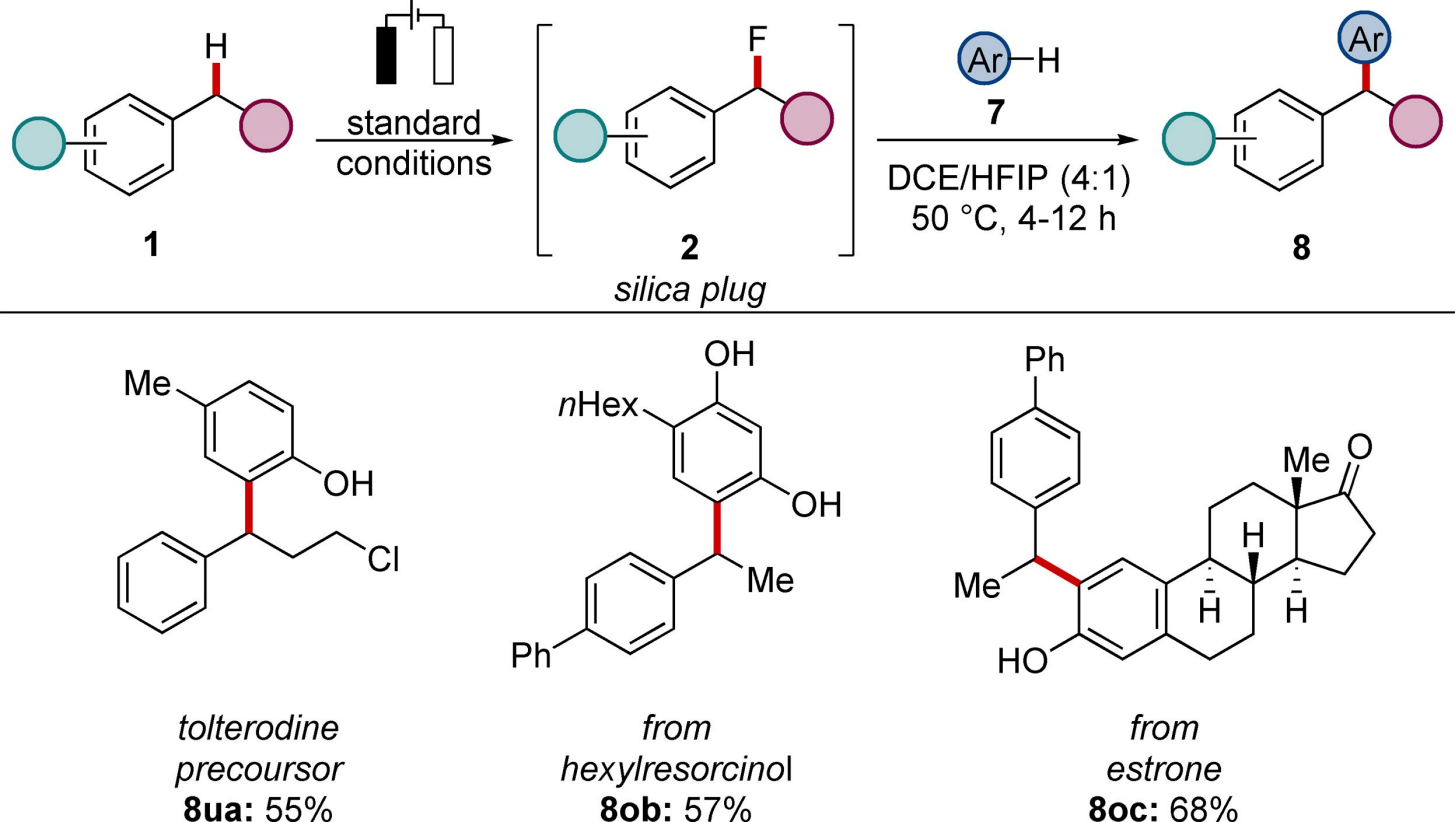
Facile C(sp^3^)−H arylation with arenes **7**.

In addition to the unique properties of fluorinated molecules, fluorides have also been utilized as strategic intermediates for the subsequent metal‐free benzylation of electron‐rich arenes.^[ 9a, 26a–b]^ We therefore explored whether the newly developed strategy allowed the facile displacement reaction. Indeed, after simple filtration of the reaction mixture over silica, tolterodine precursor **8 ua** was obtained in good efficiency after 2 steps (Scheme 4). Likewise, the strategy was employed to the functionalization of bioactive resorcinol and estrone with biphenyl **1 o** to furnish the desired products **8 ob** and **8 oc** in high yields.

**Scheme 5:**
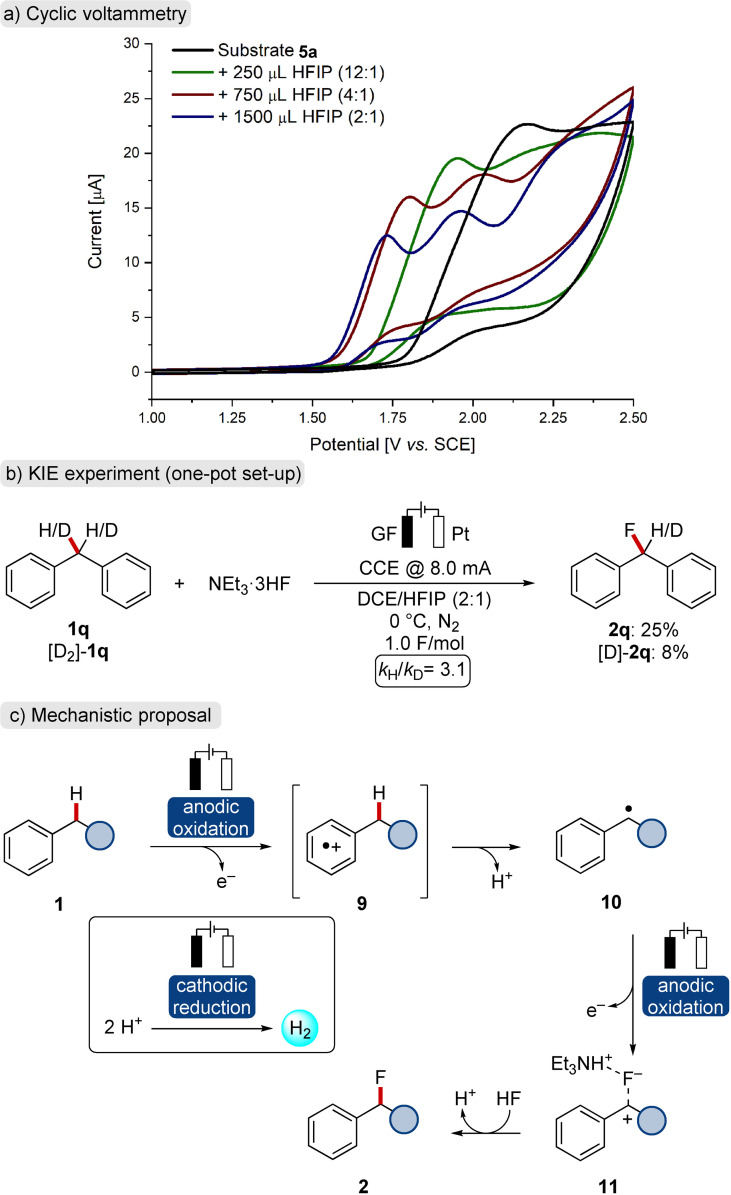
Mechanistic insights and proposed scenario for the ECF.

Mechanistic studies were conducted to rationalize the observed levels of orthogonal selectivity of the direct fluorination strategy. Cyclic voltammetry was performed and substrate **5 a** exhibited an irreversible response at *E*_p_=2.16 V vs. SCE in dichloroethane (Scheme 5a, black line). Upon addition of small amounts of HFIP, the oxidation event slightly shifted towards less positive potentials (green line, *E*_p_=1.95 V vs. SCE). This effect was boosted at a DCE/HFIP ratio of 4 : 1 (red line, *E*_1,p_=1.80 V vs. SCE). In addition, a second oxidation event at *E*_2,p_=2.03 V vs. SCE became apparent. This result suggests that HFIP enables the generation of a stable radical intermediate by a proton‐coupled electron transfer,^[27]^ which can undergo a second discrete oxidation. The oxidation potentials of **5 a** were additionally lowered towards *E*_1,p_=1.73 V vs. SCE and *E*_2,p_=1.96 V vs. SCE, when the optimized solvent ratio of DCE/HFIP=2 : 1 was used (blue line). A beneficial effect of NEt_3_ ⋅ 3HF on the second oxidation event was further revealed, suggesting the stabilization of a generated benzyl cation by the fluoride source (Supporting Information, Figure 3). Reactions with deuterated substrate [D_2_]‐**1 q** showed a significant kinetic isotope effect (KIE) without H/D exchange, being indicative of an irreversible and rate‐limiting C−H cleavage (Scheme 5b, Supporting Information, Figure 11).

Based on the cyclic voltammetry experiments and literature precedence,^[ 15a,16a–b, 17]^ a mechanistic scenario of the electro‐oxidative C−H fluorination is proposed in Scheme 5c. Initially, the arene is oxidized to a radical cation **9**, which is stabilized by the HFIP co‐solvent.^[28]^ Thereafter, rate‐limiting heterolytic C−H cleavage furnishes radical **10**.^[29]^ In a second anodic oxidation, the corresponding benzyl cation **11** is formed, which is trapped by a fluoride ion to deliver the desired product **2**. At the platinum cathode, the hydrogen evolution reaction (HER) takes place by means of electrochemical proton reduction.

## Conclusion

In conclusion, we have developed a selective electrochemical C(sp^3^)−H fluorination, avoiding expensive electrophilic fluorine reagents by readily available NEt_3_ ⋅ 3HF. Stoichiometric oxidants and precious metal catalysts, as well as directing groups, are not required. The strategy showed broad functional group tolerance, allowing the late‐stage functionalization of bioactive drugs. The practical utility was further substantiated by a large scale and sunlight‐enabled electrochemical fluorination. Ultimately, C(sp^3^)−H fluorination set the stage for a facile arylation with arenes, and mechanistic insights shed light on the crucial role of HFIP in facilitating a proton‐coupled electron transfer.

## Experimental Section

Representative procedure for the synthesis of product **2 i**: 1‐Cyclohexyl‐4‐ethylbenzene **1 i** (94.3 mg, 0.50 mmol), DCE (2.0 mL), HFIP (1.0 mL), and NEt_3_ ⋅ 3HF (1.0 mL) were placed in a 10 mL undivided cell (pre‐dried Schlenk tube) under inert atmosphere. A graphite felt (GF) anode (25 mm×10 mm×6.0 mm) and a platinum cathode (25 mm×10 mm×0.125 mm) were attached to an electrode holder which was assembled on the electrolysis cell. Electrosynthesis was performed at rt with a constant current of 8.0 mA until 2.5 F/mol were passed (4.2 h). After electrolysis, the reaction mixture was filtered over a plug of silica. The platinum cathode and the graphite felt anode were washed with EtOAc (Pt: 1×5.0 mL; C: 3×10 mL) and the resulting fraction was filtered over the same silica plug. After rinsing the silica plug with an additional mixture of *n*hexane/EtOAc 3:1 (75 mL), the solvents were removed in vacuo. After conducting the quantitative NMR analysis of the crude mixture with CH_2_Br_2_ and PhCF_3_ as internal standards (yield: 55 % by ^1^H NMR, 60 % by ^19^F{^1^H} NMR), the solvents were removed in vacuo, and the residue was purified by column chromatography on silica (partially neutralized by the addition of 4 wt‐% NEt_3_ in a pentane suspension and dried prior to loading; eluent: *n*hexane) to obtain the product **2 i** as a colorless oil (49.6 mg, 0.24 mmol, 48 %).

## Conflict of interest

The authors declare no conflict of interest.

1

## Supporting information

As a service to our authors and readers, this journal provides supporting information supplied by the authors. Such materials are peer reviewed and may be re‐organized for online delivery, but are not copy‐edited or typeset. Technical support issues arising from supporting information (other than missing files) should be addressed to the authors.

Supporting Information

## Data Availability

The data that support the findings of this study are available in the supplementary material of this article.
