# Supplementary material for: Chemo‐ and Site‐Selective Electro‐Oxidative Alkane Fluorination by C(sp3)−H Cleavage
Source: Chemistry. 2022 Aug 31;28(60):e202201654. doi: 10.1002/chem.202201654 (PMC9804291; doi:10.1002/chem.202201654)
Supplement: Supplementary file 1 — Supporting Information [file CHEM-28-0-s001.pdf]

Chemistry–A European Journal

Supporting Information

Chemo- and Site-Selective Electro-Oxidative Alkane Fluorination by C(sp^3)–H Cleavage

Maximilian Stangier, Alexej Scheremetjew, and Lutz Ackermann*

Table of Contents

1. General Information 3
2. Variations of the Standard Conditions 5
3. General Procedures 6
4. Cyclic Voltammetry 8
5. Solar Energy-Enabled Fluorination 11
6. Comparison with Previously Reported Conditions 12
7. H/D exchange experiment 15
8. Kinetic Isotope Effect Studies 17
9. Gram-Scale Reaction 20
10. Quantitative ^{1}H and $^{19}F\{H\}$-NMR Spectra for Benzyl Fluorides 23
11. Late-Stage Fluorination 57
12. $C(sp^3)$–H Arylation 60
13. Unsuccessful Substrates 63
14. NMR Spectra 64
15. References 93

1. General Information

All reactions were carried out in undivided electrochemical cells (10 mL) using pre-dried glassware, if not noted otherwise. **1l**-d_2 was synthesized according to a previously described procedure.[1] Platinum electrodes (10 mm × 15 mm × 0.25 mm, 99.9%; obtained from ChemPur®, Karlsruhe, Germany) and graphite felt electrodes (10 mm × 15 mm × 6 mm, SIGRACELL® GFA 6 EA, obtained from SGL Carbon, Wiesbaden, Germany) were connected using stainless steel electrode holders. Electrocatalysis was conducted using a Metrohm Multiautolab system M204 set-up in constant current mode. Cyclic Voltammetry studies were performed using a Metrohm Autolab PGSTAT204 workstation and Nova 2.1 software. Yields refer to determination by ^{1}H- and ^{19}F-NMR spectroscopy with internal standards. Isolated compounds are estimated to be >95% pure as determined by ^{1}H-NMR. Column chromatography was carried out on Merck silica gel 60 (40–63 µm). For chromatographic purifications carried out on partially neutralized silica, the silica gel was suspended in pentane followed by the addition of NEt_3 (4 wt-% of the silica) and removal of the solvent was in a rotary evaporator prior to loading. Benzylic fluorides can be sensitive to slightly acidic conditions and might decompose during purification on silica gel or upon concentration in glassware.[2] NMR spectra were recorded on a Bruker Avance III 300, Avance III 400 or Avance Neo 600 in the solvent indicated; chemical shifts (δ) are given in ppm relative to the resonances of $SiMe_4$ (^{1}H- and ^{13}C-NMR) and $CFCl_3$ (^{19}F{^{1}H}-NMR), and were referenced, to the residual solvent peak. Quantitative ^{1}H- and ^{19}F{^{1}H}-NMR spectra were recorded with relaxation delays of 0.1 s and 0.5 s, respectively. All IR spectra were recorded on a Bruker FT-IR Alpha-P device. EI-MS was recorded on Jeol AccuTOF at 70eV, ESI-MS on Bruker MicrOTOF and maXis. GC-MS was recorded on Agilent 7890B and Agilent 5977B. Melting points were measured on a Stuart SMP3 melting point apparatus from Barloworld Scientific. Values are uncorrected.

2. Variations of the Standard Conditions

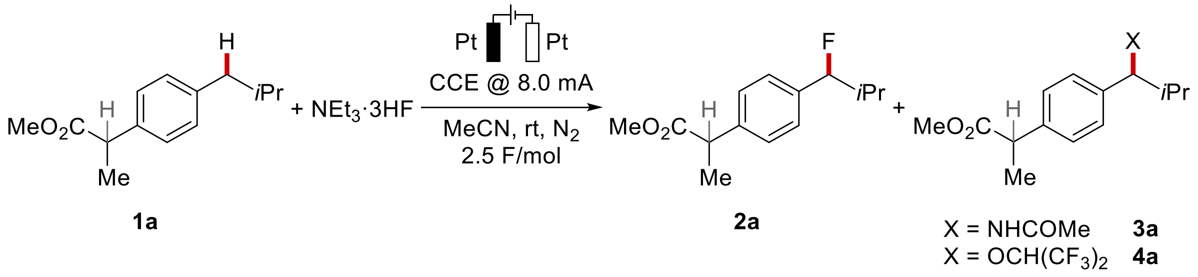

Entry	Fluoride Source	Anode	Solvent	Yield [%]		
				**2a**	**3a**	**4a**
1	$NEt_3 \cdot 3HF$	Pt	MeCN	19	19	-
2	CsF[a]	Pt	MeCN:HFIP (4:1)	<5	-	49
3	$NEt_3 \cdot 3HF$	Pt	MeCN:HFIP (4:1)	28	14	6
4	$NEt_3 \cdot 3HF$	RVC	MeCN:HFIP (4:1)	10	6	7
5	$NEt_3 \cdot 3HF$	RVC	DCE:HFIP (2:1)	82	--	--
6	$NEt_3 \cdot 3HF$[b]	RVC	DCE:HFIP (2:1)	55	--	--
7	$NEt_3 \cdot 3HF$[c]	RVC	DCE:HFIP (2:1)	66	--	--
8	$NEt_3 \cdot 3HF$	GC	DCE:HFIP (2:1)	72	--	--
9	$NEt_3 \cdot 3HF$	Pt	DCE:HFIP (2:1)	85	--	--
**10**	**$NEt_3 \cdot 3HF$**	**GF**	**DCE:HFIP (2:1)**	**92**	**--**	**--**
11	$NEt_3 \cdot 3HF$	GF	DCM:HFIP (2:1)	90	--	--
12	$NEt_3 \cdot 3HF$	GF	DCE:TFE (2:1)	37	--	--[d]

Undivided cell, platinum cathode, **1a** (0.50 mmol), $NEt_3 \cdot 3HF$ (1.0 mL, 12 equiv), solvents (3.0 mL) under inert atmosphere. NMR yields with CH_2Br_2 as internal standard are given. [a] CsF (0.3 M) and solvents (4.0 mL). [b] With $NEt_3 \cdot 3HF$ (0.5 mL, 6.1 equiv). [c] With $NEt_3 \cdot 3HF$ (0.25 mL, 3.1 equiv) and *n*Bu_4NBF_4 (0.1 M). [d] Analogous trifluoroethoxy-substituted product was formed in 23% yield.

3. General Procedures

Warning: Fluoride reagents should be handled only by trained staff and under appropriate safety measurements (lab coat, gloves, and eye protection) inside a fume hood, as they are toxic reagents.

General Procedure A-1 for the electrochemical fluorination of benzylic C–H bonds

Benzylic substrate **1** (0.50 mmol), DCE (2.0 mL), HFIP (1.0 mL), and $NEt_3 \cdot 3HF$ (1.0 mL) were placed in a 10 mL undivided cell under inert atmosphere. A graphite felt (GF) anode (25mm × 10 mm × 6.0 mm) and a platinum cathode (25 mm × 10 mm × 0.125 mm) were attached to an electrode holder which was assembled on the electrolysis cell. Electrosynthesis was performed, unless noted otherwise, at rt with a constant current of 8.0 mA until 2.5 F/mol were passed (4.2 h). After electrolysis, the reaction mixture was filtered over a plug of silica. The platinum cathode and the graphite felt anode were washed with EtOAc (Pt: 1 × 5.0 mL; C: 3 × 10 mL) and the resulting fraction was filtered over the same silica plug. After rinsing the silica plug with an additional mixture of *n*hexane/ethylacetate (75 mL), the solvents were removed in vacuo. CH_2Br_2 (36 µL, 0.50 mmol) and $PhCF_3$ (62 µL, 0.50 mmol) were added to the resulting residue and the mixture was submitted for NMR analyses.

General Procedure A-2 for the electrochemical fluorination of benzylic C–H bonds

The electrolysis was set up following general procedure A-1. After electrolysis, the platinum cathode and the graphite felt anode were washed with DCM (Pt: 1 × 5.0 mL; C: 3 × 5.0 mL). The solvents were combined with the reaction mixture and diluted with *n*hexane (50 mL). The organic layers were washed three times with water (3 × 20 mL) in a separatory funnel and dried over Na_2SO_4. After adding silica gel (2.0 g), the mixture was filtered over a pad of celite, and the solvents were removed in vacuo. CH_2Br_2 (36 µL, 0.50 mmol) and $PhCF_3$ (62 µL, 0.50 mmol) were added to the residue and the mixture was submitted for NMR analyses.

General Procedure B for the benzylation of electron-rich arenes

Following general procedure A-1 for the electrochemical fluorination, arene **5** (2.50 mmol) was added to the filtrate of the silica plug (pentane/DCM = 9:1, 75 mL) and the solvents were removed in vacuo. Subsequently the residue was dissolved in DCE (2.0 mL) and stirred at room temperature. Then HFIP (0.5 mL) was added dropwise, and the reaction was heated to 50 °C.

When the benzylic fluoride was consumed, as indicated by TLC or GC-MS, the reaction was stopped, the solvents removed, and the residue purified by column chromatography.

4. Cyclic Voltammetry

Cyclic voltammetry measurements were conducted with a Metrohm Autolab PGSTAT204 potentiostat and Nova 2.1 software. For all experiments, a glassy carbon working electrode (disk, diameter: 3 mm), a platinum wire counter electrode, and either a SCE reference electrode or an Ag/Ag^{+} quasi-reference electrode with ferrocene as an internal standard were used. DCE and HFIP with 0.1 mol/L $n\mathrm{Bu_4NPF_6}$ as conducting salt served as electrolyte for the measurements. The voltammograms were recorded under inert atmosphere at a scan rate of 100 mV/s, if not indicated otherwise.

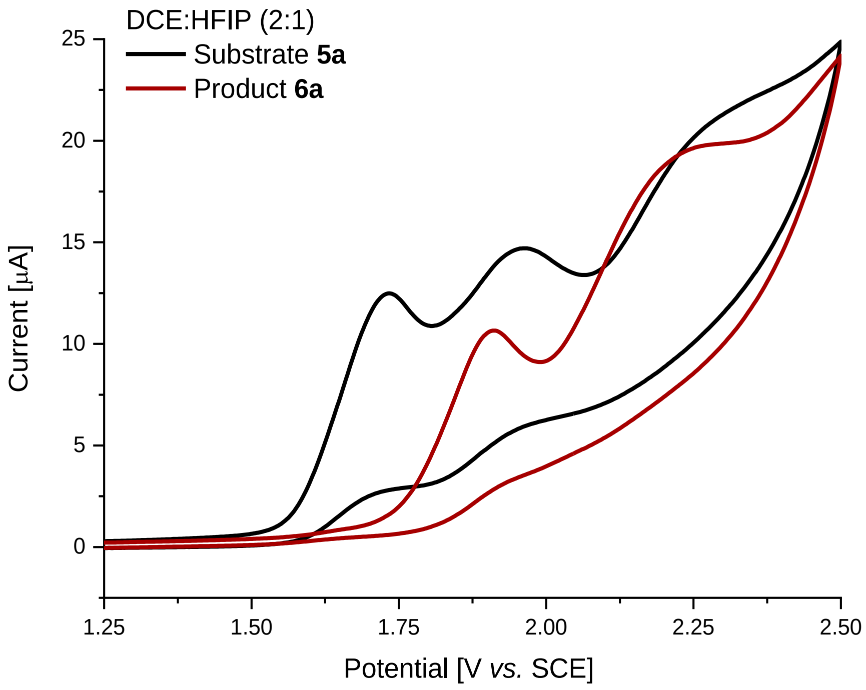

Figure 1 Cyclic voltammetry in DCE:HFIP (2:1) with $n\mathrm{Bu_4NPF_6}$ (0.1 M) at 100 mV/s and glassy carbon working electrode. Substrate **5a** (black); product **6a** (red).

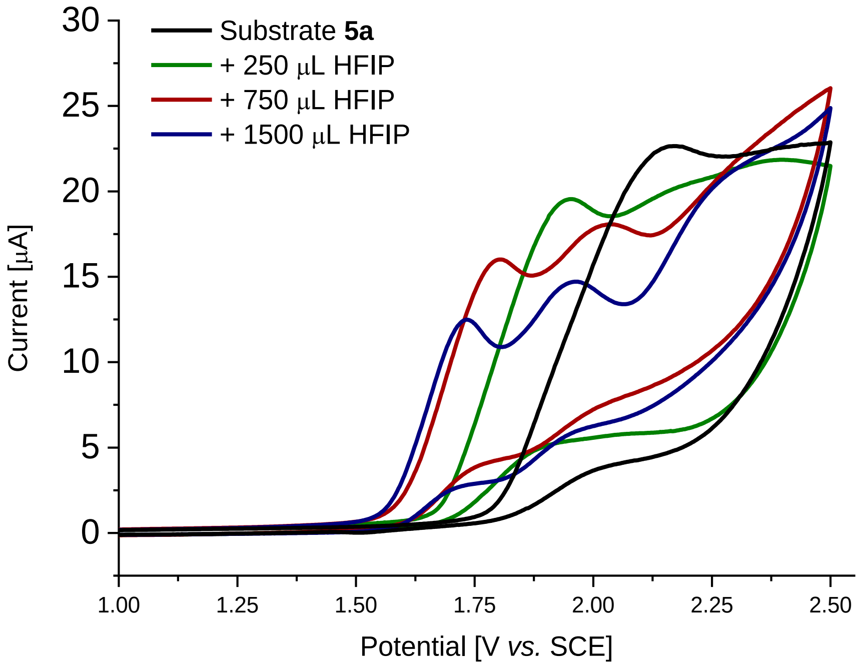

Figure 2 Cyclic voltammetry in DCE with *n*Bu_4NPF_6 (0.1 M) at 100 mV/s and glassy carbon working electrode. Substrate **5a** (black); substrate **5a** + 250 µL HFIP (DCE/HFIP = 12:1, green); substrate **5a** + 750 µL HFIP (DCE/HFIP = 4:1, red); substrate **5a** + 1500 µL HFIP (DCE/HFIP = 2:1, blue).

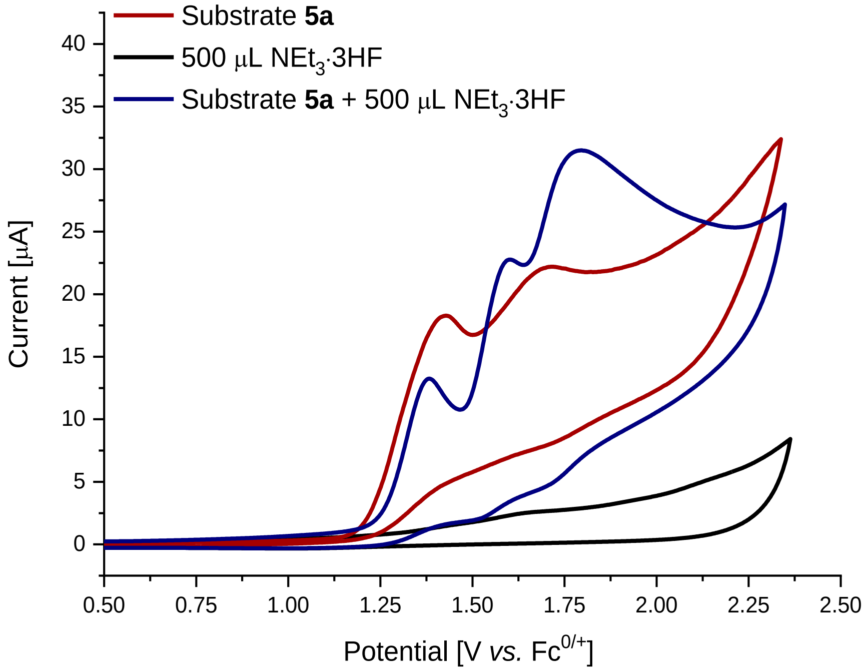

Figure 3 Cyclic voltammetry in DCE/HFIP (2:1) with *n*Bu_4NPF_6 (0.1 M) at 100 mV/s and glassy carbon working electrode. With NEt_3·3HF (black); substrate **5a** (red); substrate **5a** + 500 µL NEt_3·3HF (blue).

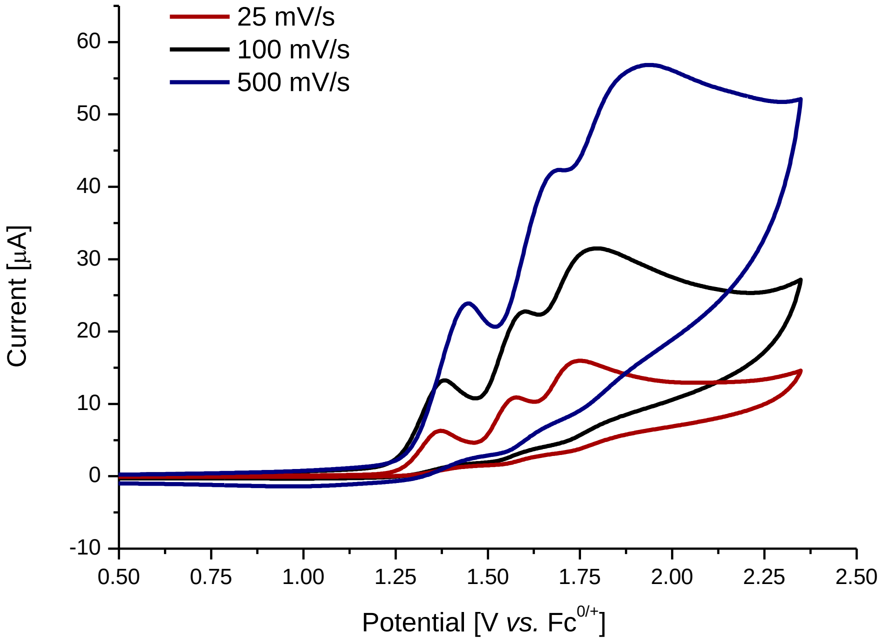

Figure 4 Cyclic voltammetry of substrate **5a** in DCE/HFIP (2:1), $NEt_3 \cdot 3HF$ (0.5 mL) with *n*Bu_4NPF_6 (0.1 M) and glassy carbon working electrode. 25 mV/s (red); 100 mV/s (black); 500 mV/s (blue).

5. Solar Energy-Enabled Fluorination

For the electrocatalysis powered by sunlight, a commercially available photovoltaic cell (Conrad Electronic SE, TPS-103 6 W, 17.5 V max. voltage, 428 mA max. current, 467 mm x 161 mm x 19 mm) was used. The output current was controlled with a customized and normalized constant current regulator and double checked with a multimeter.

The general procedure A-1 was followed using tridecylbenzene **1b** (131 mg, 0.50 mmol) on the 18th of March in 2022 at the rooftop of the *Institute of Organic and Biomolecular Chemistry* in Göttingen from 12.50–5.20 p.m. The weather conditions were sunny.

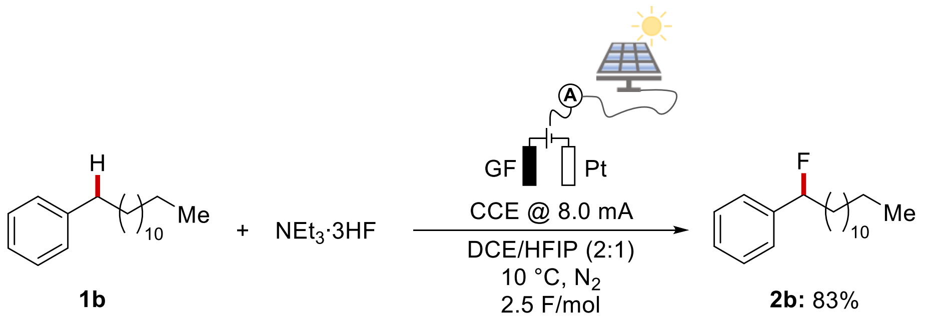

Scheme S1 C–H fluorination facilitated by solar energy. NMR yield with CH_2Br_2 as internal standard is provided.

Benzyl Fluoride C–H Shift: ^{1}H-NMR (300 MHz, C_6D_6) δ = 5.23 (ddd, J = 47.8, 8.2, 5.4 Hz). Calibrated ^{1}H-NMR yield from benzylic proton: 83%. **Benzylic Fluoride Shift:** ^{19}F{^{1}H}-NMR (282 MHz, C_6D_6) δ = –175.0. Calibrated ^{19}F{^{1}H}-NMR yield from benzylic fluoride: 87%. **HR-MS** (EI) *m/z* calc. for $C_{19}H_{31}F$ $[M]^+$: 278.2404, found: 278.2403.

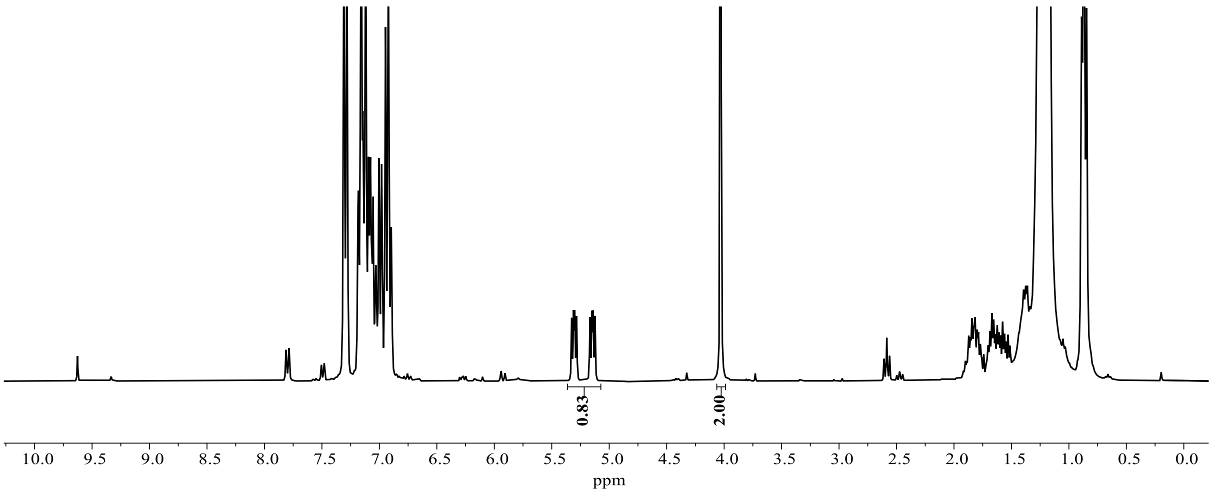

Figure 5 Crude ^{1}H-NMR Spectrum (300 MHz, C_6D_6) of the reaction mixture with CH_2Br_2 (36 µL, 0.50 mmol) as internal standard (4.03 ppm). The signals of the benzylic proton and internal standard are integrated.

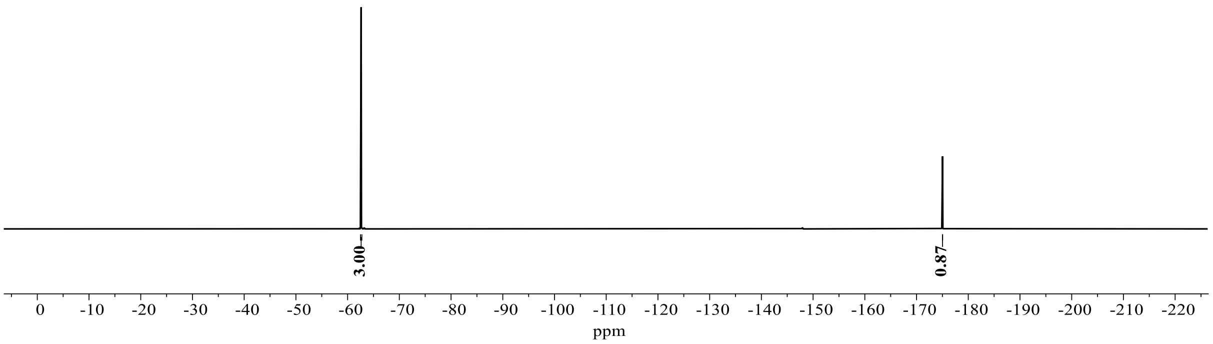

Figure 6 ^{19}F-NMR Spectrum (282 MHz, C_6D_6) of the reaction mixture with $PhCF_3$ (62 µL, 0.50 mmol) as internal standard (–62.6 ppm). The signals of the benzylic fluoride and internal standard are integrated.

6. Comparison with Previously Reported Conditions

To investigate the advantages of the developed strategy, substrates **1b** and **1v** were tested under the previously described reaction conditions.[3] According to the reported protocol, benzylic substrate **1** (0.25 mmol), grinded and oven-dried molecular sieves (20.0 mg), CsF (223 mg, 0.3 M), and MeCN/HFIP (4:1, 5.0 mL) were placed in a 10 mL undivided cell under inert atmosphere. Platinum electrodes (25 mm × 10 mm × 0.125 mm) were attached to the electrode holder which was assembled on the electrolysis cell. Electrosynthesis was performed at rt with a constant current of 8.0 mA until 2.5 F/mol were passed (2.1 h). After electrolysis, the reaction mixture was filtered over a plug of silica. The platinum electrodes were washed with EtOAc (5.0 mL) and the resulting fraction was filtered over the same silica plug. After rinsing the silica plug with an additional mixture of *n*hexane/ethylacetate (75 mL), the solvents were removed in vacuo. CH_2Br_2 (36 µL, 0.50 mmol) and $PhCF_3$ (62 µL, 0.50 mmol) were added to the resulting residue and the mixture was submitted for NMR analyses.

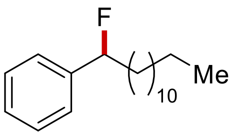

2b

The general procedure was followed using tridecylbenzene **1b** (65.1 mg, 0.25 mmol). Analysis of the NMR data indicated only trace formation of **2b**, next to hypothesized oxygenated side product **4b** (39%) and remaining starting material **1b** (47%).

Benzylic Fluoride Shift: Calibrated $^{19}F\{^1H\}$-NMR yield from benzylic fluoride: 2%.

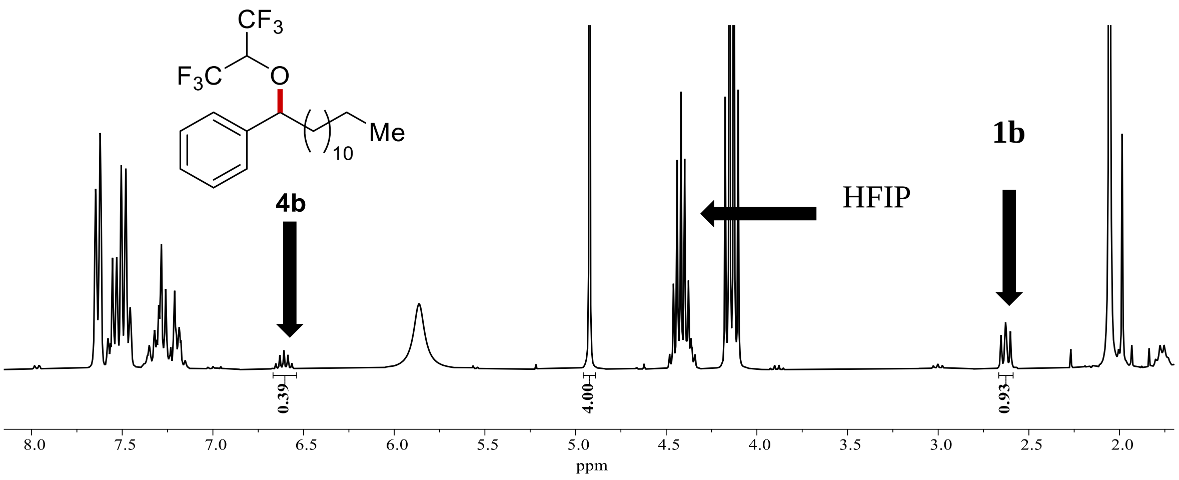

Figure 7 Crude ^1H-NMR Spectrum (300 MHz, $CDCl_3$) of the reaction mixture with CH_2Br_2 (36 µL, 0.50 mmol) as internal standard (4.93 ppm).

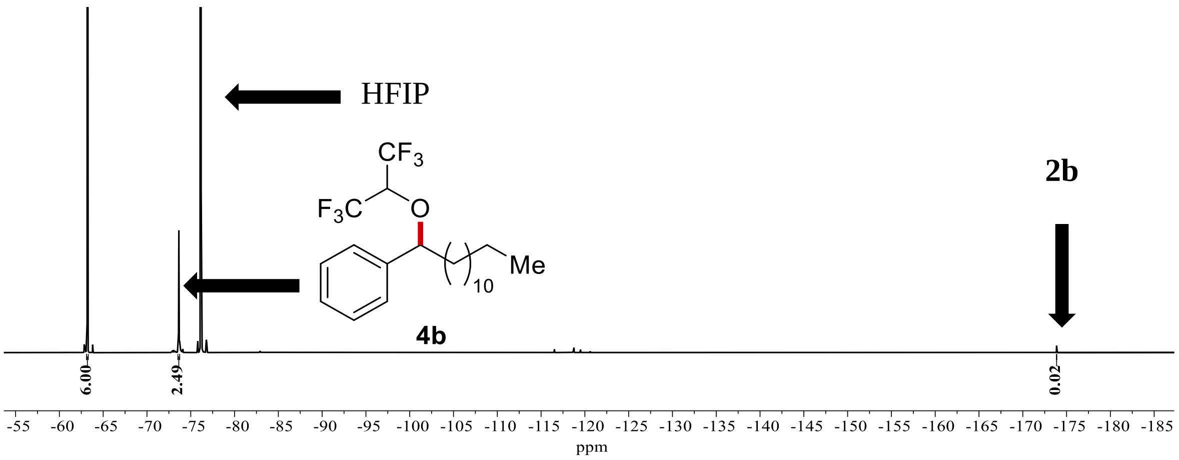

Figure 8 Crude $^{19}F\{^1H\}$-NMR Spectrum ($CDCl_3$, 300 MHz) of the reaction mixture with $PhCF_3$ (62 µL, 0.50 mmol) as internal standard (–62.7 ppm). The signals of the resolved (side) product(s) and internal standard are integrated.

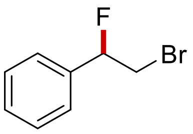

2v

The general procedure was followed using methyl (2-bromoethyl)benzene **1v** (46.3 mg, 0.25 mmol).

Analysis of the NMR data indicated only trace formation of **2v**, next to hypothesized oxygenated side product **4v** (38%) and remaining starting material **1v** (39%).

Benzylic Fluoride Shift: Calibrated $^{19}F\{^{1}H\}$-NMR yield from benzylic fluoride: 1%.

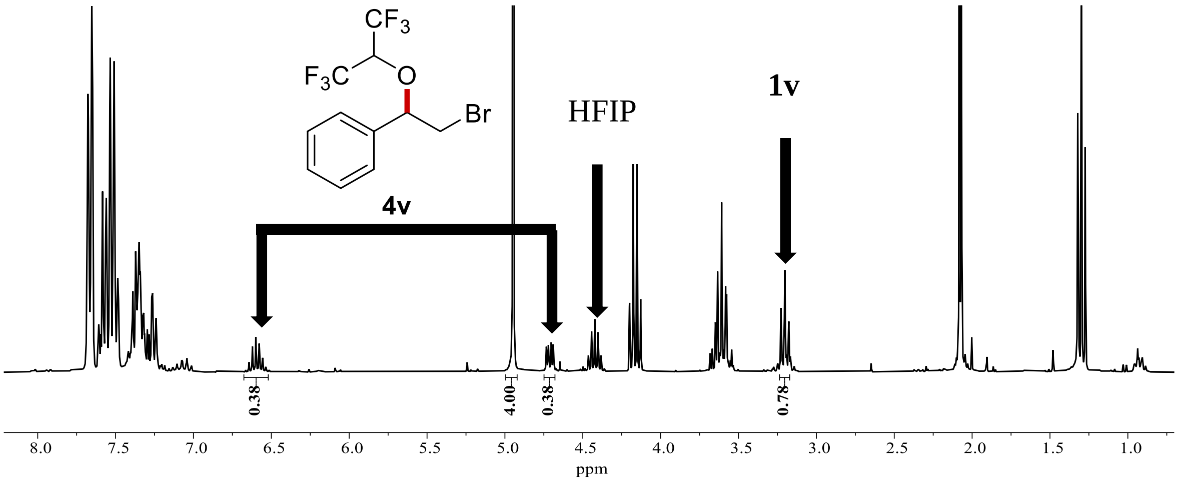

Figure 9 Crude ^{1}H-NMR Spectrum (300 MHz, $CDCl_3$) of the reaction mixture with CH_2Br_2 (36 µL, 0.50 mmol) as internal standard (4.93 ppm).

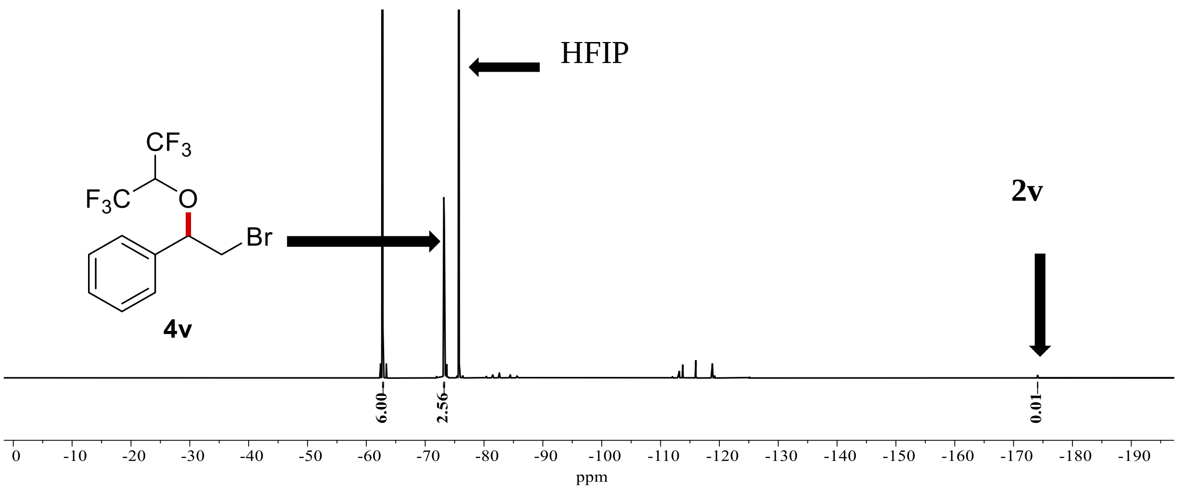

Figure 10 Crude $^{19}F\{^{1}H\}$-NMR Spectrum ($CDCl_3$, 300 MHz) of the reaction mixture with $PhCF_3$ (62 µL, 0.50 mmol) as internal standard (–62.7 ppm). The signals of the resolved (side) product(s) and internal standard are integrated.

7. H/D exchange experiment

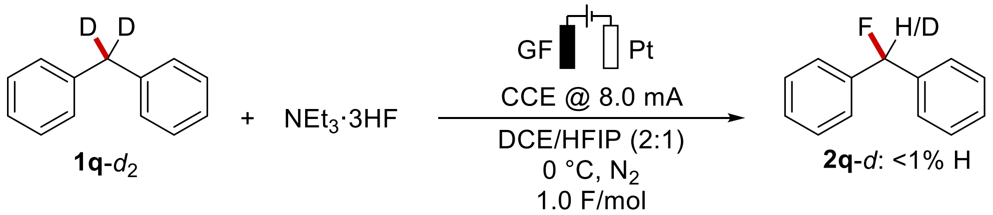

Diphenylmethane-d_2 **1q**-d_2 (85.0 mg, 0.50 mmol), DCE (2.0 mL), HFIP (1.0 mL) and $NEt_3 \cdot 3HF$ (1.0 mL) were placed in a 10 mL undivided cell under nitrogen atmosphere. A graphite felt (GF) anode (25mm × 10 mm × 6.0 mm) and a platinum cathode (25 mm × 10 mm × 0.125 mm) were attached to an electrode holder which was assembled on the electrolysis cell. Electrocatalysis was performed at 0 °C with a constant current of 8.0 mA for 1.7 h (1.0 F/mol). After electrolysis, the platinum cathode and the graphite felt anode were washed with DCM (Pt: 1 × 5.0 mL; C: 3 × 10 mL). The solvents were combined with the reaction mixture and diluted with *n*hexane (50 mL). The organic layers were washed three times with water (3 × 20 mL) in a separatory funnel and dried over Na_2SO_4. After adding silica gel (2.0 g), the mixture was filtered over a pad of celite, and the solvents were removed in vacuo. CH_2Br_2 (36 µL, 0.50 mmol) and $PhCF_3$ (62 µL, 0.50 mmol) were added to the residue and the mixture was submitted for NMR analyses. The spectral data are in accordance with those reported in literature.[4]

Benzyl Fluoride C–H Shift: no benzylic proton incorporation was observed in the ^{1}H-NMR spectrum. **Benzyl Fluoride C–D Shift:** ^{2}H-NMR (46 MHz, $CHCl_3$) δ = 6.55 (d, J = 7.4 Hz). Calibrated ^{2}H-NMR yield from benzylic deuteron 30%: **Benzylic Fluoride Shift:** ^{19}F{^{1}H}-NMR (282 MHz, $CDCl_3$) δ = −167.4 (t, J = 7.3 Hz). Calibrated ^{19}F{^{1}H}-NMR yield from benzylic fluoride 32%: **HR-MS** (EI) *m/z* calc. for $C_{12}H_{10}DF$ $[M]^+$: 187.0908, found: 187.0902.

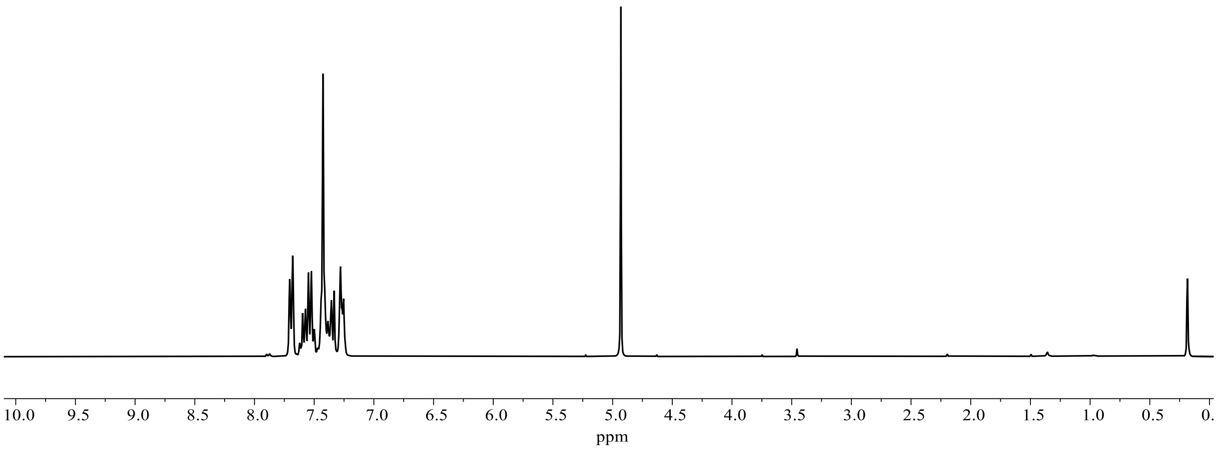

Figure 11 Crude ^{1}H-NMR Spectrum (300 MHz, $CDCl_3$) of the reaction mixture with CH_2Br_2 (36 µL, 0.50 mmol) as internal standard (4.93 ppm).

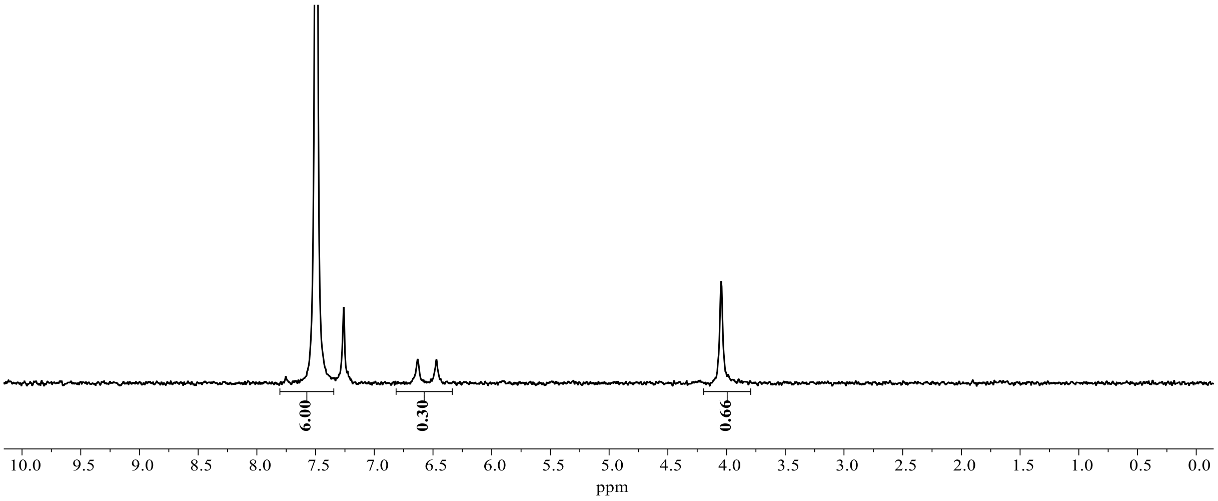

Figure 12 Crude ^{2}H-NMR Spectrum (46 MHz, $CHCl_3$) of the reaction mixture with C_6D_6 (46 µL, 0.50 mmol) as internal standard (7.49 ppm) and trace amounts of $CDCl_3$ (7.26 ppm). The signals of the benzylic deuteron of product and starting material and internal standard are integrated.

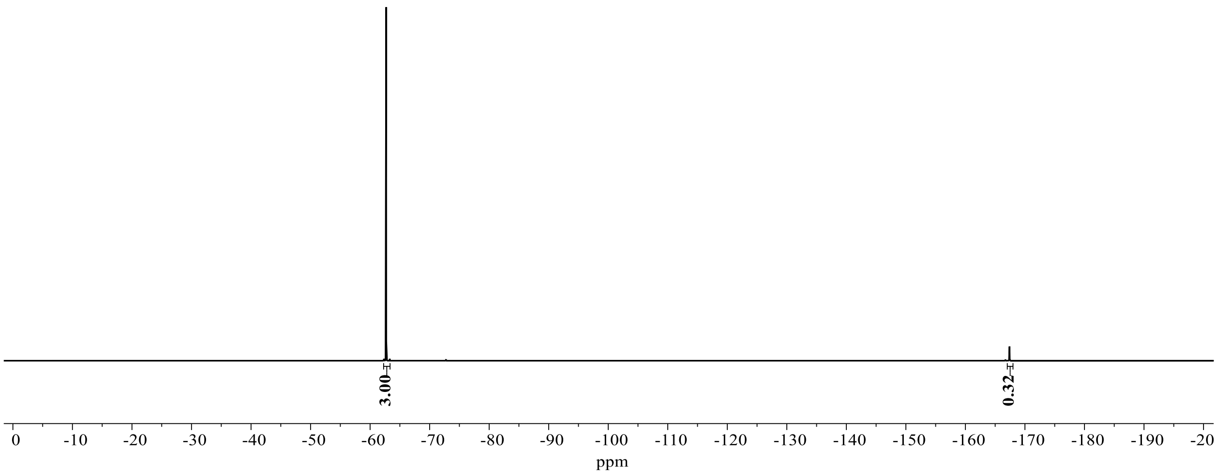

Figure 13 Crude $^{19}F\{^1H\}$-NMR Spectrum (282 MHz, $CDCl_3$) of the reaction mixture with $PhCF_3$ (62 µL, 0.50 mmol) as internal standard (–62.7 ppm). The signals of the benzylic fluoride and internal standard are integrated.

8. Kinetic Isotope Effect Studies

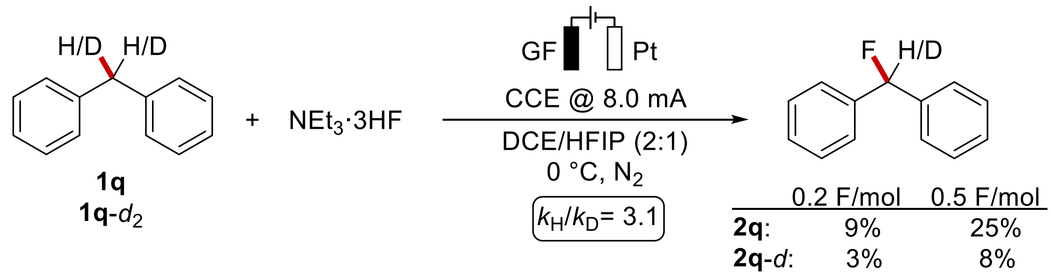

The experiment was performed twice, with different reaction times. Diphenylmethane **1q** (84.0 mg, 0.50 mmol) and diphenylmethane-d_2 **1q**-d_2 (85.0 mg, 0.50 mmol), DCE (2.0 mL), HFIP (1.0 mL) and NEt_3·3HF (1.0 mL) were placed in a 10 mL undivided cell under nitrogen atmosphere. A graphite felt (GF) anode (25 mm × 10 mm × 6.0 mm) and a platinum cathode (25 mm × 10 mm × 0.125 mm) were attached to an electrode holder which was assembled on the electrolysis cell. Electrocatalysis was performed at 0 °C with a constant current of 8.0 mA for 0.67 h (0.2 F/mol) and 1.67 h (0.5 F/mol), respectively. After electrolysis of each batch, the platinum cathode and the graphite felt anode were washed with DCM (Pt: 1 × 5.0 mL; C: 3 × 10 mL). The solvents were combined with the reaction mixture and diluted with *n*hexane (25 mL). The organic layers were washed three times with water (3 × 15 mL) in a separatory funnel and dried over Na_2SO_4. After adding silica gel (2.0 g), the mixture was filtered over celite, and the solvents were removed in vacuo. CH_2Br_2 (36 µL, 0.50 mmol) and $PhCF_3$ (62 µL, 0.50 mmol) and benzene-d_6 (46 µL, 0.50 mmol) were added to the residue and the mixture was submitted for NMR analyses.

Benzyl Fluoride C–H Shift: ^{1}H-NMR (300 MHz, $CHCl_3$) δ = 6.52 (d, J = 47.4 Hz). Calibrated ^{1}H-NMR yield of **2q** from benzylic proton: 9% (0.2 F/mol); 25% (0.5 F/mol). **Benzyl Fluoride C–D Shift:** ^{2}H-NMR (46 MHz, $CHCl_3$) δ = 6.55 (d, J = 7.4 Hz). Calibrated ^{2}H-NMR yield of **2l**-*d* from benzylic deuteron 3% (0.2 F/mol); 8% (0.5 F/mol): **Benzylic Fluoride Shift:** ^{19}F{^{1}H}-NMR (282 MHz, $CDCl_3$) δ = −166.7 (s), −167.4 (t, J = 7.3 Hz). Calibrated ^{19}F{^{1}H}-NMR yields from benzylic fluorides: 9% for **2q**, 3% for **2q**-*d* (0.2 F/mol); 26% for **2q**, 8% for **2q**-*d* (0.5 F/mol). The spectral data are in accordance with those reported in literature.[4] Accordingly, a KIE value of $k_H/k_D \approx 3.1$ was determined by the spectroscopic analyses.

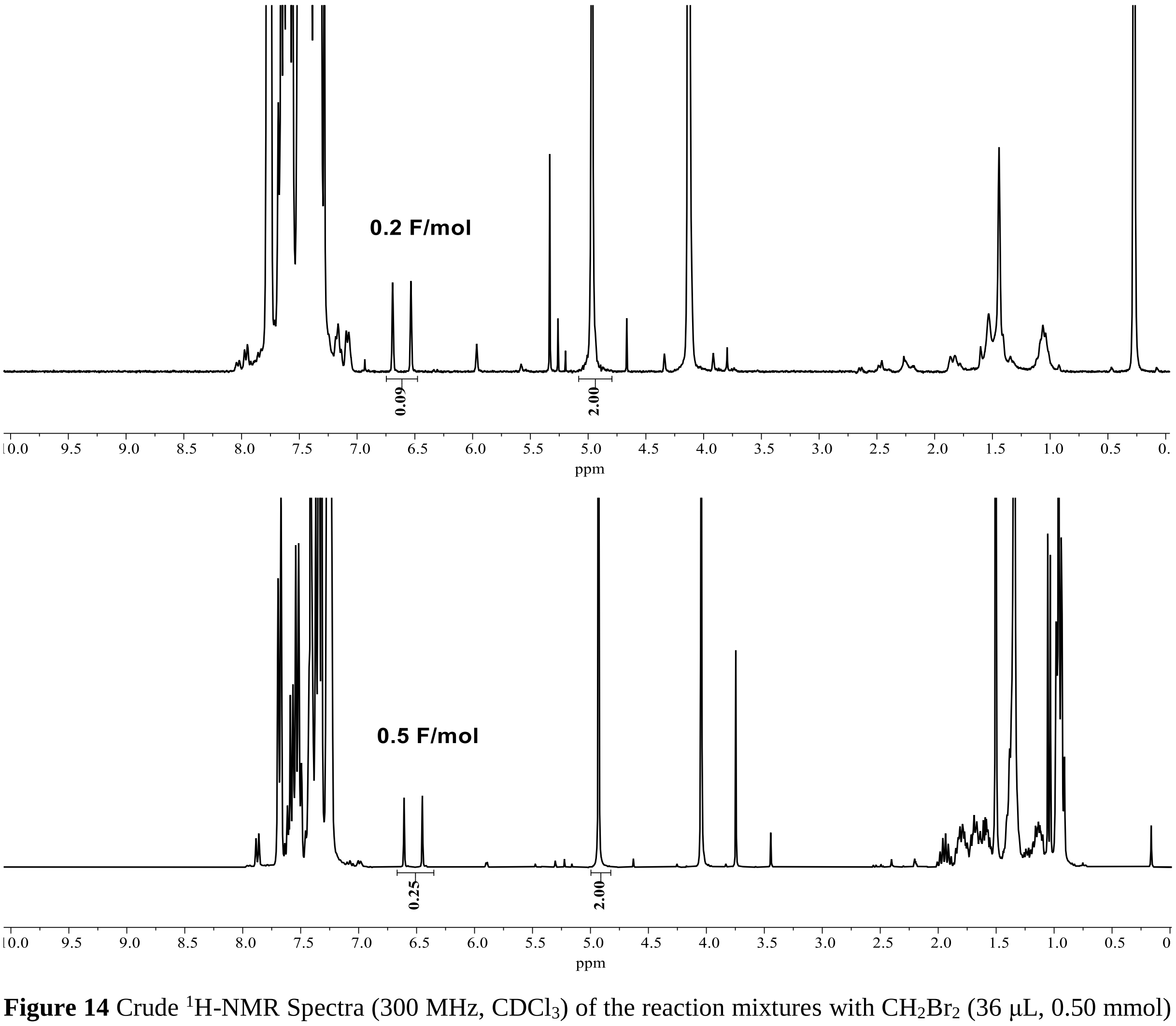

Figure 14 Crude ^{1}H-NMR Spectra (300 MHz, $CDCl_3$) of the reaction mixtures with CH_2Br_2 (36 µL, 0.50 mmol) as internal standard (4.93 ppm). The signals of the benzylic deuteron of product and internal standard are integrated.

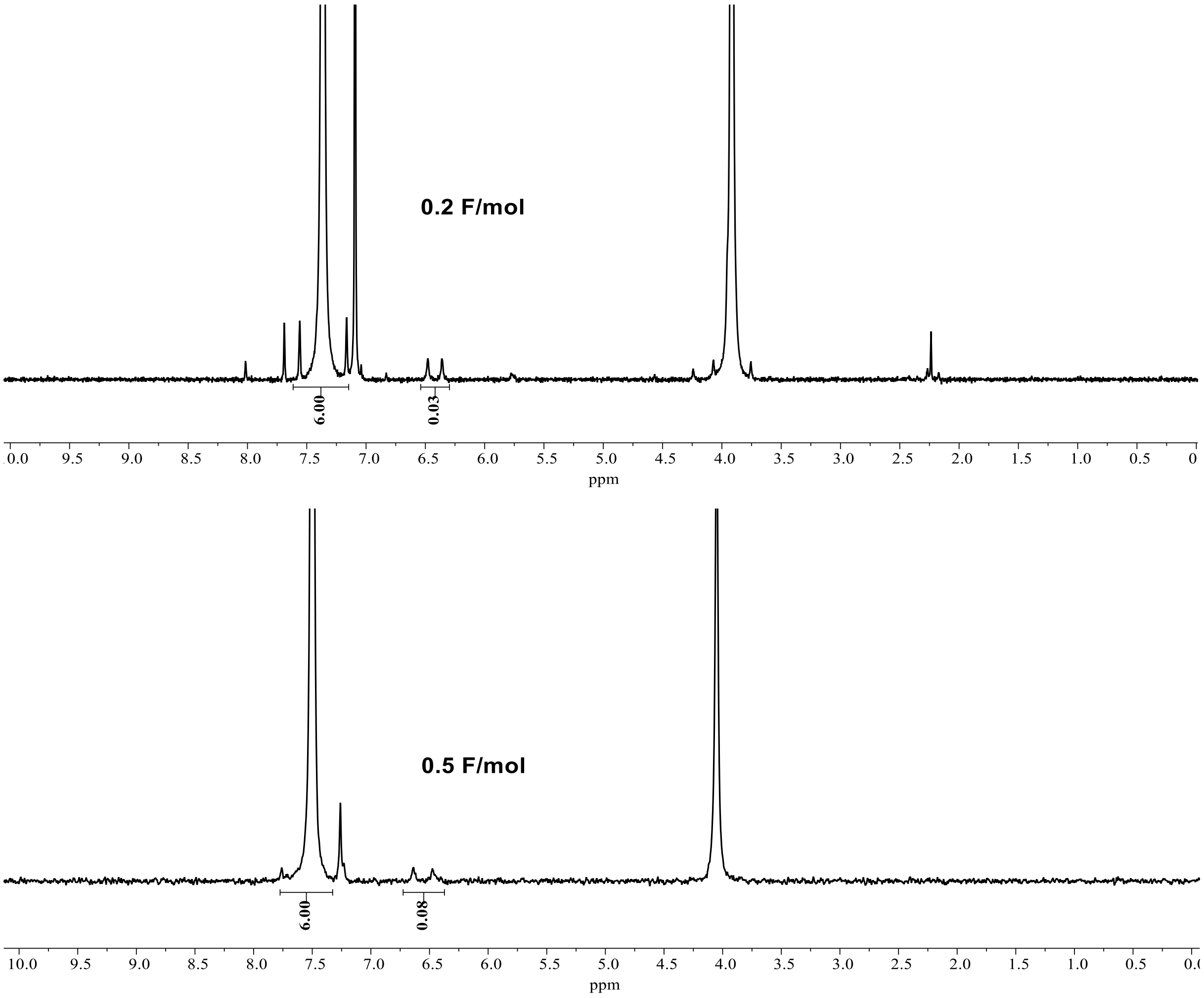

Figure 15 Crude ^{2}H-NMR Spectra (46 MHz, $CHCl_3$) of the reaction mixtures with C_6D_6 (46 µL, 0.50 mmol) as internal standard (7.49 ppm) and trace amounts of $CDCl_3$ (7.26 ppm). The signals of the benzylic deuteron of product and internal standard are integrated.

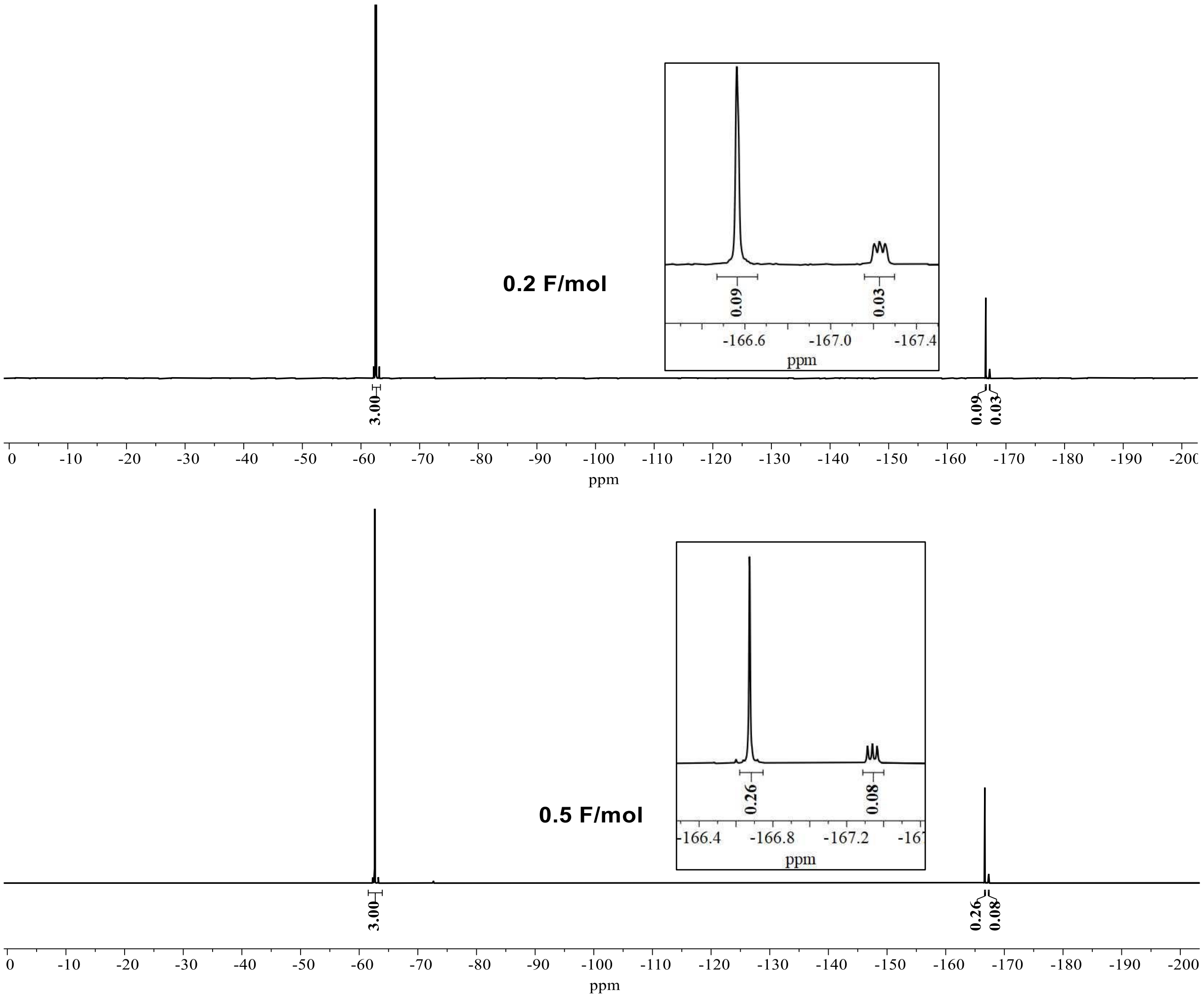

Figure 16 Crude $^{19}F\{^{1}H\}$-NMR Spectra (282 MHz, $CDCl_3$) of the reaction mixtures with $PhCF_3$ (62 µL, 0.50 mmol) as internal standard (–62.7 ppm). The signals of the benzylic fluoride and internal standard are integrated.

9. Gram-Scale Reaction

Caution! Working with HF and sources thereof is highly dangerous. These reagents are highly toxic, and they easily penetrate tissue, leading potentially to irreversible damage. Therefore, reagents should be handled only by trained staff and under appropriate safety measurements (lab coat, impenetrable gloves, and eye protection) inside a well-ventilated fume hood. Glassware should not be exposed to these reagents for unnecessarily long times, as HF can damage glassware. Alternatively inert polymer-based vessels for the reaction and the workup procedure can be used. The present gram-scale synthesis was set up analogous to the reactions performed on 0.5 mmol scale, but with bigger reaction vessel and electrodes (Figure 17).

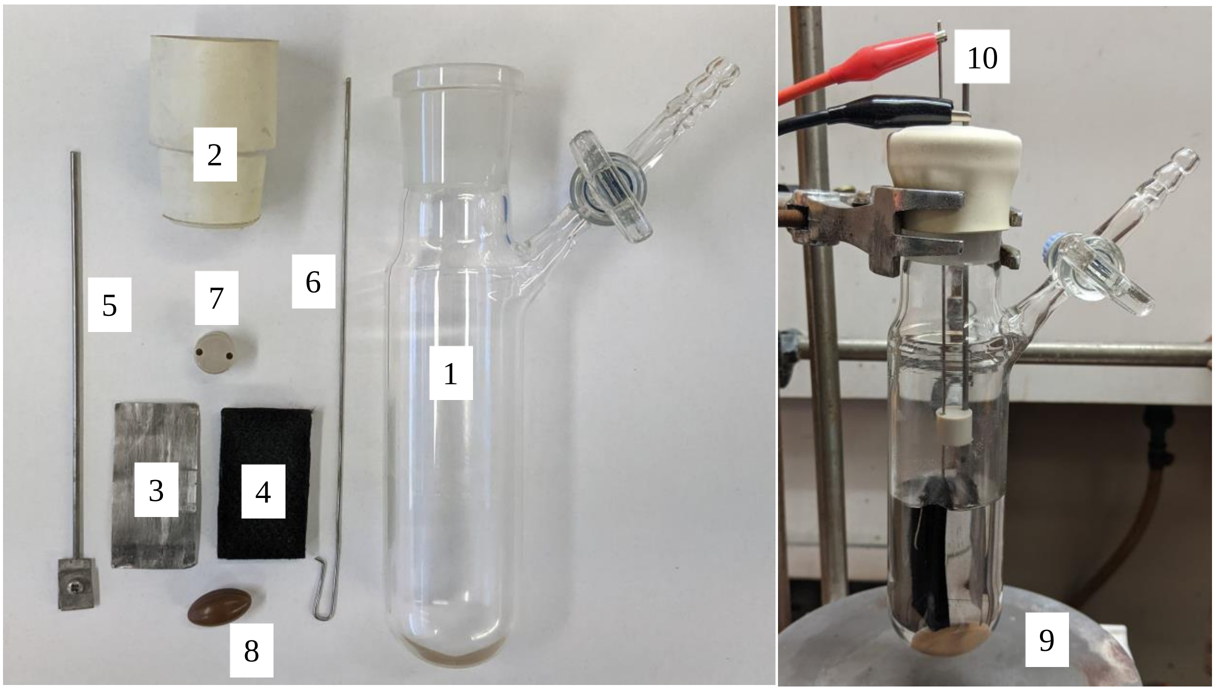

Figure 17 Left: Assembly parts for the undivided cell used for gram-scale electrolysis; 1 – reaction vessel (100 mL NS 29 Schlenk tube), 2 – NS 29 septum with piercings for electrode holders, 3 – Pt cathode, 4 – GF anode, 5 – stainless steel cathode holder, 6 – stainless steel anode holder, 7 – electrode spacer, 8 – stirring bar. Right: Fully assembled cell above stirring plate (9), with alligator clamp connectors (10). The electrodes are arranged plane parallel with approx. 1 cm distance.

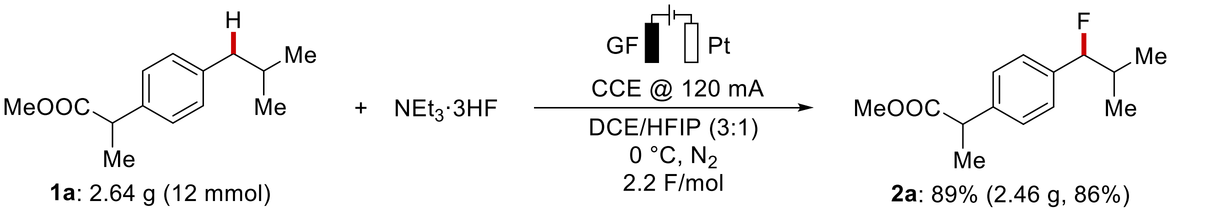

Methyl 2-(4-(2-methylpropyl)phenyl)propanoate **1a** (2.64 g, 12.0 mmol), DCE (24 mL), HFIP (8.0 mL) and $NEt_3 \cdot 3HF$ (8.0 mL) were placed in a 100 mL undivided cell under nitrogen atmosphere. A graphite felt (GF) anode (45 mm × 25 mm × 6.0 mm) and a platinum cathode (50 mm × 25 mm × 0.25 mm) were attached to an electrode holder which was assembled on the electrolysis cell. Electrocatalysis was performed at 0 °C with a constant current of 120 mA for 5.9 h (2.2 F/mol). After electrolysis, the platinum cathode and the graphite felt anode were washed with DCM (Pt: 1 × 5.0 mL; C: 3 × 15.0 mL). The solvents were combined with the reaction mixture and diluted with *n*hexane (150 mL). The combined organic layers were washed three times with water (3 × 75 mL) and dried over Na_2SO_4. After adding silica gel (15 g), the mixture was filtered over celite, and the solvents were removed in vacuo. CH_2Br_2 (86 µL, 1.20 mmol) and $PhCF_3$ (148 µL, 1.20 mmol) were added to the residue and the mixture was submitted for NMR analyses. After conducting the crude NMR analysis, the solvents were removed in vacuo, and the residue was purified by column chromatography (*n*hexane/EtOAc = 25/1), to obtain **2a** (2.46 g, 10.3 mmol, 86%) as a colorless oil.

¹H-NMR (400 MHz, $CDCl_3$, mixture of diastereomers): δ = 7.34 (d, *J* = 8.2, 2H), 7.29 (d, *J* = 8.1, 2H), 5.12 (dd, *J* = 47.0, 6.8, 1H), 3.78 (q, *J* = 7.2, 1H), 3.71 (s, 3H), 2.23–2.05 (m, 1H), 1.55 (d, *J* = 7.2, 3H), 1.06 (d, *J* = 6.6, 3H), 0.90 (d, *J* = 6.9, 3H). Calibrated ^{1}H-NMR yield from benzylic proton: 89%. **¹³C-NMR** (101 MHz, $CDCl_3$, mixture of diastereomers): δ = 175.0 (C_q), 140.5 (d, $^5J_{C-F}$ = 1.8, C_q), 138.4 (d, $^2J_{C-F}$ = 20.6, C_q), 127.5 (CH), 126.6 (d, $^3J_{C-F}$ = 7.0, CH), 99.2 (d, $^1J_{C-F}$ = 173.5, CH), 52.2 (CH_3), 45.3 (CH), 34.4 (d, $^2J_{C-F}$ = 22.8, CH), 18.7 (CH_3) 18.5 (d, $^3J_{C-F}$ = 5.6, CH_3), 17.7 (d, $^3J_{C-F}$ = 5.2, CH_3). **¹⁹F{1H}-NMR** (282 MHz, $CDCl_3$, mixture of diastereomers) δ = –179.6, –179.6. Calibrated ^{19}F{^{1}H}-NMR yield from benzylic fluoride: 89%. **IR** (ATR): $\tilde{\nu}$ = 2978, 2876, 1736, 1514, 1207, 1161, 990, 840. **HR-MS** (EI) *m/z* calc. for $C_{14}H_{19}FO_2$ $[M]^+$: 238.1364, found: 238.1362. The spectral data are in accordance with those reported in literature. [5]

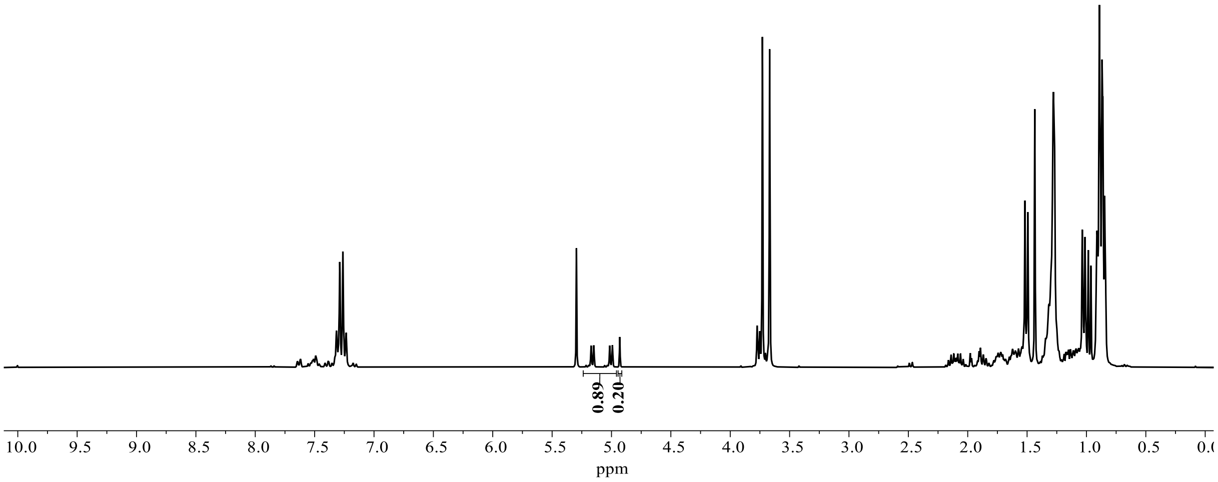

Figure 18 Crude ^{1}H-NMR Spectrum (300 MHz, $CDCl_3$) of the reaction mixture with CH_2Br_2 (86 µL, 1.20 mmol) as internal standard (4.93 ppm).

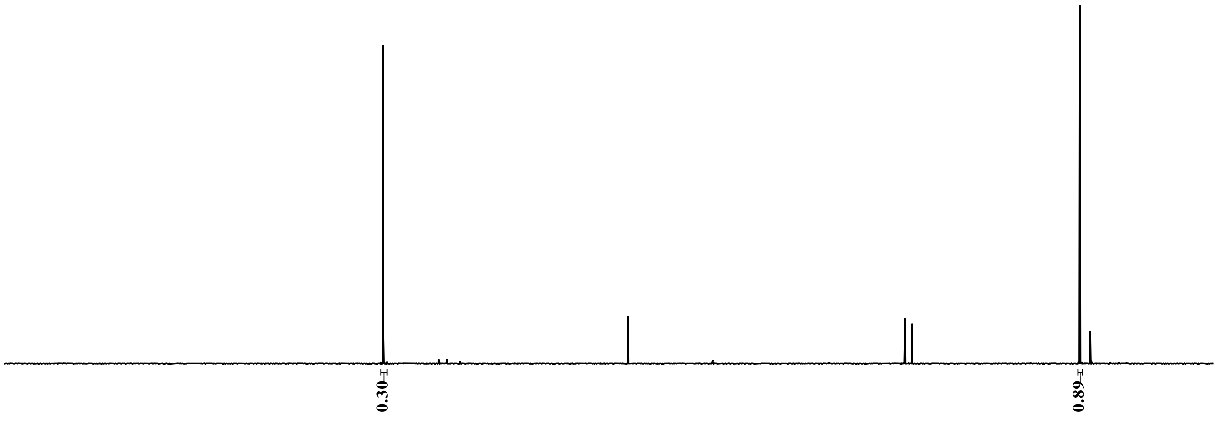

Figure 19 Crude ^{19}F{^{1}H}-NMR Spectrum (282 MHz, $CDCl_3$) of the reaction mixture with $PhCF_3$ (148 µL, 1.20 mmol) as internal standard (–62.7 ppm). The signals of the benzylic fluoride and internal standard are integrated.

10. Quantitative ^{1}H and $^{19}F\{H\}$-NMR Spectra for Benzyl Fluorides

Prior to quantification, the automatic baseline correction and phase correction was performed using MNOVA 14.2.0 from Mestrelabs. Relevant integrals of nuclei from products and the standard are shown in the spectra. For further details see the captions and information given in the spectra.

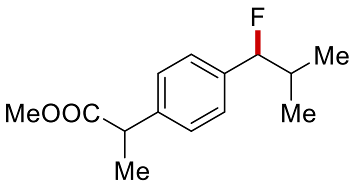

2a

Methyl 2-(4-(-1-fluoro-2-methylpropyl)phenyl)propanoate

The general procedure A-1 was followed using methyl 2-(4-(2-methylpropyl)phenyl)propanoate **1a** (110 mg, 0.50 mmol).

Benzyl Fluoride C–H Shift: ^{1}H-NMR (300 MHz, $CDCl_3$, mixture of diastereomers) δ = 5.11 (dd, J = 47.0, 6.8 Hz). Calibrated ^{1}H-NMR yield from benzylic proton: 92%. **Benzylic Fluoride Shift:** ^{19}F{^{1}H}-NMR (282 MHz, $CDCl_3$, mixture of diastereomers) δ = –179.6, –179.7. Calibrated ^{19}F{^{1}H}-NMR yield from benzylic fluoride: 92%. **HR-MS** (EI) *m/z* calc. for $C_{14}H_{19}FO_2$ $[M]^+$: 238.1364, found: 238.1362.

The spectral data are in accordance with those reported in literature.[5]

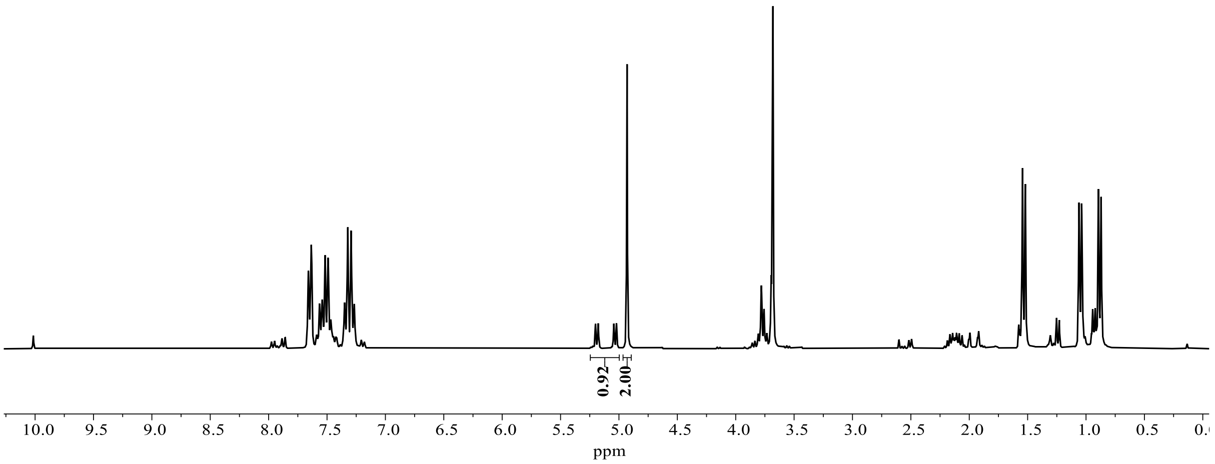

Figure 20 Crude ^{1}H-NMR Spectrum ($CDCl_3$, 300 MHz) of the reaction mixture with CH_2Br_2 (36 µL, 0.50 mmol) as internal standard (4.93 ppm). The signals of the resolved product and internal standard are integrated.

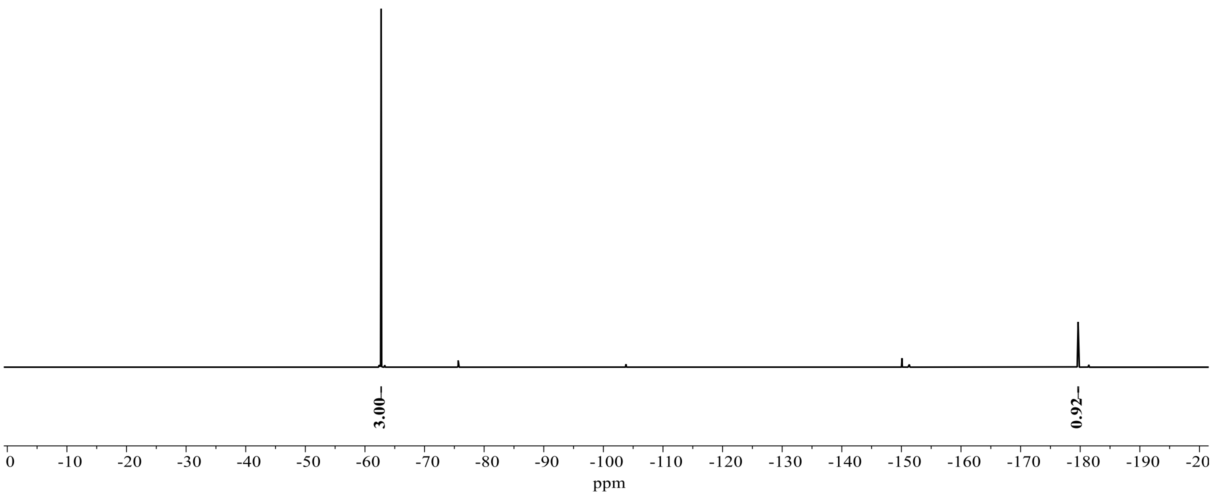

Figure 21. Crude $^{19}F\{^1H\}$-NMR Spectrum ($CDCl_3$, 300 MHz) of the reaction mixture with $PhCF_3$ (62 µL, 0.50 mmol) as internal standard (–62.7 ppm). The signals of the resolved product and internal standard are integrated.

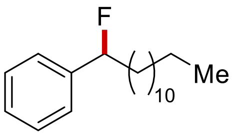

2b

1-Fluorotridecylbenzene

The general procedure A-1 was followed using tridecylbenzene **1b** (131 mg, 0.50 mmol). After conducting the NMR analysis, the solvents were removed in vacuo, and the residue was purified by column chromatography (*n*hexane) to obtain the product **2b** as a colorless oil (90.4 mg, 0.32 mmol, 65%).

^{1}H-NMR (400 MHz, $CDCl_3$): δ = 7.42–7.31 (m, 5H), 5.47 (ddd, J = 48.1, 5.1, 4.9 Hz, 1H), 1.99 (m, 1H), 1.91–1.74 (m, 1H), 1.55–1.41 (m, 2H), 1.29 (m, 18H), 0.91 (t, J = 6.7 Hz, 3H). Calibrated ^{1}H-NMR yield from benzylic proton: 82%. **^{13}C-NMR** (101 MHz, $CDCl_3$): δ = 140.8 (d, $^2J_{C–F}$ = 19.8 Hz, C_q), 128.5 (CH), 128.3 (d, $^4J_{C–F}$ = 1.9 Hz, CH), 125.7 (d, $^3J_{C–F}$ = 6.8 Hz, CH), 94.8 (d, $^1J_{C–F}$ = 170.1 Hz, CH), 37.4 (d, $^2J_{C–F}$ = 23.5 Hz, CH_2), 32.1 (CH_2), 29.8 (CH_2), 29.8 (CH_2), 29.7 (CH_2), 29.6 (CH_2), 29.5 (CH_2), 29.5 (CH_2), 25.3 (d, $^3J_{C–F}$ = 4.3 Hz, CH_2), 22.8 (CH_2), 14.3 (CH_3). **^{19}F{^{1}H}-NMR** (282 MHz, $CDCl_3$): δ = –174.2. Calibrated $^{19}F\{^1H\}$-NMR yield from benzylic fluoride: 86%. **IR** (ATR): $\tilde{\nu}$ = 2922, 2853, 1458, 1372, 1026, 975, 913, 753, 697, 551. **HR-MS** (EI) *m/z* calc. for $C_{19}H_{31}F$ $[M]^+$: 278.2404, found: 278.2404.

The spectral data are in accordance with those reported in literature.[6]

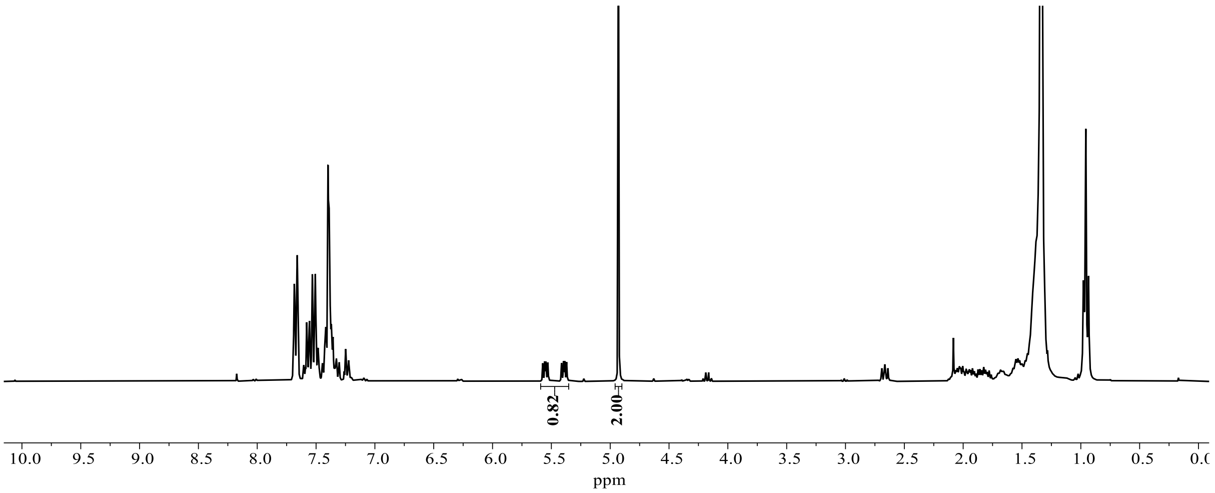

Figure 22 Crude ^{1}H-NMR Spectrum (300 MHz, $CDCl_3$) of the reaction mixture with CH_2Br_2 (36 µL, 0.50 mmol) as internal standard (4.93 ppm). The signals of the benzylic proton and internal standard are integrated.

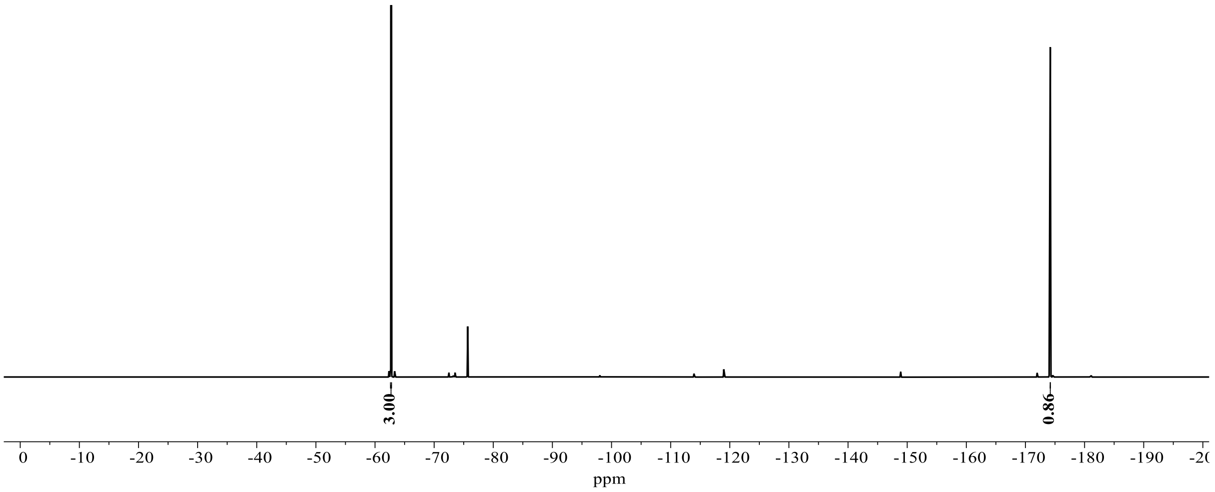

Figure 23 ^{19}F-NMR Spectrum (282 MHz, $CDCl_3$) of the reaction mixture with $PhCF_3$ (62 µL, 0.50 mmol) as internal standard (–62.7 ppm). The signals of the benzylic fluoride and internal standard are integrated.

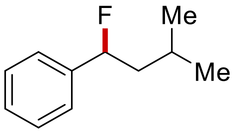

2c

(1-Fluoro-3-methylbutyl)benzene

The general procedure A-1 was followed using isopentylbenzene **1c** (74.1 mg, 0.50 mmol).

Benzyl Fluoride C–H Shift: ^{1}H-NMR (300 MHz, $CDCl_3$) δ = 5.55 (ddd, J = 48.6, 4.3, 4.2 Hz). Calibrated ^{1}H-NMR yield from benzylic proton: 70%. **Benzylic Fluoride Shift:** ^{19}F{^{1}H}-NMR (282 MHz, $CDCl_3$) δ = –174.3. Calibrated ^{19}F{^{1}H}-NMR yield from benzylic fluoride: 72%. **HR-MS** (EI) *m/z* calc. for $C_{11}H_{15}F$ $[M]^+$: 166.1152, found: 166.1152.

The spectral data are in accordance with those reported in literature.[7]

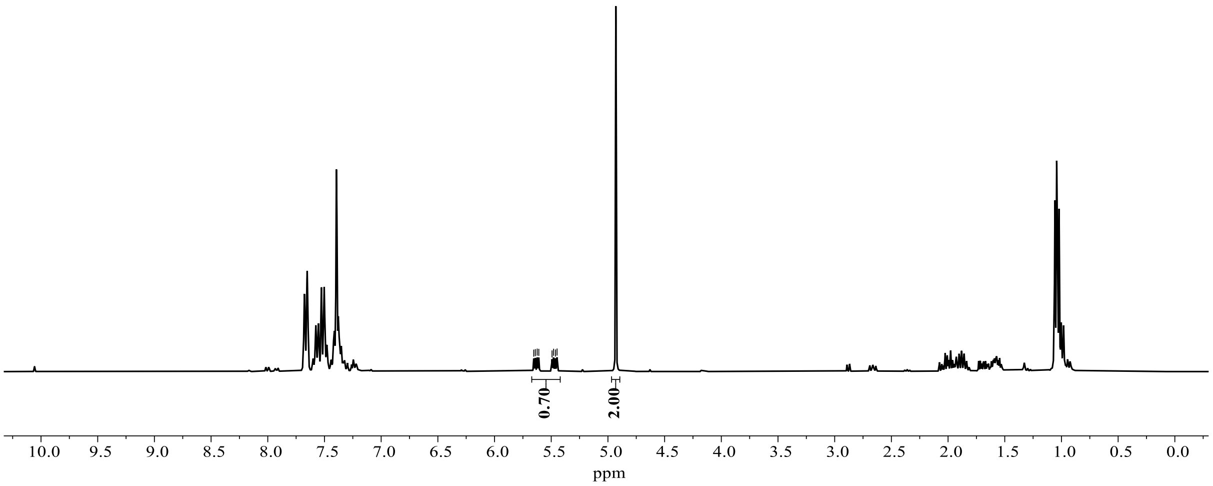

Figure 24 Crude ^{1}H-NMR Spectrum (300 MHz, $CDCl_3$) of the reaction mixture with CH_2Br_2 (36 µL, 0.50 mmol) as internal standard (4.93 ppm). The signals of the benzylic proton and internal standard are integrated.

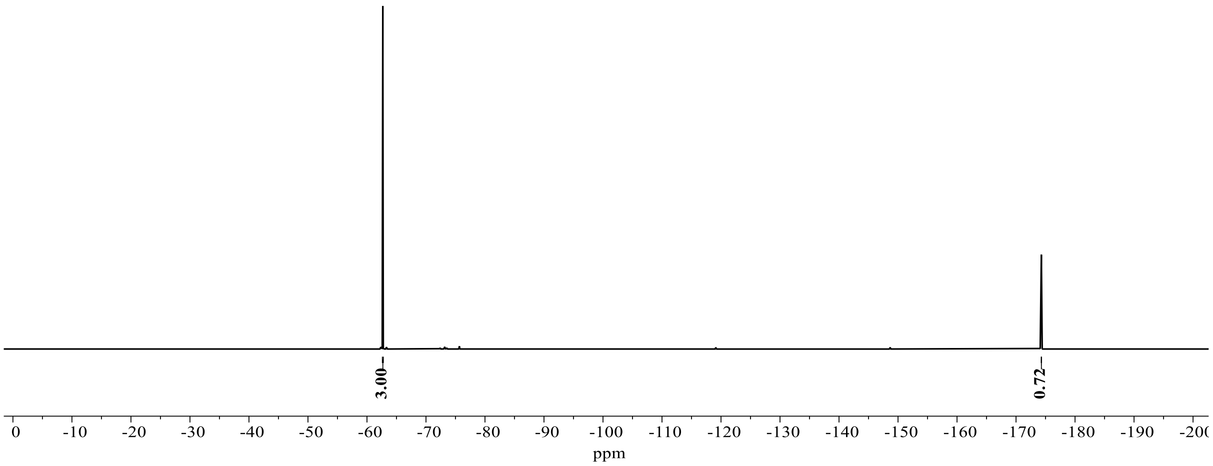

Figure 25 ^{19}F-NMR Spectrum (282 MHz, $CDCl_3$) of the reaction mixture with $PhCF_3$ (62 µL, 0.50 mmol) as internal standard (–62.7 ppm). The signals of the benzylic fluoride and internal standard are integrated.

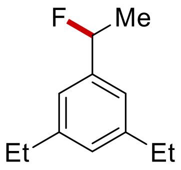

2d

1,3-Diethyl-5-(1-fluoroethyl)benzene

The general procedure A-2 was followed using 1,3,5-triethylbenzene **1d** (81.3 mg, 0.50 mmol) at 0 °C. After conducting the NMR analysis, the solvents were removed in vacuo, and the residue was purified by column chromatography on partially neutralized silica (*n*hexane/EtOAc = 40:1) to obtain the product **2d** as a colorless oil (54.4 mg, 0.30 mmol, 60%).

^{1}H-NMR (300 MHz, $CDCl_3$): δ = 7.01 (s, 3H), 5.59 (dq, J = 47.8, 6.4 Hz, 1H), 2.65 (q, J = 7.6 Hz, 4H), 1.65 (dd, J = 23.9, 6.4 Hz, 3H), 1.25 (t, J = 7.6 Hz, 6H). Calibrated ^{1}H-NMR

(300 MHz, CD_2Cl_2) yield from benzylic proton: 78%. **^{19}F{^{1}H}-NMR** (282 MHz, $CDCl_3$) −165.9. Calibrated ^{19}F{^{1}H}-NMR (282 MHz, CD_2Cl_2) yield from benzylic fluoride: 75%. **^{13}C-NMR** (101 MHz, $CDCl_3$): δ = 144.7 (C_q), 141.6 (d, $^2J_{C–F}$ = 19.2 Hz, C_q), 127.6 (d, $^5J_{C–F}$ = 2.1 Hz, CH), 122.3 (d, $^3J_{C–F}$ = 6.5 Hz, CH), 91.4 (d, $^1J_{C–F}$ = 166.8 Hz, CH), 29.0 (CH_2), 23.1 (d, $^2J_{C–F}$ = 25.4 Hz, CH_3), 15.7 (CH_3). **IR** (ATR): $\tilde{\nu}$ = 2968, 2934, 2873, 1606, 1460, 1374, 1083, 1060, 870, 847, 710. **HR-MS** (EI) *m/z* calc. for $C_{12}H_{17}F$ $[M]^+$: 180.1314, found: 180.1309.

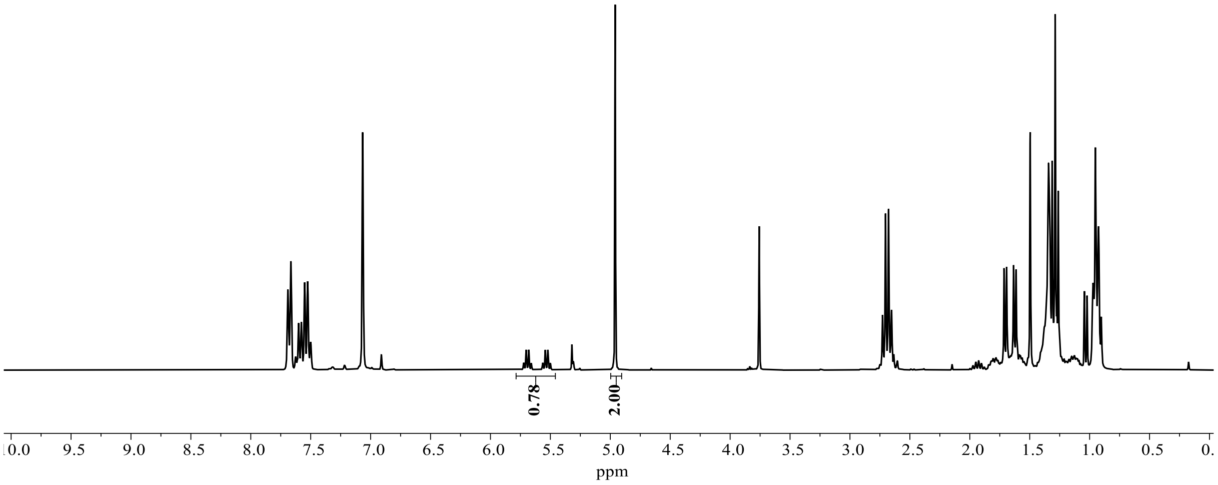

Figure 26 Crude ^{1}H-NMR Spectrum (300 MHz, CD_2Cl_2) of the reaction mixture with CH_2Br_2 (36 µL, 0.50 mmol) as internal standard (4.96 ppm). The signals of the benzylic proton and internal standard are integrated.

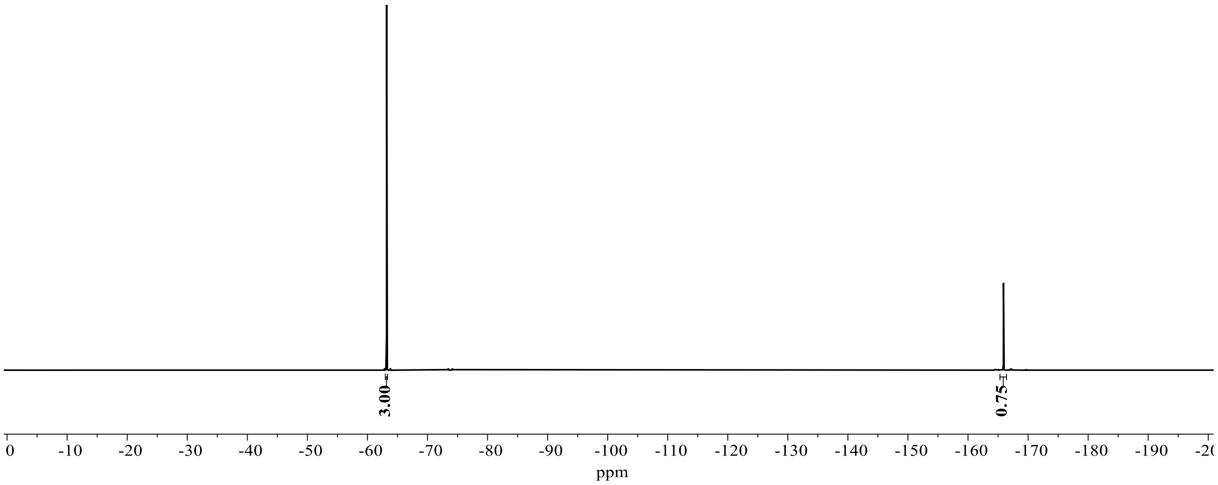

Figure 27 Crude ^{19}F{^{1}H}-NMR Spectrum (282 MHz, CD_2Cl_2) of the reaction mixture with $PhCF_3$ (62 µL, 0.50 mmol) as internal standard (–63.2 ppm). The signals of the benzylic fluoride and internal standard are integrated.

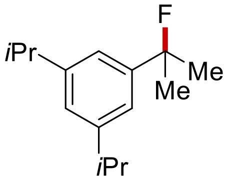

2e

1-(2-fluoropropan-2-yl)-3,5-diisopropylbenzene

The general procedure A-1 was followed using 1,3,5-triisopropylbenzene **1e** (102 mg, 0.50 mmol) at 0 °C. After filtration over a silica plug (*n*hexane/DCM = 95:5), the organic layer was washed with water (3 x 50 mL) and dried over Na_2SO_4. The solvents were removed in vacuo and the resulting residue was analyzed by NMR.

Aromatic C–H Shift: ^{1}H-NMR (300 MHz, $CDCl_3$): δ = 7.11–7.07 (m). Calibrated ^{1}H-NMR yield from aromatic proton: 65%. **Benzylic Fluoride Shift:** ^{19}F{^{1}H}-NMR (282 MHz, $CDCl_3$) δ = –136.4. Calibrated ^{19}F{^{1}H}-NMR yield from benzylic fluoride: 63%. **HR-MS** (EI) *m/z* calc. for $C_{15}H_{23}F$ [M]$^+$: 222.1778, found: 222.1775.

The spectral data are in accordance with those reported in literature.[8]

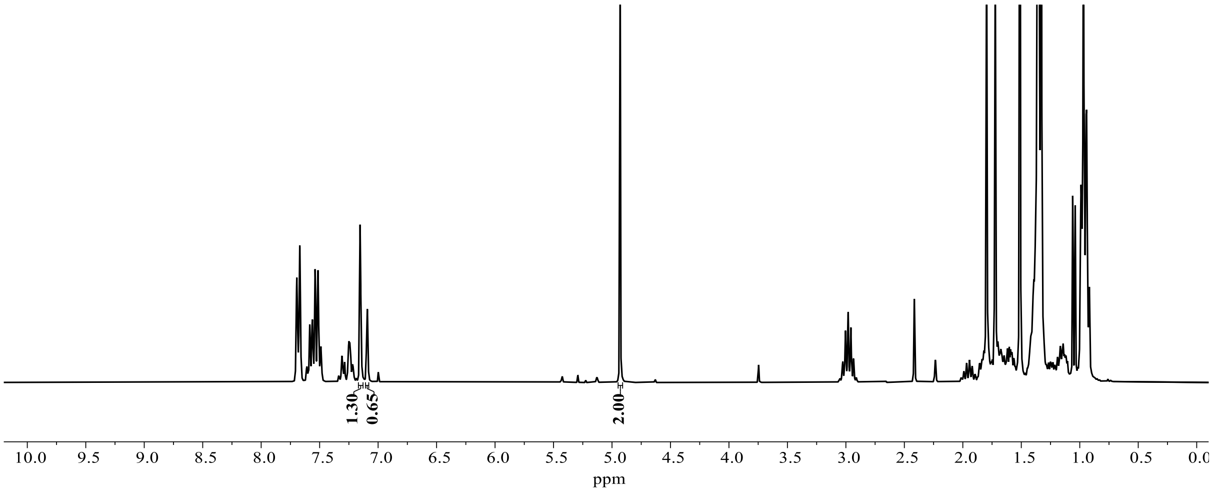

Figure 28 Crude ^{1}H-NMR Spectrum (300 MHz, $CDCl_3$) of the reaction mixture with CH_2Br_2 (36 µL, 0.50 mmol) as internal standard (4.94 ppm). The signals of the aromatic protons and internal standard are integrated.

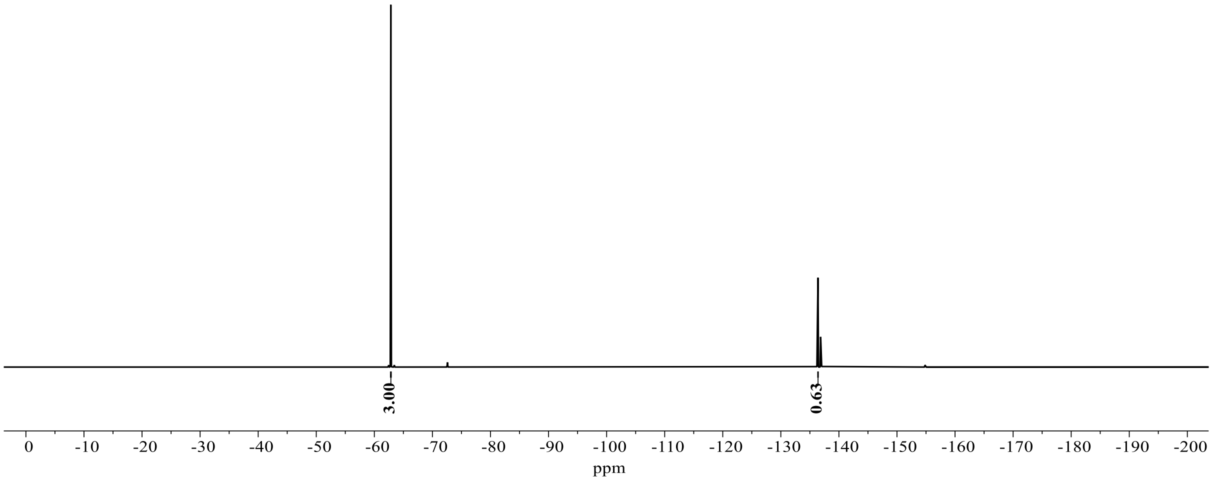

Figure 29 ^{19}F-NMR Spectrum (282 MHz, $CDCl_3$) of the reaction mixture with $PhCF_3$ (62 µL, 0.50 mmol) as internal standard (–62.7 ppm). The signals of the benzylic fluoride and internal standard are integrated.

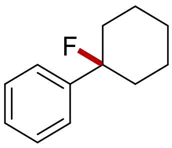

2f

(1-Fluorocyclohexyl)benzene

The general procedure A was followed using cyclohexylbenzene **1f** (80.1 mg, 0.50 mmol) at 0 °C. After conducting the NMR analysis, the solvents were removed in vacuo, and the residue was purified by column chromatography on partially neutralized silica (*n*hexane) to obtain the product **2f** as a colorless oil (32.0 mg, 0.20 mmol, 40%).

^{1}H-NMR (300 MHz, $CDCl_3$): δ = 7.45–7.22 (m, 5H), 2.10–1.95 (m, 2H), 1.92–1.63 (m, 7H), 1.39–1.23 (m, 1H). **^{19}F{^{1}H}-NMR** (282 MHz, $CDCl_3$) δ = –158.9 (broad). Calibrated ^{19}F{^{1}H}-NMR yield from benzylic fluoride: 70%. **^{13}C-NMR** (75 MHz, $CDCl_3$) δ = 146.1 (d, $^2J_{C–F}$ = 21.9 Hz, C_q), 128.3 (d, $^4J_{C–F}$ = 1.3 Hz, CH), 127.3 (d, $^5J_{C–F}$ = 1.5 Hz, CH), 124.1 (d, $^3J_{C–F}$ = 9.2 Hz, CH), 96.2 (d, $^1J_{C–F}$ = 173.7 Hz, C_q), 37.3 (d, $^2J_{C–F}$ = 23.8 Hz, CH_2), 25.1 (CH_2), 22.1 (d, $^3J_{C–F}$ = 1.7 Hz, CH_2). **IR** (ATR): $\tilde{\nu}$ = 2937, 2963, 1448, 1018, 949, 845, 758, 698, 542. **HR-MS** (EI) *m/z* calc. for $C_{12}H_{15}F$ $[M]^+$: 178.1152, found: 178.1153.

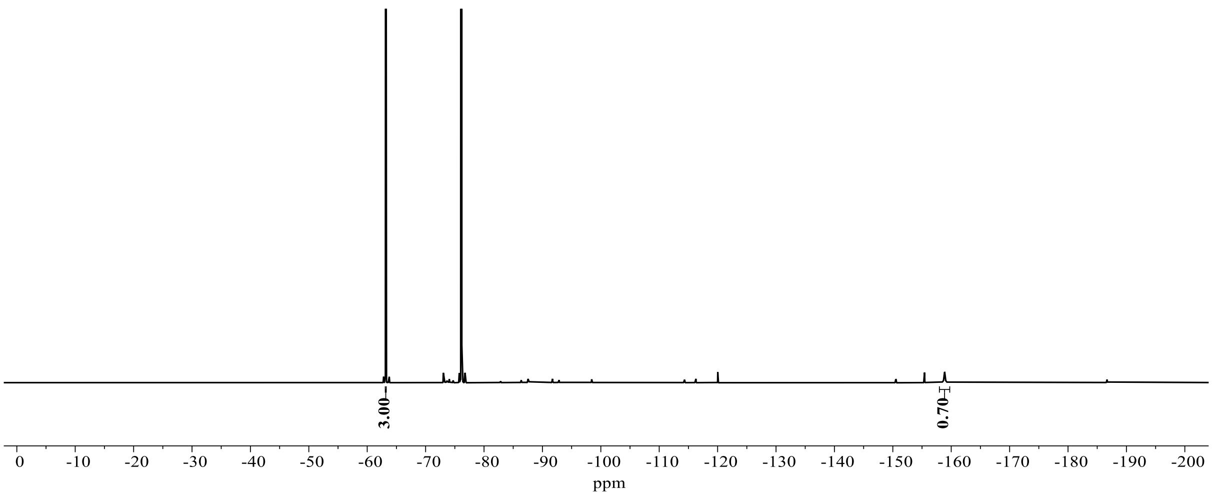

Figure 30 ^{19}F-NMR Spectrum (282 MHz, $CDCl_3$) of the reaction mixture with $PhCF_3$ (62 µL, 0.50 mmol) as internal standard (–63.2 ppm). The signals of the benzylic fluoride and internal standard are integrated.

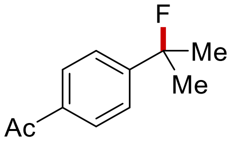

2g

4-(2-fluoropropan-2-yl)acetophenone

The general procedure A-2 was followed using 4-isopropylacetophenone **1g** (81.3 mg, 0.50 mmol) at −20 °C with 5.0 F/mol total charge.

2-Fluoropropan-2-yl C–H Shift: ^{1}H-NMR (300 MHz, CD_2Cl_2) δ = 1.72 (d, J = 21.9 Hz). Calibrated ^{1}H-NMR yield from 2-fluoropropan-2-yl proton: 42%. **Benzylic Fluoride Shift:** ^{19}F{^{1}H}-NMR (282 MHz, CD_2Cl_2) δ = −139.0. Calibrated ^{19}F{^{1}H}-NMR yield from benzylic fluoride: 42%. **HR-MS** (EI) *m/z* calc. for $C_{11}H_{13}FO$ $[M]^+$: 180.0950, found: 180.0945.

The spectral data are in accordance with those reported in literature.[4]

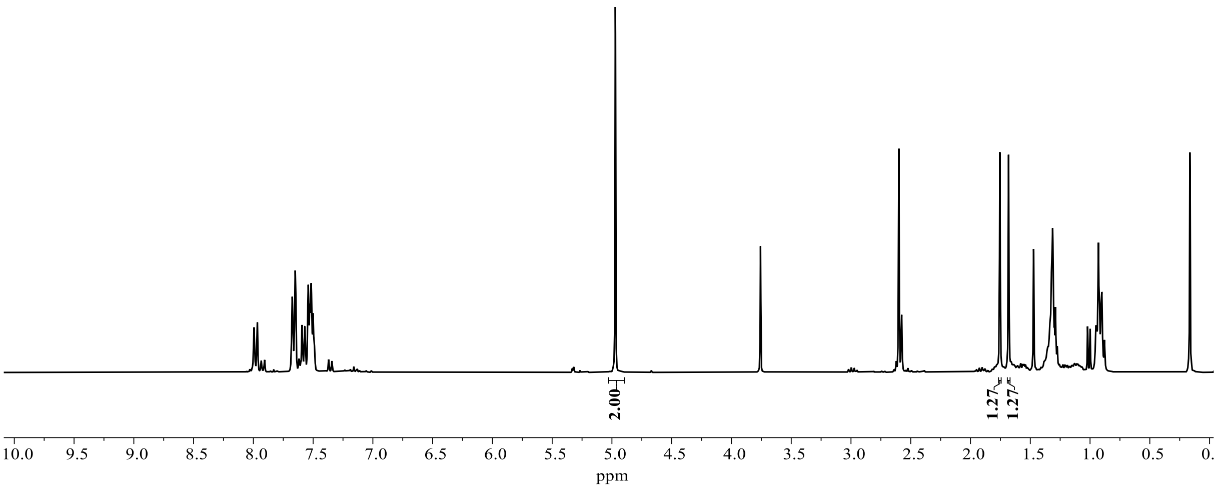

Figure 31 Crude ^{1}H-NMR Spectrum (300 MHz, CD_2Cl_2) of the reaction mixture with CH_2Br_2 (36 μL, 0.50 mmol) as internal standard (4.96 ppm). The signals of the fluoroisopropyl group and internal standard are integrated.

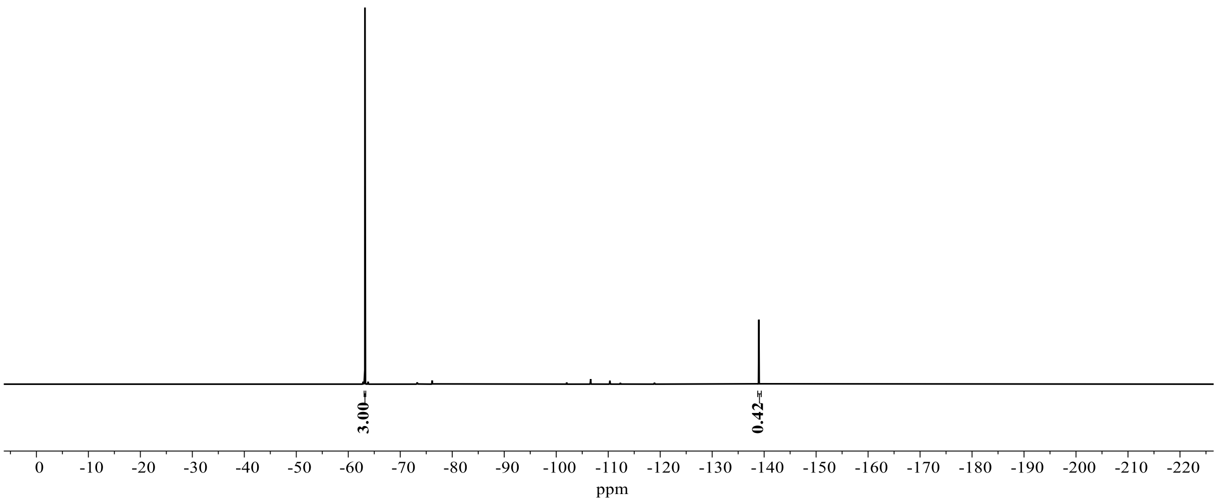

Figure 32 Crude ^{19}F{^{1}H}-NMR Spectrum (282 MHz, CD_2Cl_2) of the reaction mixture with $PhCF_3$ (62 μL, 0.50 mmol) as internal standard (–63.2 ppm). The signals of the benzylic fluoride and internal standard are integrated.

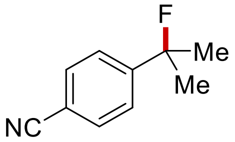

2h

4-(2-fluoropropan-2-yl)benzonitrile

The general procedure A-2 was followed using 4-isopropylbenzonitrile **1h** (72.7 mg, 0.50 mmol) at −20 °C with 5.0 F/mol total charge.

2-fluoropropan-2-yl C–H Shift: ^{1}H-NMR (300 MHz, CD_2Cl_2) δ = 1.68 (d, J = 22.0 Hz). Calibrated ^{1}H-NMR yield from 2-fluoropropan-2-yl proton: 46%. **Benzylic Fluoride Shift:**

$^{19}F\{^{1}H\}$-NMR (282 MHz, CD_2Cl_2) δ = −139.8. Calibrated $^{19}F\{^{1}H\}$-NMR yield from benzylic fluoride: 46%. **HR-MS** (EI) *m/z* calc. for $C_{10}H_{10}FN$ $[M]^+$: 163.0797, found: 163.0792.

The spectral data are in accordance with those reported in literature.[9]

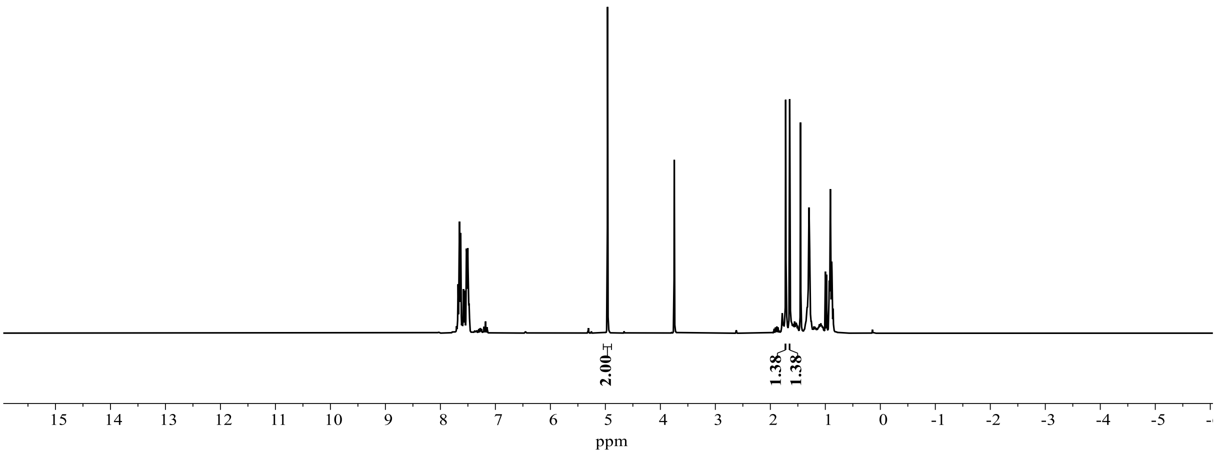

Figure 33 Crude ^{1}H-NMR Spectrum (300 MHz, CD_2Cl_2) of the reaction mixture with CH_2Br_2 (36 µL, 0.50 mmol) as internal standard (4.96 ppm). The signals of the fluoroisopropyl group and internal standard are integrated.

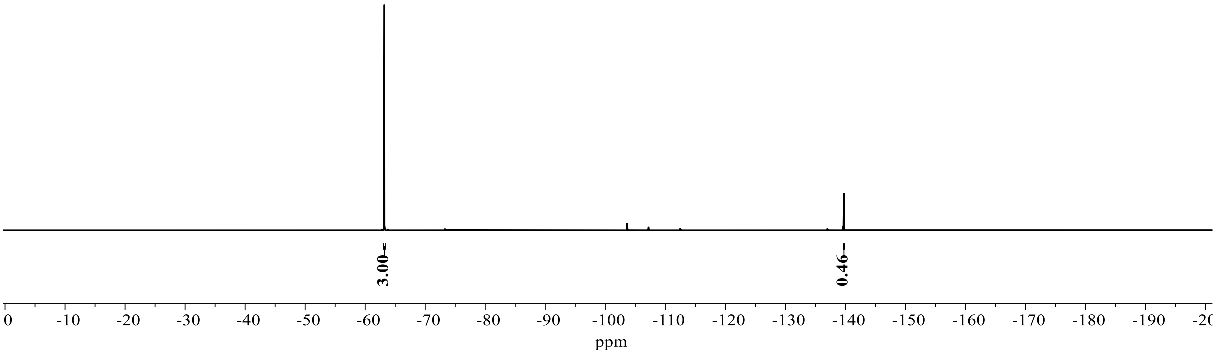

Figure 34 Crude $^{19}F\{^{1}H\}$-NMR Spectrum (282 MHz, CD_2Cl_2) of the reaction mixture with $PhCF_3$ (62 µL, 0.50 mmol) as internal standard (–63.2 ppm). The signals of the benzylic fluoride and internal standard are integrated.

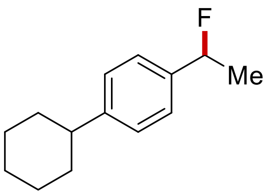

2i

1-Cyclohexyl-4-(1-fluoroethyl)benzene

The general procedure A-1 was followed using 1-cyclohexyl-4-ethylbenzene **1i** (94.3 mg, 0.50 mmol). After conducting the NMR analysis, the solvents were removed in vacuo, and the residue was purified by column chromatography on partially neutralized silica (*n*hexane) to obtain the product **2i** as a colorless oil (49.6 mg, 0.24 mmol, 48%).

^{1}H-NMR (400 MHz, $CDCl_3$): δ = 7.34–7.27 (m, 2H), 7.25–7.21 (m, 2H), 5.61 (dq, J = 47.8, 6.4 Hz, 1H), 2.60–2.44 (m, 1H), 1.94–1.81 (m, 4H), 1.80–1.73 (m, 1H), 1.65 (dd, J = 23.8, 6.5 Hz, 3H), 1.51–1.34 (m, 4H), 1.33–1.19 (m, 1H). Calibrated ^{1}H-NMR (300 MHz, C_6D_6) yield from benzylic proton: 55%. **^{19}F{^{1}H}-NMR** (282 MHz, $CDCl_3$) −164.6. Calibrated ^{19}F{^{1}H}-NMR (282 MHz, C_6D_6) yield from benzylic fluoride: 60%. **^{13}C-NMR** (101 MHz, $CDCl_3$): δ = 148.4 (d, $^5J_{C-F}$ = 2.3 Hz, C_q), 138.9 (d, $^2J_{C-F}$ = 19.5 Hz, CH), 127.1 (CH), 125.5 (d, $^3J_{C-F}$ =6.2, CH), 91.1 (d, $^1J_{C-F}$ = 166.3, CH), 44.5 (CH), 34.6 (CH_2), 27.0 (CH_2), 26.3 (CH_2), 22.8 (d, $^2J_{C-F}$ = 25.4, CH_3). **IR** (ATR): $\tilde{\nu}$ = 2982, 2924, 2852, 1449, 1068, 1006, 885, 828, 561. **HR-MS** (EI) *m/z* calc. for $C_{14}H_{19}F$ $[M]^+$: 206.1465, found: 206.1465.

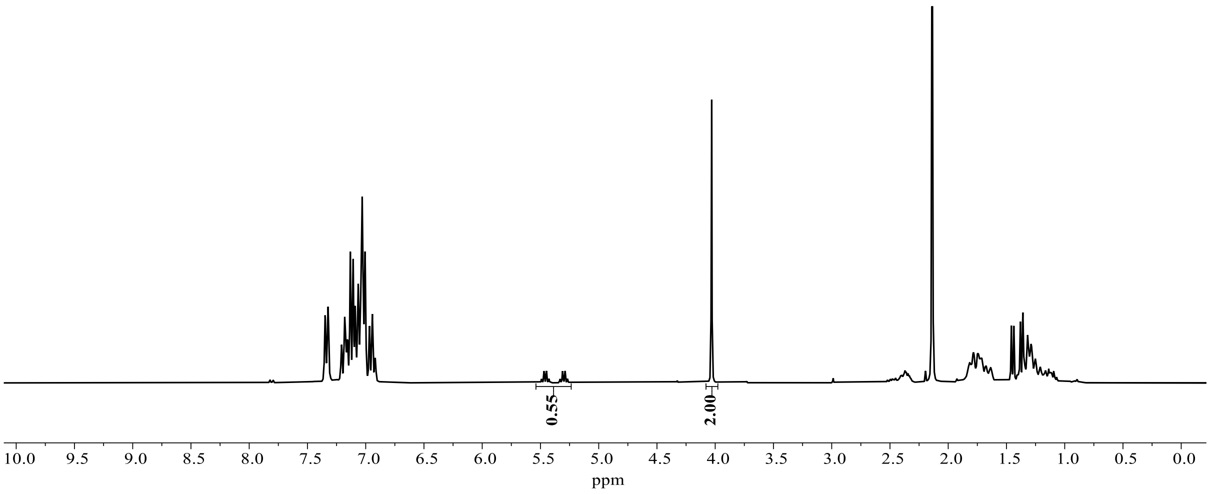

Figure 35 Crude ^{1}H-NMR Spectrum (300 MHz, C_6D_6) of the reaction mixture with CH_2Br_2 (36 µL, 0.50 mmol) as internal standard (4.03 ppm). The signals of the benzylic proton and internal standard are integrated.

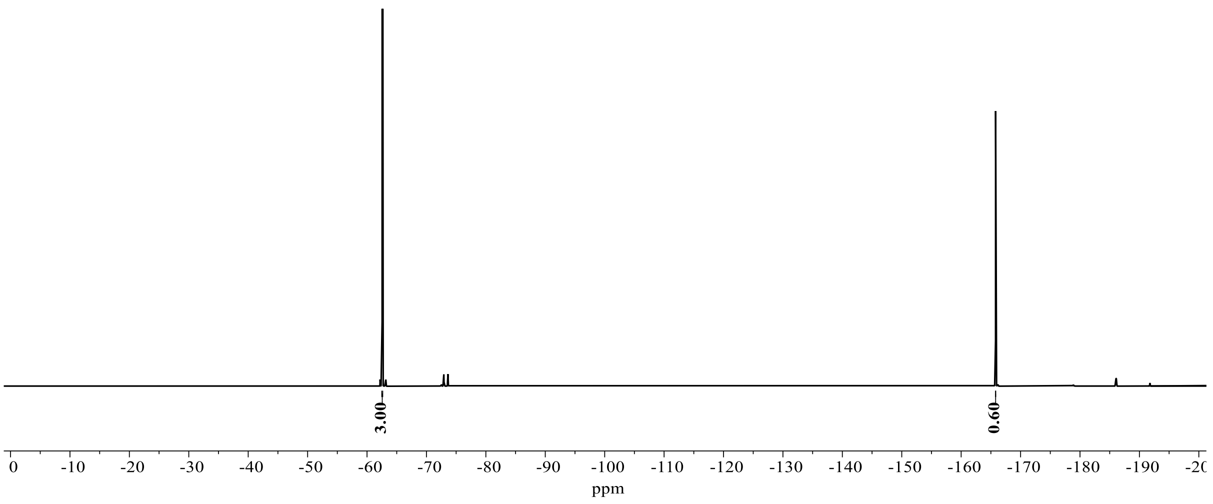

Figure 36 Crude ^{19}F-NMR Spectrum (282 MHz, C_6D_6) of the reaction mixture with $PhCF_3$ (62 µL, 0.50 mmol) as internal standard (–62.5 ppm). The signals of the benzylic fluoride and internal standard are integrated.

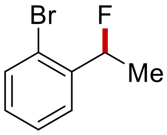

2j

1-Bromo-2-(1-fluoroethyl)benzene

The general procedure A-1 was followed using 1-bromo-2-ethylbenzene **1j** (92.5 mg, 0.50 mmol). The silica plug was flushed with EtOAc/nhexane/toluene (75 mL, 5:60:10).

Benzyl Fluoride C–H Shift: ^{1}H-NMR (300 MHz, $CDCl_3$): δ = 6.08 (dq, J = 46.4, 6.4 Hz). Calibrated ^{1}H-NMR yield from benzylic proton: 48%. **Benzylic Fluoride Shift:** ^{19}F{^{1}H}-NMR (282 MHz, $CDCl_3$): δ = –173.5. Calibrated ^{19}F{^{1}H}-NMR yield from benzylic fluoride: 45%. **HR-MS** (EI) *m/z* calc. for $C_8H_8F^{79}Br$ $[M]^+$: 201.9788, found: 201.9787.

The spectral data are in accordance with those reported in literature.[7]

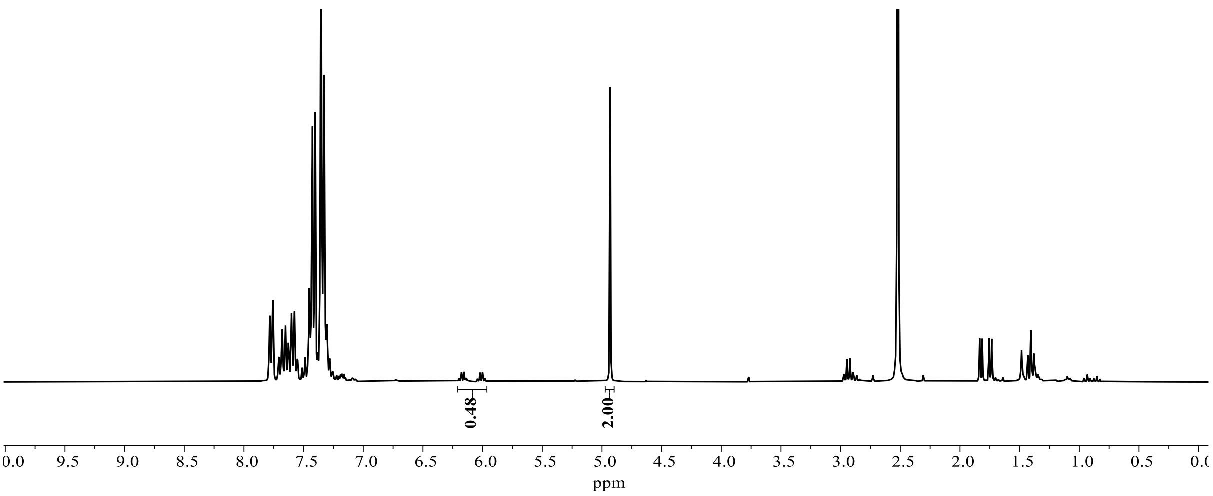

Figure 37 Crude ^{1}H-NMR Spectrum (300 MHz, $CDCl_3$) of the reaction mixture with CH_2Br_2 (36 μL, 0.50 mmol) as internal standard (4.93 ppm). The signals of the benzylic proton and internal standard are integrated.

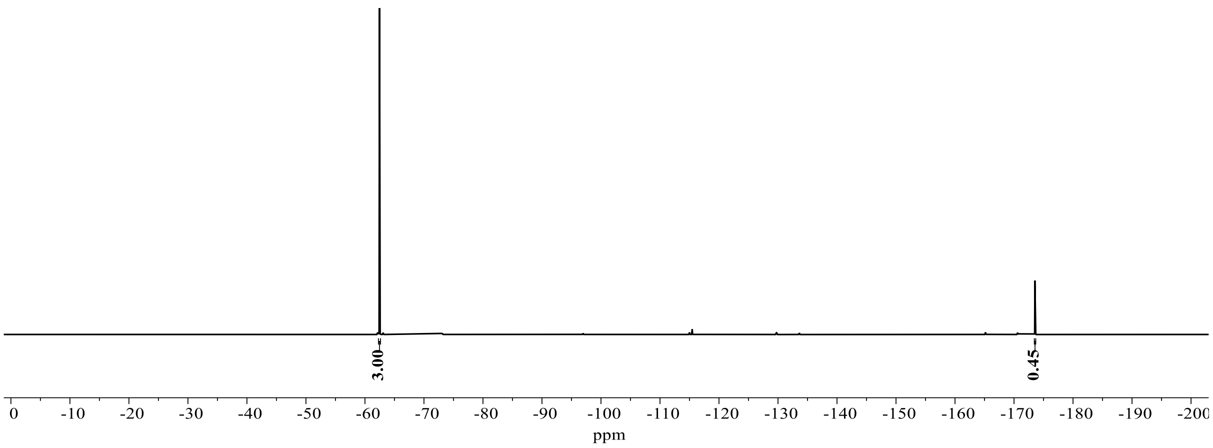

Figure 38 ^{19}F-NMR Spectrum (282 MHz, $CDCl_3$) of the reaction mixture with $PhCF_3$ (62 μL, 0.50 mmol) as internal standard (–62.5 ppm). The signals of the benzylic fluoride and internal standard are integrated.

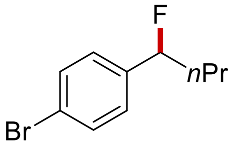

2k

1-Bromo-4-(1-fluorobutyl)benzene

The general procedure A-2 was followed using 1-bromo-4-butylbenzene **1k** (106.7 mg, 0.50 mmol) at 0 °C. After conducting the NMR analysis, the solvents were removed in vacuo, and the residue was purified by column chromatography (*n*hexane/EtOAc = 30:1) to obtain the product **2k** as a colorless oil (63.6 mg, 0.28 mmol, 55%).

1H-NMR (300 MHz, $CDCl_3$) δ = 7.50 (d, J = 7.8 Hz, 2H), 7.20 (d, J = 7.8 Hz, 2H), 5.39 (ddd, J = 47.7, 8.0, 4.9 Hz, 1H), 2.06–1.63 (m, 2H), 1.60–1.28 (m, 2H), 0.96 (t, J = 7.4 Hz, 3H).Calibrated ^{1}H-NMR (300 MHz, CD_2Cl_2) yield from benzylic proton: 87%. **^{19}F{^{1}H}-NMR** (282 MHz, $CDCl_3$): δ = −175.5. Calibrated ^{19}F{^{1}H}-NMR (282 MHz, CD_2Cl_2) yield from benzylic fluoride: 87%. **^{13}C-NMR** (75 MHz, $CDCl_3$) δ = 139.8 (d, $^{2}J_{C–F}$ = 20.3 Hz, C_q), 131.7 (CH), 127.4 (d, $^{3}J_{C–F}$ = 6.9 Hz), 122.2 (d, $^{5}J_{C–F}$ = 2.5 Hz, C_q), 93.9 (d, $^{1}J_{C–F}$ = 171.1 Hz, CH), 39.3 (d, $^{2}J_{C–F}$ = 23.2 Hz, CH_2), 18.4 (d, $^{3}J_{C–F}$ = 4.5 Hz, CH_2), 13.9 (CH_3). **IR** (ATR): $\tilde{\nu}$ = 2962, 2935, 2874, 1597, 1490, 1072, 1012, 954, 824, 543. **HR-MS** (EI) *m/z* calc. for $C_{10}H_{12}{}^{79}BrF$ $[M]^{+}$: 230.0106, found: 230.0101.

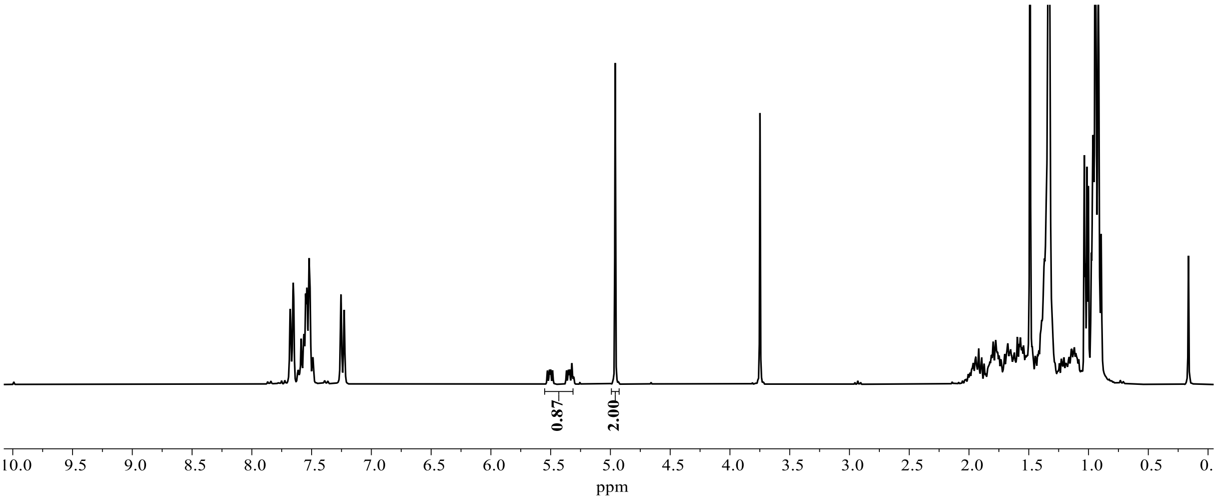

Figure 39 Crude ^{1}H-NMR Spectrum (300 MHz, CD_2Cl_2) of the reaction mixture with CH_2Br_2 (36 µL, 0.50 mmol) as internal standard (4.96 ppm). The signals of the benzylic proton and internal standard are integrated.

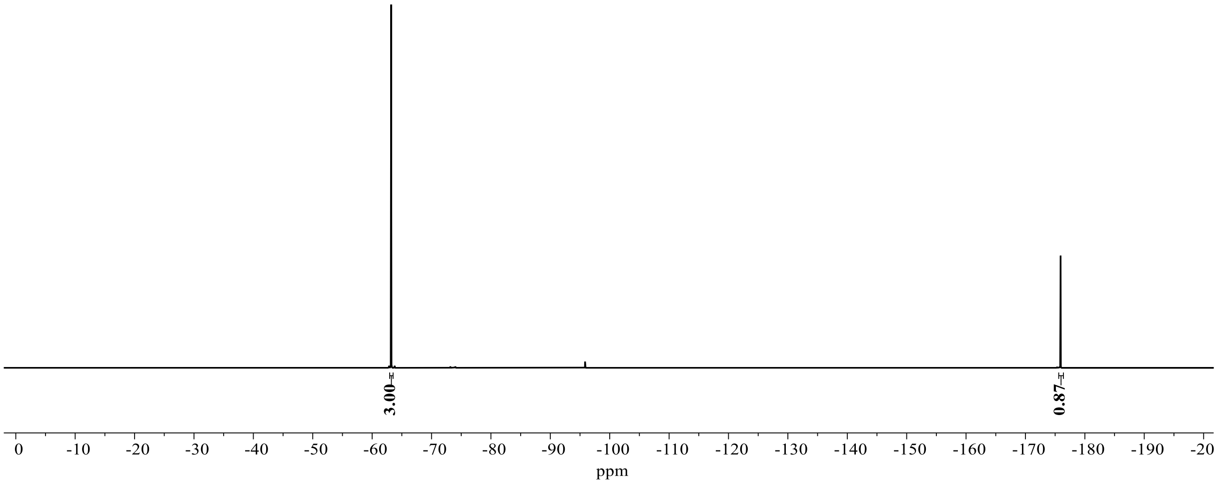

Figure 40 Crude ^{19}F{^{1}H}-NMR Spectrum (282 MHz, CD_2Cl_2) of the reaction mixture with $PhCF_3$ (62 µL, 0.50 mmol) as internal standard (–63.2 ppm). The signals of the benzylic fluoride and internal standard are integrated.

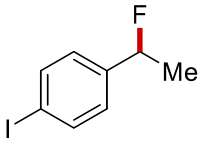

2l

1-(1-Fluoroethyl)-4-iodobenzene

The general procedure A-1 was followed using 1-ethyl-4-iodobenzene **1l** (116 mg, 0.50 mmol). After conducting the NMR analysis, the solvents were removed in vacuo, and the residue was purified by column chromatography (*n*hexane) to obtain the product **2l** as a colorless oil (85.6 mg, 0.34 mmol, 68%).

^{1}H-NMR (400 MHz, C_6D_6): δ = 7.44–7.37 (m, 2H), 6.66–6.60 (m, 2H), 5.08 (dq, *J* = 47.8, 6.5 Hz, 1H), 1.19 (dd, *J* = 23.4, 6.6 Hz, 3H). Calibrated ^{1}H-NMR yield from benzylic proton: 88%.**^{19}F{^{1}H}-NMR** (282 MHz, C_6D_6): δ = −169.1. Calibrated ^{19}F{^{1}H}-NMR yield from benzylic fluoride: 90%. **^{13}C-NMR** (101 MHz, C_6D_6): δ = 141.6 (d, $^2J_{C–F}$ = 20.0 Hz, C_q), 137.8 (CH), 127.2 (d, $^3J_{C–F}$ = 6.9 Hz, CH), 93.9 (d, $^5J_{C–F}$ = 2.5 Hz, C_q), 90.1 (d, $^1J_{C–F}$ = 169.7 Hz, CH), 22.8 (d, $^2J_{C–F}$ = 25.1 Hz. CH_3). **IR** (ATR): $\tilde{\nu}$ = 1591, 1484, 1400, 1335, 1064, 1003, 879, 818, 768, 529. **HR-MS** (EI) *m/z* calc. for C_8H_8FI $[M]^+$: 249.9649, found: 249.9651.

The spectral data are in accordance with those reported in literature.[5]

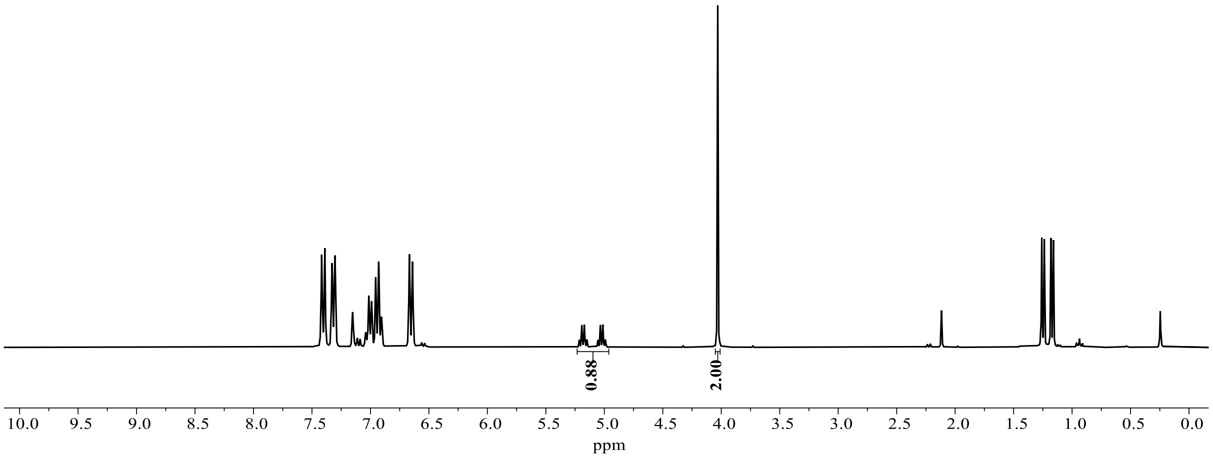

Figure 41 Crude ^{1}H-NMR Spectrum (300 MHz, C_6D_6) of the reaction mixture with CH_2Br_2 (36 µL, 0.50 mmol) as internal standard (4.03 ppm). The signals of the benzylic proton and internal standard are integrated.

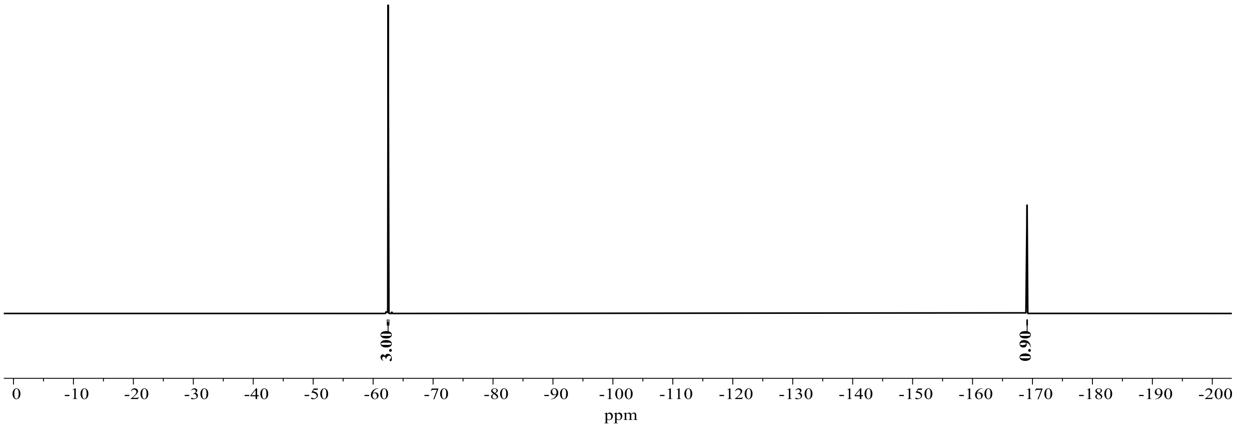

Figure 42 Crude ^{19}F-NMR Spectrum (282 MHz, C_6D_6) of the reaction mixture with $PhCF_3$ (62 µL, 0.50 mmol) as internal standard (–62.5 ppm). The signals of the benzylic fluoride and internal standard are integrated.

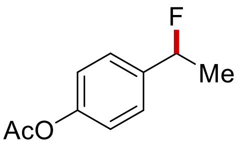

2m

4-(1-Fluoroethyl)phenyl acetate

The general procedure A was followed using 4-ethylphenyl acetate **1m** (82.1 mg, 0.50 mmol) with 3.0 F/mol total charge at –15 °C.

Benzyl Fluoride C–H Shift: ^{1}H-NMR (300 MHz, $CDCl_3$): δ = 5.64 (dq, J = 47.8, 6.4 Hz). Calibrated ^{1}H-NMR yield from benzylic proton: 64%. **Benzylic Fluoride Shift:** ^{19}F{^{1}H}-NMR (282 MHz, $CDCl_3$): δ = –166.4. Calibrated ^{19}F{^{1}H}-NMR yield from benzylic fluoride: 70%. **HR-MS** (EI) *m/z* calc. for $C_{10}H_{11}FO_2$ $[M]^+$: 182.0738, found: 182.0739.

The spectral data are in accordance with those reported in literature.[10]

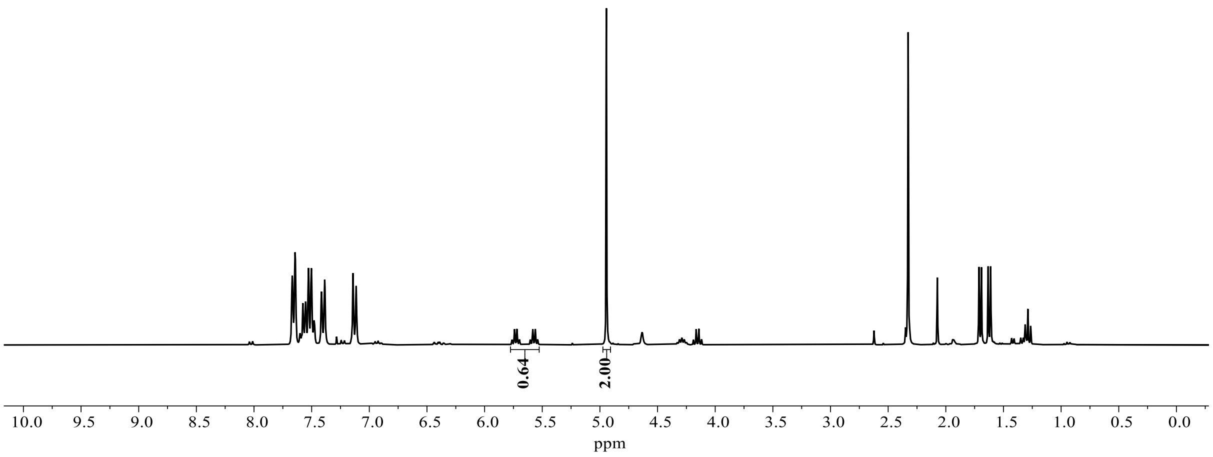

Figure 43 Crude ^{1}H-NMR Spectrum (300 MHz, $CDCl_3$) of the reaction mixture with CH_2Br_2 (36 µL, 0.50 mmol) as internal standard (4.93 ppm). The signals of the benzylic proton and internal standard are integrated.

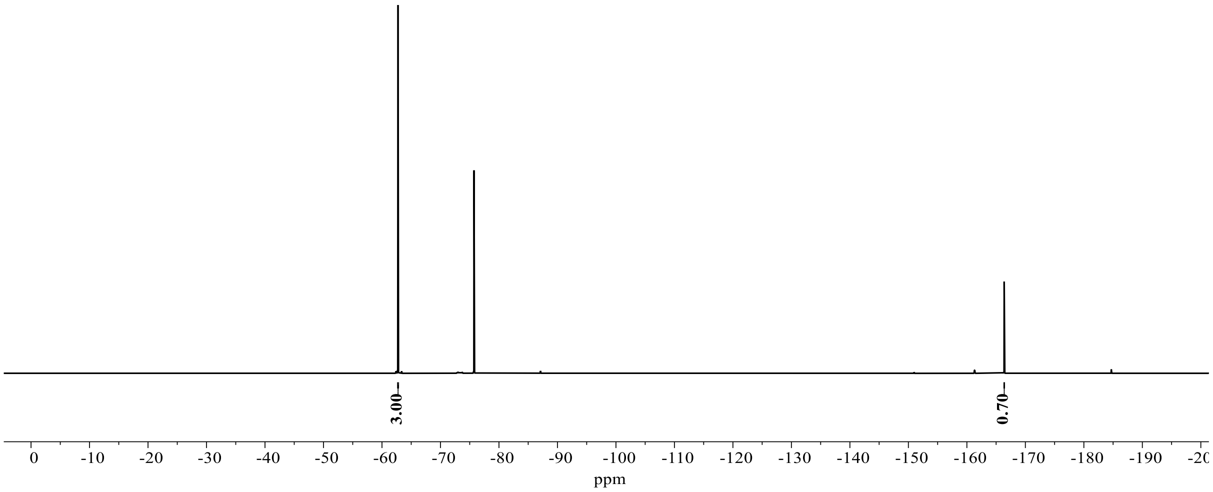

Figure 44 ^{19}F-NMR Spectrum (282 MHz, $CDCl_3$) of the reaction mixture with $PhCF_3$ (62 µL, 0.50 mmol) as internal standard (–62.7 ppm). The signals of the benzylic fluoride and internal standard are integrated.

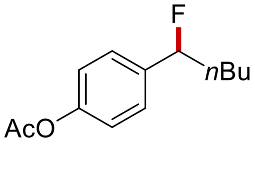

2n

4-(1-fluoropentyl)phenyl acetate

The general procedure A-2 was followed using 4-pentylphenyl acetate **1n** (103 mg, 0.50 mmol) at −20 °C. After conducting the NMR analysis, the solvents were removed in vacuo, and the residue was purified by column chromatography on partially neutralized silica (*n*hexane/EtOAc = 30:1) to obtain the product **2n** as a colorless oil (57.0 mg, 0.25 mmol, 50%).

1H-NMR (300 MHz, $CDCl_3$) δ = 7.34 (d, J = 8.4 Hz, 2H), 7.10 (d, J = 8.4 Hz, 2H), 5.41 (ddd, J = 47.7, 8.1, 4.9 Hz, 1H), 2.30 (s, 3H), 2.08–1.66 (m, 2H), 1.56–1.23 (m, 2H), 0.91 (t, J =

6.9 Hz, 3H). Calibrated ^{1}H-NMR yield from benzylic proton: 62%. **^{19}F{^{1}H}-NMR** (282 MHz, $CDCl_3$): δ = −173.6. Calibrated ^{19}F{^{1}H}-NMR yield from benzylic fluoride: 66%. **^{13}C-NMR** (101 MHz, $CDCl_3$) δ = 169.5 (C_q), 150.5 (d, $^5J_{C-F}$ = 2.2 Hz, C_q), 138.3 (d, $^2J_{C-F}$ = 20.3 Hz, C_q), 126.8 (d, $^3J_{C-F}$ = 6.8 Hz, CH), 121.7 (CH), 94.3 (d, $^1J_{C-F}$ = 170.5 Hz, CH), 37.0 (d, $^2J_{C-F}$ = 23.5 Hz, CH), 27.3 (d, $^3J_{C-F}$ = 4.2 Hz, CH_2), 22.6 (CH_2), 21.2 (CH_3), 14.0 (CH_3). **IR** (ATR): $\tilde{\nu}$ = 2958, 2936, 2867, 1760, 1510, 1369, 1190, 912, 851, 550. **HR-MS** (EI) *m/z* calc. for $C_{13}H_{17}FO_2$ $[M]^+$: 224.1213, found: 224.1207.

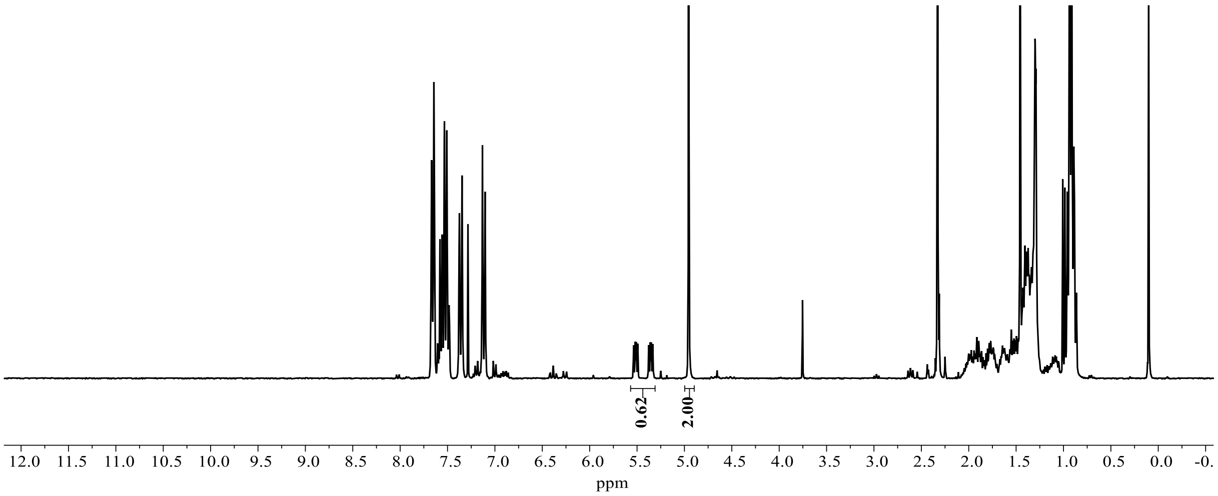

Figure 45 Crude ^{1}H-NMR Spectrum (300 MHz, $CDCl_3$) of the reaction mixture with CH_2Br_2 (36 µL, 0.50 mmol) as internal standard (4.93 ppm). The signals of the benzylic proton and internal standard are integrated.

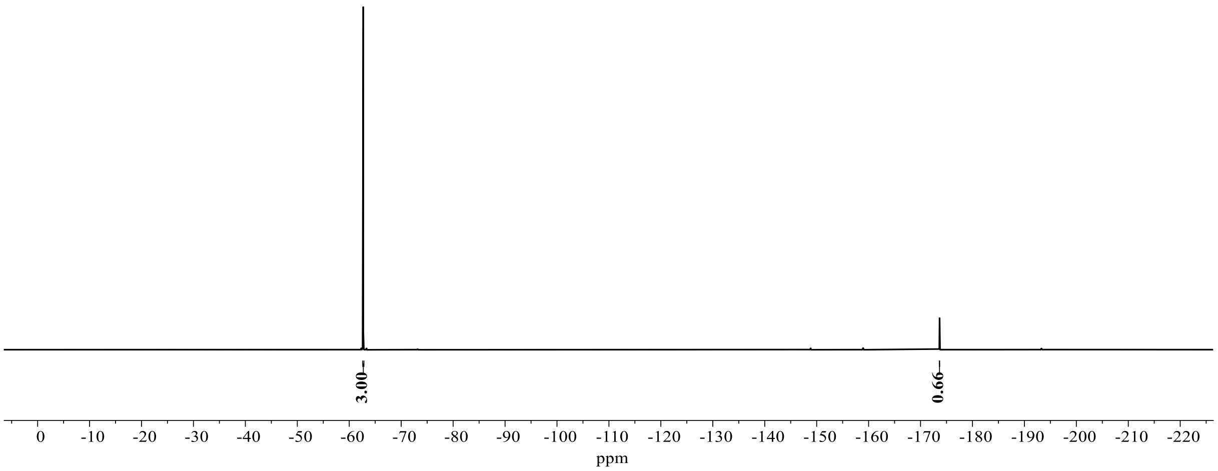

Figure 46 ^{19}F-NMR Spectrum (282 MHz, $CDCl_3$) of the reaction mixture with $PhCF_3$ (62 µL, 0.50 mmol) as internal standard (–62.7 ppm). The signals of the benzylic fluoride and internal standard are integrated.

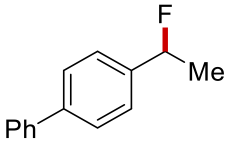

2o

4-(1-fluoroethyl)-1,1'-biphenyl

The general procedure A-1 was followed using 4-ethyl-1,1'-biphenyl **1o** (91.2 mg, 0.50 mmol). **Benzyl Fluoride C–H Shift:** ^{1}H-NMR (300 MHz, d_6-Acetone) δ = 5.72 (dq, J = 48.2, 6.4 Hz). Calibrated ^{1}H-NMR yield from the benzylic proton: 79%. **Benzylic Fluoride Shift:** ^{19}F{^{1}H}-NMR (282 MHz, d_6-Acetone) δ = –161.9. Calibrated ^{19}F{^{1}H}-NMR yield from benzylic fluoride: 81%. **HR-MS** (EI) *m/z* calc. for $C_{14}H_{13}F$ [M]$^{+}$: 200.0996, found: 200.0996.

The spectral data are in accordance with those reported in literature.[4]

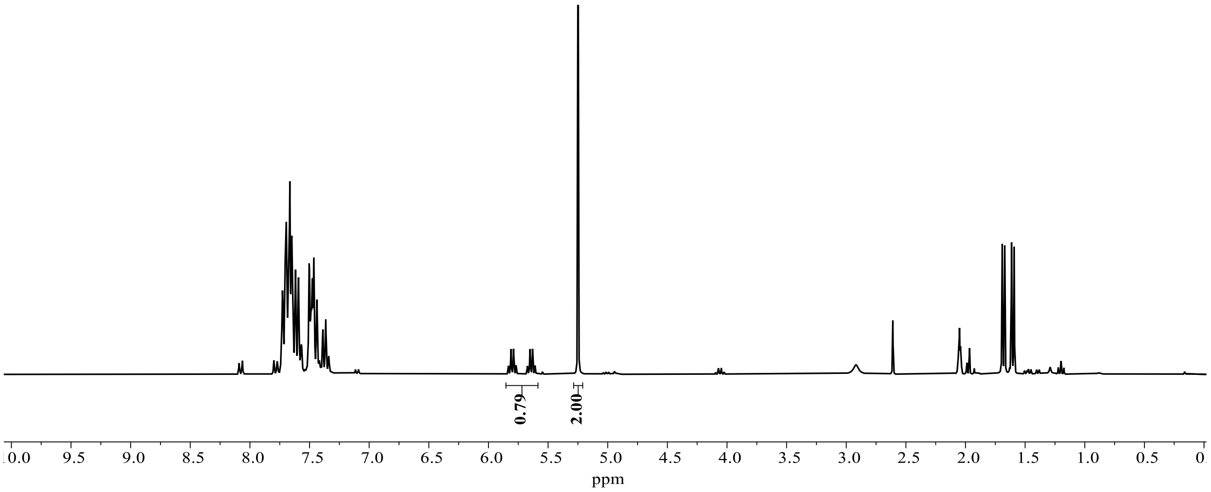

Figure 47 Crude ^{1}H-NMR Spectrum (300 MHz, d_6-Acetone) of the reaction mixture with CH_2Br_2 (36 µL, 0.50 mmol) as internal standard (5.25 ppm). The signals of the benzylic proton and internal standard are integrated.

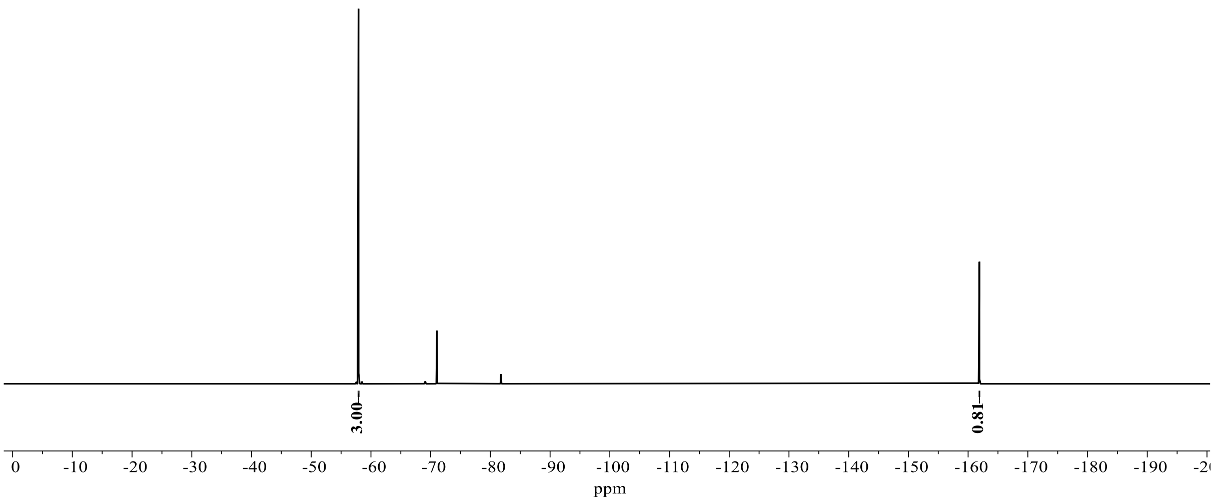

Figure 48 Crude ^{19}F-NMR Spectrum (282 MHz, d_6-Acetone) of the reaction mixture with $PhCF_3$ (62 µL, 0.50 mmol) as internal standard (–57.9 ppm). The signals of the benzylic fluoride and internal standard are integrated.

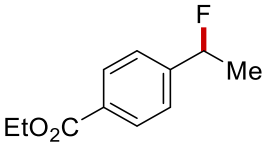

2p

Ethyl 4-(1-fluoroethyl)benzoate

The general procedure A-2 was followed using ethyl 4-ethylbenzoate **1p** (89.3 mg, 0.50 mmol) at −20 °C with 4.0 F/mol total charge.

Benzyl Fluoride C–H Shift: ^{1}H-NMR (300 MHz, C_6D_6) δ = 5.19 (dq, J = 47.6, 6.4 Hz). Calibrated ^{1}H-NMR yield from benzylic proton: 44%. **Benzylic Fluoride Shift:** ^{19}F{^{1}H}-NMR (282 MHz, C_6D_6) δ = −171.2. Calibrated ^{19}F{^{1}H}-NMR yield from benzylic fluoride: 46%. HR-MS (EI) *m/z* calc. for $C_{11}H_{13}FO_2$ $[M]^+$: 196.0900, found: 196.0894.

The spectral data are in accordance with those reported in literature.[8]

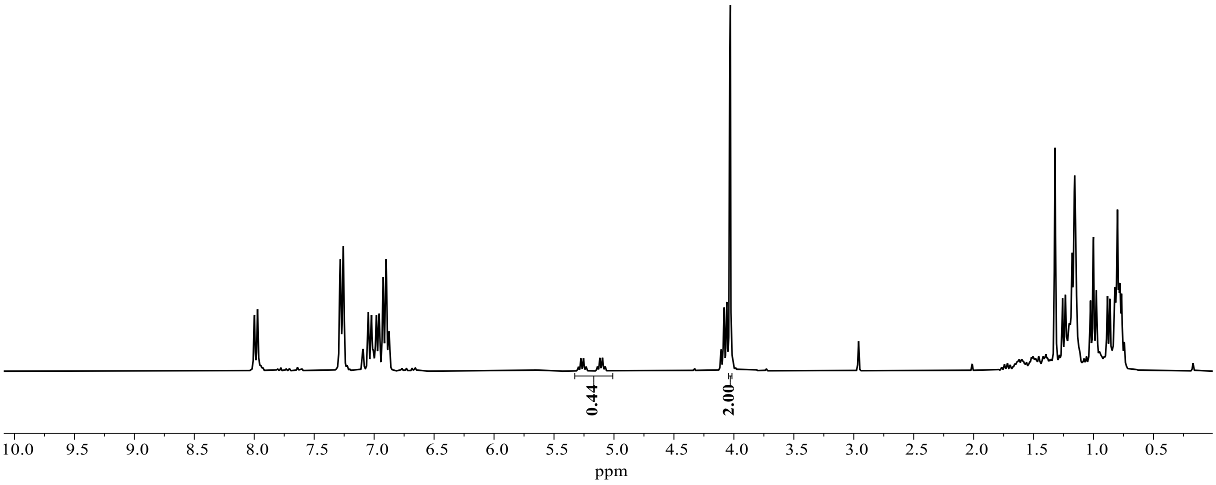

Figure 49 Crude ^{1}H-NMR Spectrum (300 MHz, C_6D_6) of the reaction mixture with CH_2Br_2 (36 μL, 0.50 mmol) as internal standard (4.03 ppm). The signals of the benzylic proton and internal standard are integrated.

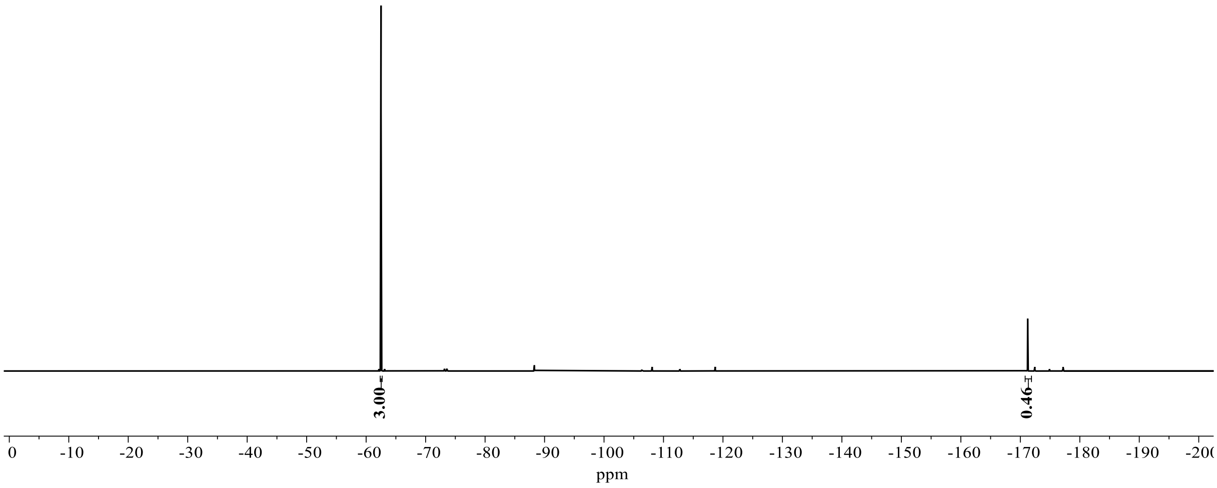

Figure 50 Crude ^{19}F{^{1}H}-NMR Spectrum (282 MHz, C_6D_6) of the reaction mixture with $PhCF_3$ (62 μL, 0.50 mmol) as internal standard (–63.5 ppm). The signals of the benzylic fluoride and internal standard are integrated.

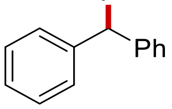

2q

(Fluoromethylene)dibenzene

The general procedure A-1 was followed using diphenylmethane **1q** (84.1 mg, 0.50 mmol).

Benzyl Fluoride C–H Shift: ^{1}H-NMR (300 MHz, $CDCl_3$): δ = 6.52 (d, J = 47.7 Hz). Calibrated ^{1}H-NMR yield from benzylic proton: 64%. **Benzylic Fluoride Shift:** ^{19}F{^{1}H}-NMR (282 MHz, $CDCl_3$): δ = –166.7. Calibrated ^{19}F{^{1}H}-NMR yield from benzylic fluoride: 65%. **HR-MS** (EI) *m/z* calc. for $C_{13}H_{11}F$ $[M]^+$: 186.0839, found: 186.0840.

The spectral data are in accordance with those reported in literature.[8]

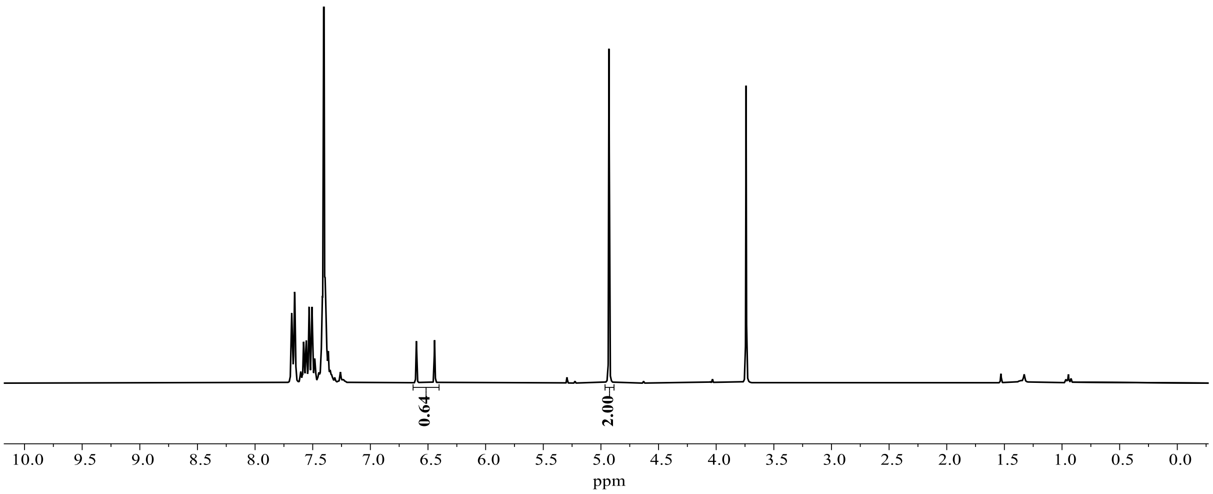

Figure 51 Crude ^{1}H-NMR Spectrum (300 MHz, $CDCl_3$) of the reaction mixture with CH_2Br_2 (36 µL, 0.50 mmol) as internal standard (4.93 ppm). The signals of the benzylic proton and internal standard are integrated.

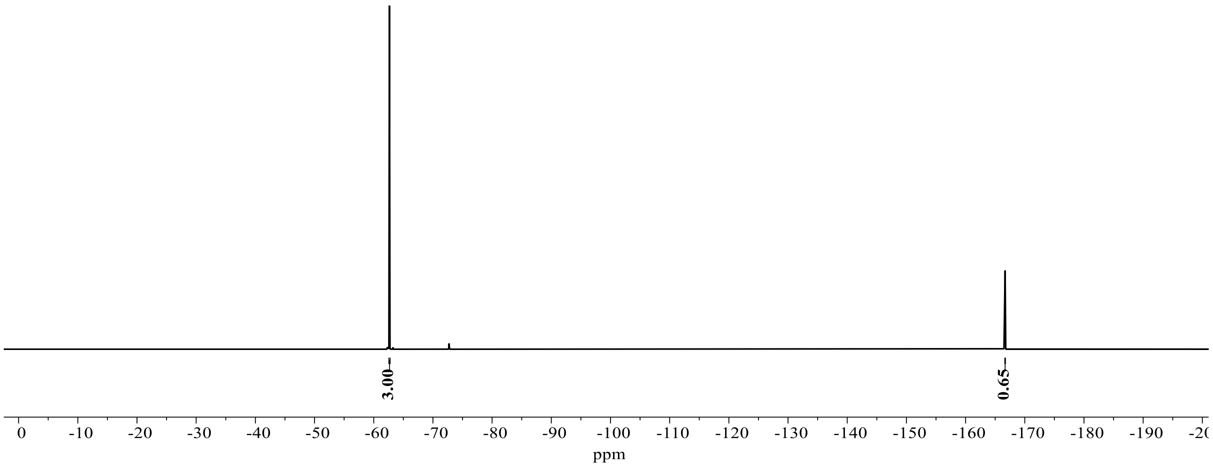

Figure 52 ^{19}F-NMR Spectrum (282 MHz, $CDCl_3$) of the reaction mixture with $PhCF_3$ (62 µL, 0.50 mmol) as internal standard (–62.6 ppm). The signals of the benzylic fluoride and internal standard are integrated.

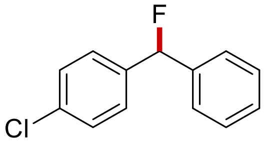

2r

1-Chloro-4-(fluoro(phenyl)methyl)benzene

The general procedure A-2 was followed using 4-chlorodiphenylmethane **1r** (81.3 mg, 0.50 mmol) at −20 °C with 3.0 F/mol total charge.

Benzyl Fluoride C–H Shift: ^{1}H-NMR (300 MHz, CD_2Cl_2) δ = 6.47 (d, J = 47.3 Hz). Calibrated ^{1}H-NMR yield from benzylic proton: 74%. **Benzylic Fluoride Shift:** ^{19}F{^{1}H}-NMR (282 MHz,

CD_2Cl_2) δ = −167.4. Calibrated $^{19}F\{^1H\}$-NMR yield from benzylic fluoride: 74%. **HR-MS** (EI) *m/z* calc. for $C_{13}H_{10}{}^{35}ClF$ $[M]^+$: 220.0455, found: 220.0450.

The spectral data are in accordance with those reported in literature.[11]

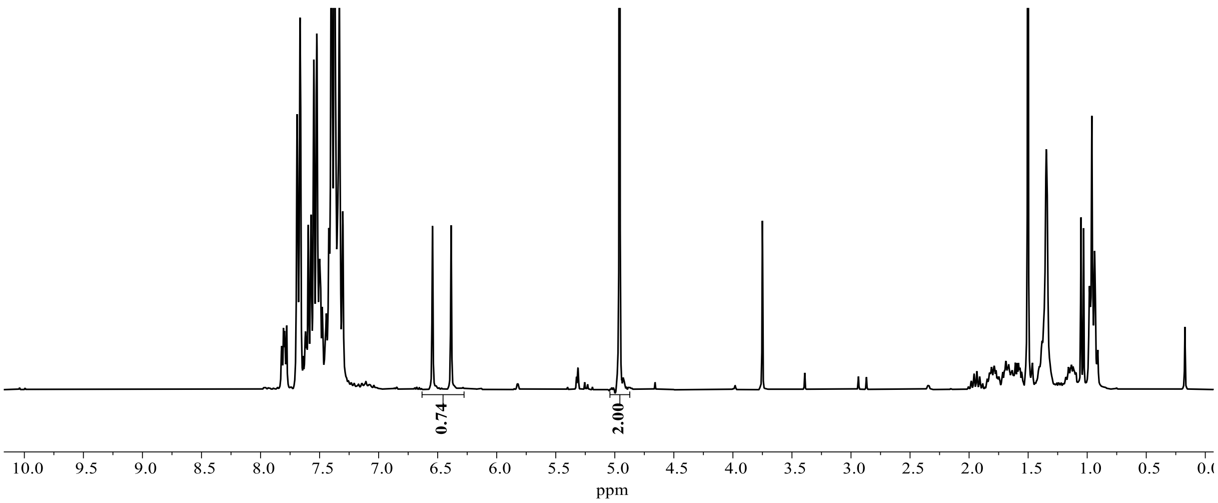

Figure 53 Crude ^1H-NMR Spectrum (300 MHz, CD_2Cl_2) of the reaction mixture with CH_2Br_2 (36 µL, 0.50 mmol) as internal standard (4.96 ppm). The signals of the benzylic proton and internal standard are integrated.

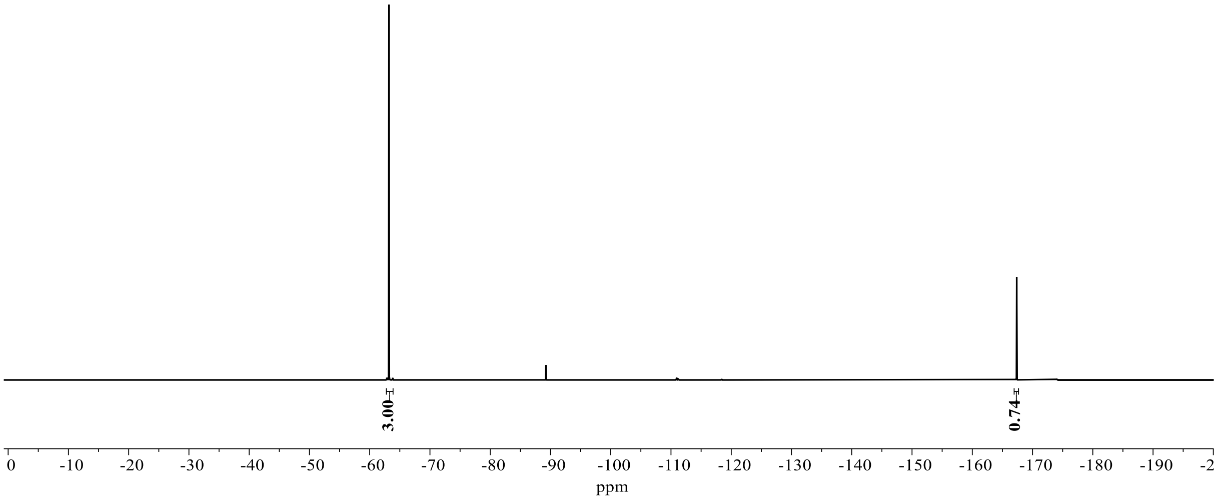

Figure 54 Crude $^{19}F\{^1H\}$-NMR Spectrum (282 MHz, CD_2Cl_2) of the reaction mixture with $PhCF_3$ (62 µL, 0.50 mmol) as internal standard (–63.2 ppm). The signals of the benzylic fluoride and internal standard are integrated.

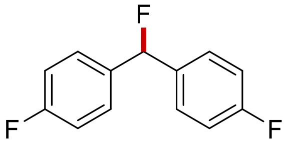

2s

4,4'-(fluoromethylene)bis(fluorobenzene)

The general procedure A-2 was followed using 4,4’-difluorodiphenylmethane **1s** (102 mg, 0.50 mmol) at −20 °C with 3.0 F/mol total charge.

Benzyl Fluoride C–H Shift: ^{1}H-NMR (300 MHz, CD_2Cl_2) δ = 6.45 (d, J = 47.4 Hz). Calibrated ^{1}H-NMR yield from benzylic proton: 70%. **Benzylic Fluoride Shift:** ^{19}F{^{1}H}-NMR (282 MHz, CD_2Cl_2) δ = −164.0 (t, J = 3.6 Hz). Calibrated ^{19}F{^{1}H}-NMR yield from benzylic fluoride: 70%. **HR-MS** (EI) *m/z* calc. for $C_{13}H_9F_3$ $[M]^+$: 222.0656, found: 222.0651.

The spectral data are in accordance with those reported in literature.[8]

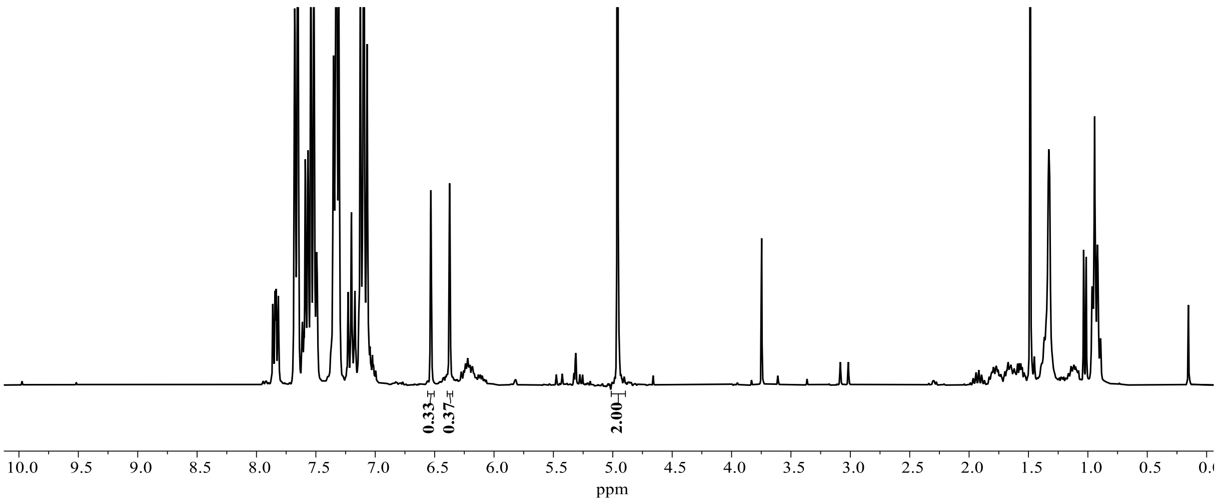

Figure 55 Crude ^{1}H-NMR Spectrum (300 MHz, CD_2Cl_2) of the reaction mixture with CH_2Br_2 (36 µL, 0.50 mmol) as internal standard (4.96 ppm). The signals of the benzylic proton and internal standard are integrated.

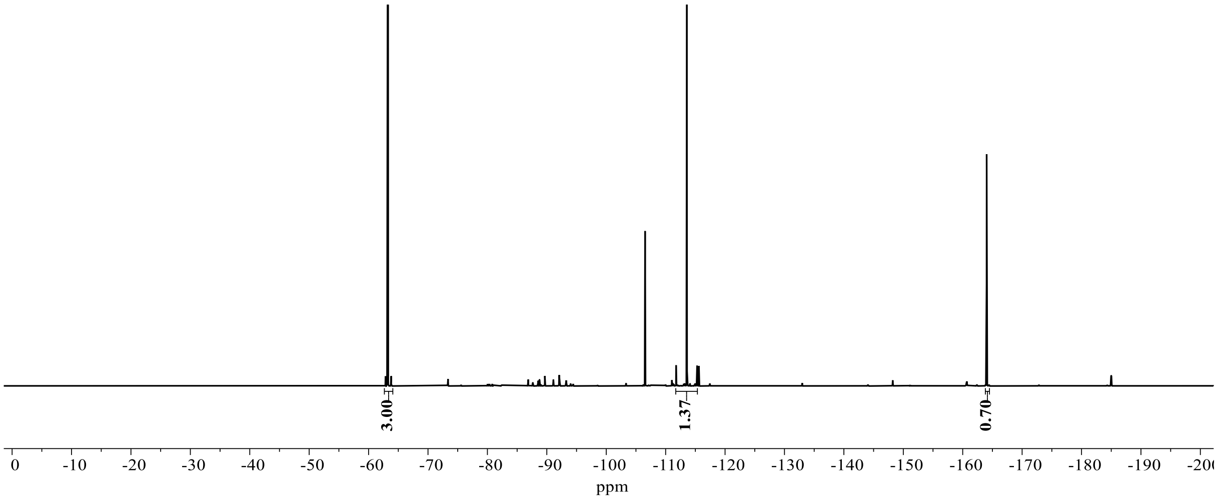

Figure 56 Crude $^{19}F\{^1H\}$-NMR Spectrum (282 MHz, CD_2Cl_2) of the reaction mixture with $PhCF_3$ (62 µL, 0.50 mmol) as internal standard (–63.2 ppm). The signals of the benzylic fluoride, aromatic fluoride and internal standard are integrated.

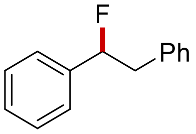

2t

(1-Fluoroethane-1,2-diyl)dibenzene

The general procedure A-1 was followed using dibenzyl **1t** (91.1 mg, 0.50 mmol) with a total charge of 5.0 F/mol using constant current electrolysis at 16 mA at 0 °C.

Benzyl Fluoride C–H Shift: ^1H-NMR (300 MHz, $CDCl_3$) δ = 5.69 (ddd, J = 47.4, 4.7, 4.7 Hz). Calibrated ^1H-NMR yield from benzylic proton: 56%. **Benzylic Fluoride Shift:** $^{19}F\{^1H\}$-NMR (282 MHz, $CDCl_3$) δ = –173.0. Calibrated $^{19}F\{^1H\}$-NMR yield from benzylic fluoride: 51%. **HR-MS** (EI) *m/z* calc. for $C_{14}H_{13}F$ $[M]^+$: 200.0996, found: 200.0995.

The spectral data are in accordance with those reported in literature.[12]

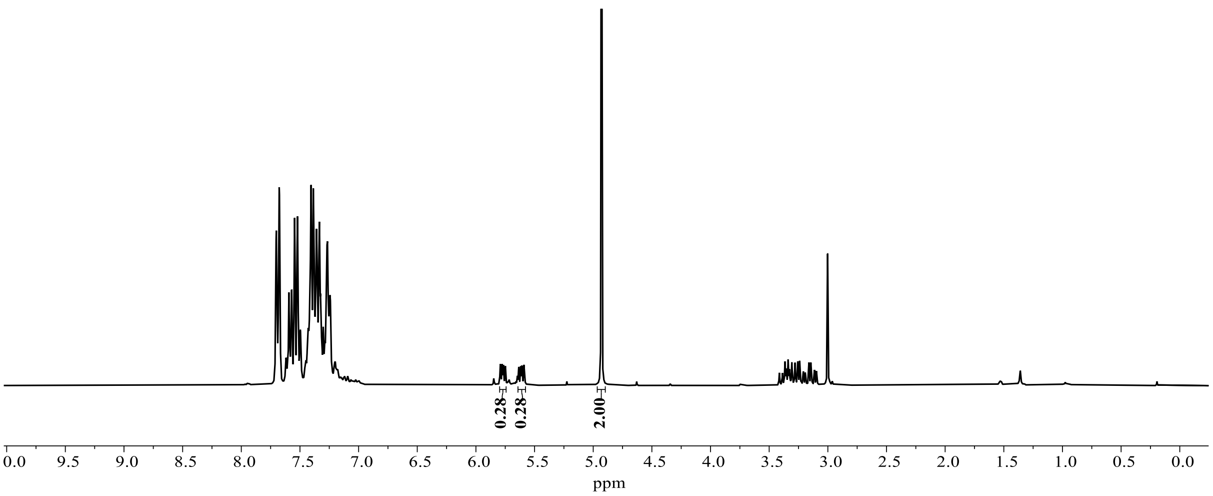

Figure 57 Crude ^{1}H-NMR Spectrum (300 MHz, $CDCl_3$) of the reaction mixture with CH_2Br_2 (36 µL, 0.50 mmol) as internal standard (4.93 ppm). The signals of the benzylic proton and internal standard are integrated.

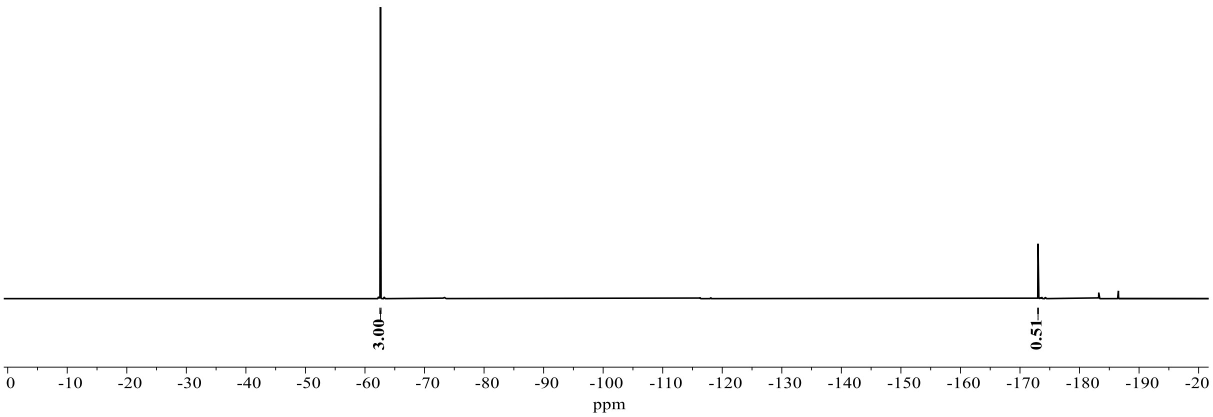

Figure 58 ^{19}F-NMR Spectrum (282 MHz, $CDCl_3$) of the reaction mixture with $PhCF_3$ (62 µL, 0.50 mmol) as internal standard (–62.6 ppm). The signals of the benzylic fluoride and internal standard are integrated.

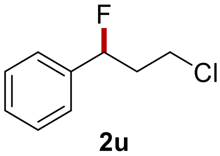

2u

(3-Chloro-1-fluoropropyl)benzene

The general procedure A-1 was followed using (3-chloro-1-propyl)benzene **1u** (77.3 mg, 0.50 mmol) with 3.0 F/mol total charge.

Benzyl Fluoride C–H Shift: ^{1}H-NMR (300 MHz, C_6D_6) δ = 5.42 (ddd, J = 48.3, 9.1, 4.0 Hz, 1H). Calibrated ^{1}H-NMR yield from benzylic proton: 75%. **Benzylic Fluoride Shift:** $^{19}F\{^{1}H\}$-NMR (282 MHz, C_6D_6) δ = –179.5. Calibrated $^{19}F\{^{1}H\}$-NMR yield from benzylic fluoride: 75%. **HR-MS** (EI) *m/z* calc. for $C_9H_{10}ClF$ $[M]^+$: 172.0450, found: 172.0448.

The spectral data are in accordance with those reported in literature.[13]

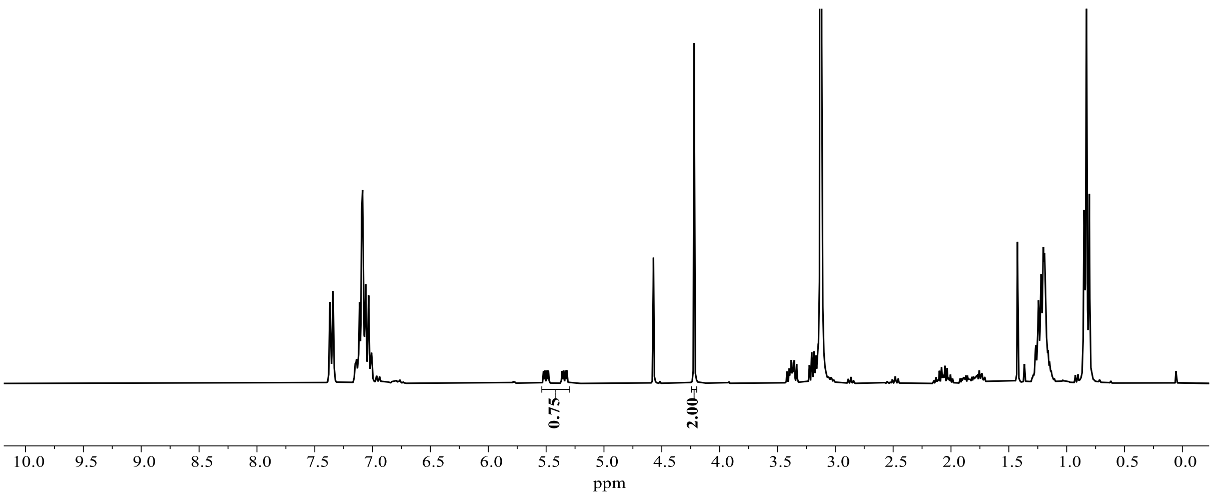

Figure 59 Crude ^{1}H-NMR Spectrum (300 MHz, C_6D_6) of the reaction mixture with CH_2Br_2 (36 µL, 0.50 mmol) as internal standard (4.22 ppm). The signals of the benzylic proton and internal standard are integrated.

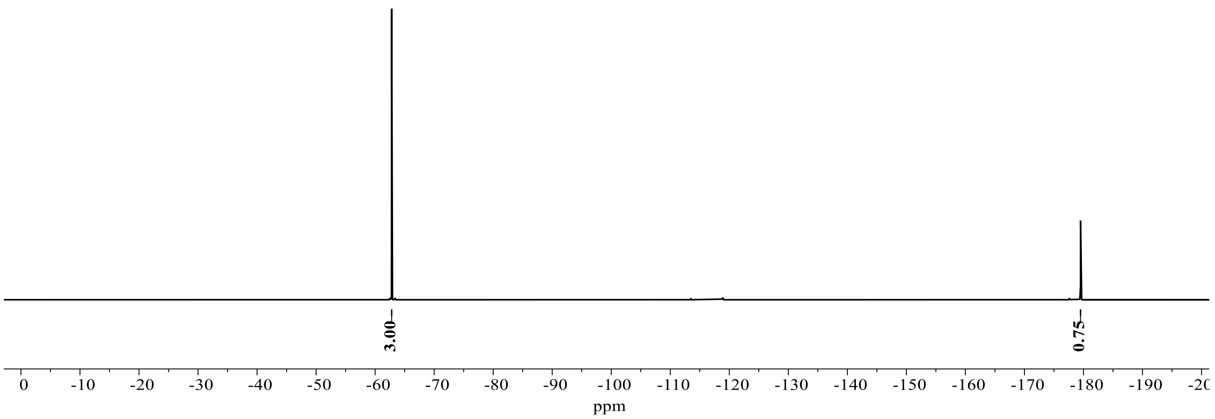

Figure 60 ^{19}F-NMR Spectrum (282 MHz, $CDCl_3$) of the reaction mixture with $PhCF_3$ (62 µL, 0.50 mmol) as internal standard (–62.8 ppm). The signals of the benzylic fluoride and internal standard are integrated.

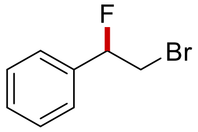

2v

(2-Bromo-1-fluoroethyl)benzene

The general procedure A-1 was followed using (2-bromoethyl)benzene **1v** (92.5 mg, 0.50 mmol) with 3.0 F/mol total charge and a RVC anode. After conducting the NMR analysis, the solvents were removed in vacuo, and the residue was purified by column chromatography (pentane to pentane/Et_2O = 9:1) to obtain the product **2v** as a colorless oil (53.4 mg, 0.26 mmol, 53%).

Benzyl Fluoride C–H Shift: ^{1}H-NMR (400 MHz, $CDCl_3$): δ = 5.63 (ddd, J = 47.0, 4.4, 4.1 Hz, 1H). Calibrated ^{1}H-NMR (300 MHz, d_6-Acetone) yield from benzylic proton: 62%. **Benzylic Fluoride Shift:** ^{19}F{^{1}H}-NMR (282 MHz, $CDCl_3$): δ = –174.0. Calibrated ^{19}F{^{1}H}-NMR (282 MHz, d_6-Acetone) yield from benzylic fluoride: 60%. **HR-MS** (EI) *m/z* calc. for $C_8H_8{}^{79}BrF$ $[M]^+$: 201.9788, found: 201.9786.

The spectral data are in accordance with those reported in literature.[14]

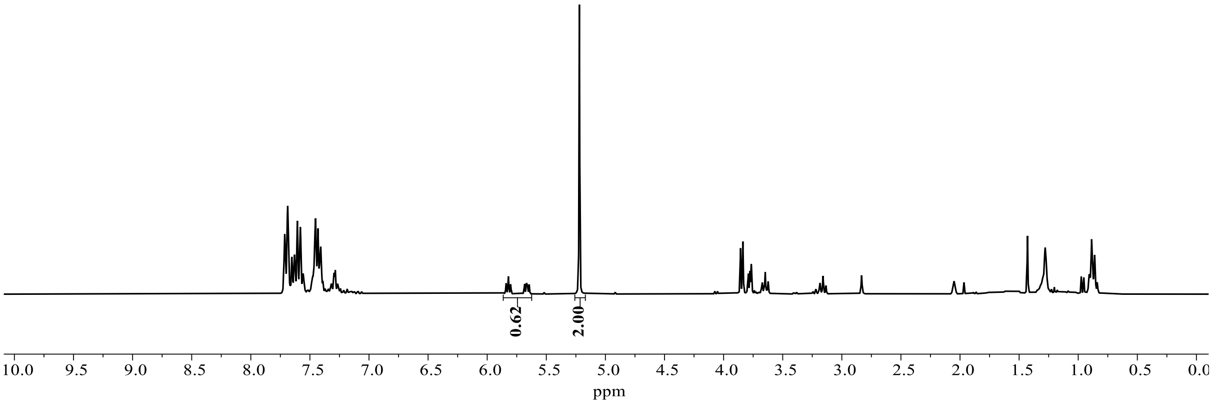

Figure 61 Crude ^{1}H-NMR Spectrum (300 MHz, d_6-Acetone) of the reaction mixture with CH_2Br_2 (36 µL, 0.50 mmol) as internal standard (5.25 ppm). The signals of the benzylic proton and internal standard are integrated.

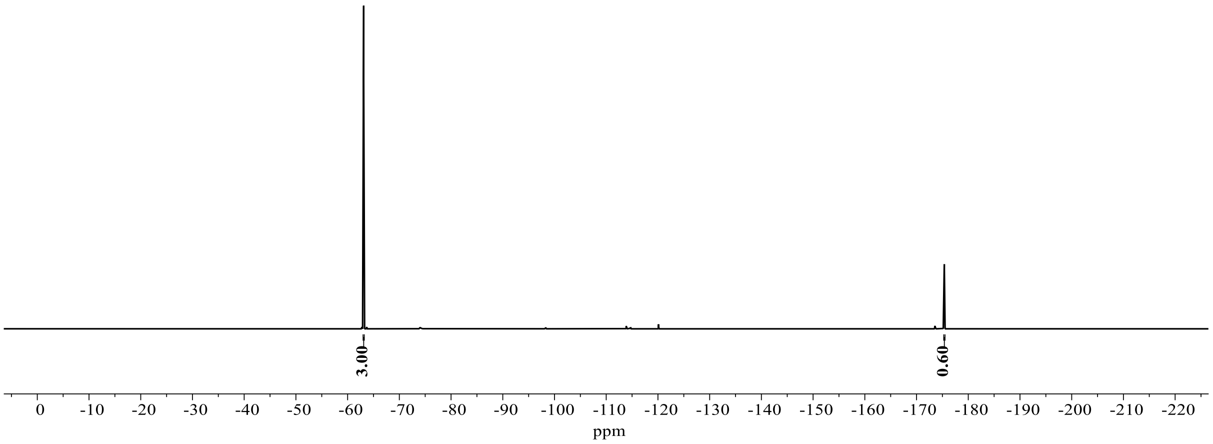

Figure 62 Crude ^{19}F-NMR Spectrum (282 MHz, d_6-Acetone) of the reaction mixture with $PhCF_3$ (62 µL, 0.50 mmol) as internal standard (–63.1 ppm). The signals of the benzylic fluoride and internal standard are integrated.

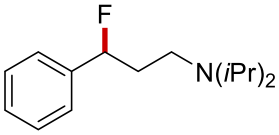

2w

3-Fluoro-*N*,*N*-diisopropyl-3-phenylpropan-1-amine

The general procedure A-2 was followed using *N*,*N*-diisopropyl-3-phenylpropan-1-amine **1w** (110.0 mg, 0.50 mmol) at 0 °C and 10 mA with 4.0 F/mol total charge. The combined organic solutions were washed the first time with sat. aq. $NaHCO_3$ (20 mL) instead of water. After conducting the NMR analysis, the solvents were removed in vacuo, and the residue was purified by column chromatography on partially neutralized silica (*n*hexane/EtOAc/NEt_3 = 35:4:1) to obtain the product **2w** as a colorless oil (39.4 mg, 0.16 mmol, 33%).

^{1}H-NMR (300 MHz, $CDCl_3$) δ =7.43–7.26 (m, 5H), 5.57 (ddd, J = 48.2, 8.8, 3.8 Hz, 1H), 3.01 (hept, J = 7.0 Hz, 2H), 2.61 (dd, J = 8.0, 6.2 Hz, 2H), 2.14 – 1.75 (m, 2H), 1.01 (t, J = 7.0 Hz, 12H). Calibrated ^{1}H-NMR (300 MHz, C_6D_6) yield from benzylic proton: 49%. **^{19}F{^{1}H}-NMR** (282 MHz, $CDCl_3$): δ = −176.4. Calibrated ^{19}F{^{1}H}-NMR (282 MHz, C_6D_6) yield from benzylic fluoride: 54%. **^{13}C-NMR** (101 MHz, $CDCl_3$): δ = 141.0 (d, $^2J_{C-F}$ = 19.7 Hz, C_q), 128.5 (CH), 128.2 (d, $^5J_{C-F}$ = 1.9 Hz, CH), 125.7 (d, $^3J_{C-F}$ = 6.8 Hz, CH), 93.0 (d, $^1J_{C-F}$ = 169.0 Hz, CH), 48.6 (CH), 41.0 (d, $^3J_{C-F}$ = 3.5 Hz), 39.0 (d, $^2J_{C-F}$ = 22.9 Hz), 21.4 (CH_3), 20.5 (CH_3). **IR** (ATR): $\tilde{\nu}$ = 2965, 2931, 2873, 1456, 1388, 1363, 1203, 1171, 1056, 910, 756, 699. **HR-MS** (EI) *m/z* calc. for $C_{15}H_{24}FN$ $[M]^+$: 237.1893, found: 237.1887.

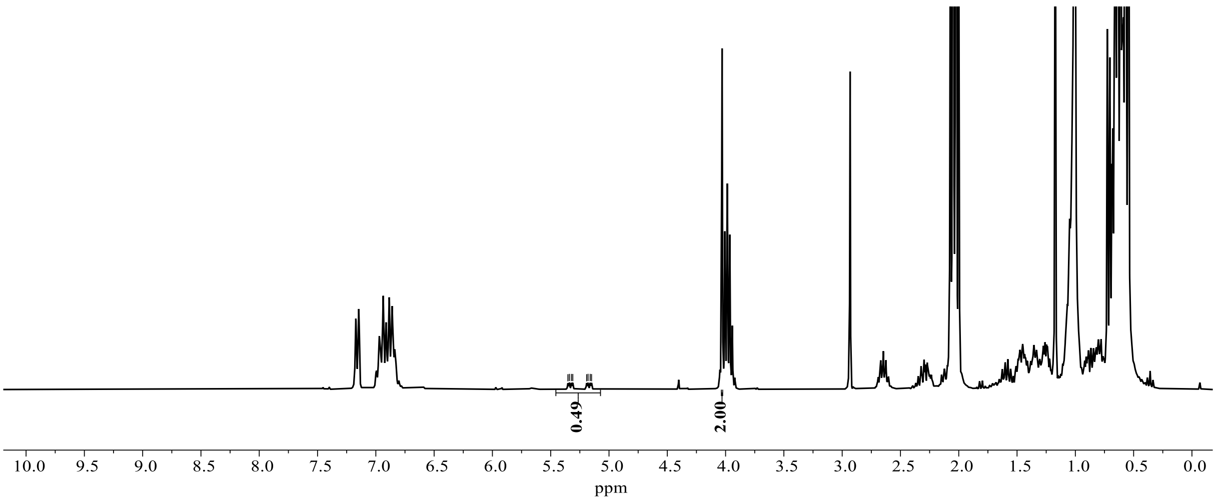

Figure 63 Crude ^{1}H-NMR Spectrum (300 MHz, C_6D_6) of the reaction mixture with CH_2Br_2 (36 µL, 0.50 mmol) as internal standard (4.03 ppm). The signals of the benzylic proton and internal standard are integrated.

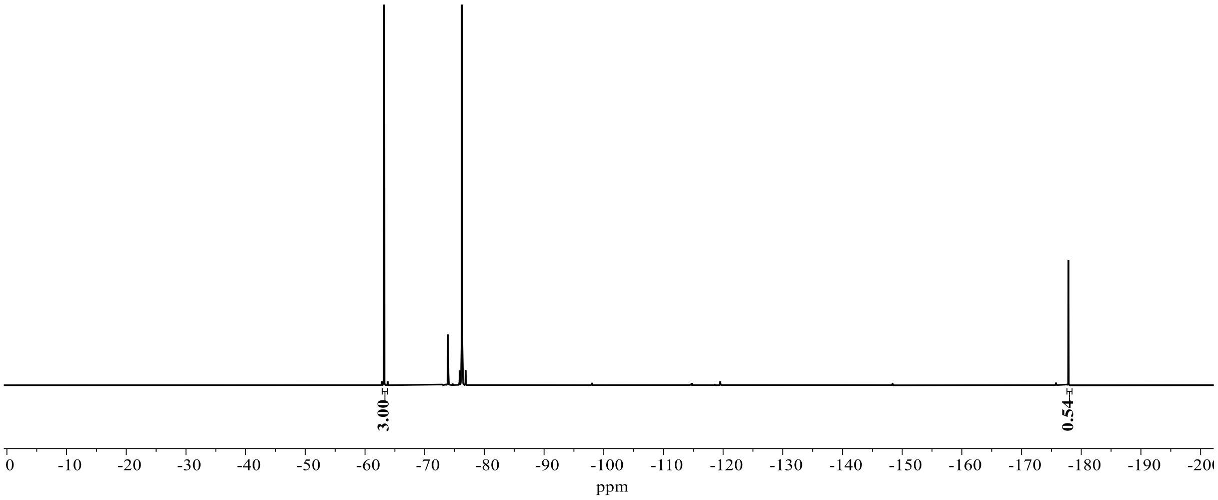

Figure 64 Crude $^{19}F\{^{1}H\}$-NMR Spectrum (282 MHz, C_6D_6) of the reaction mixture with $PhCF_3$ (62 µL, 0.50 mmol) as internal standard (–63.5 ppm). The signals of the benzylic fluoride and internal standard are integrated.

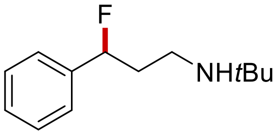

2x

***N*-(*tert*-butyl)-3-fluoro-3-phenylpropan-1-amine**

The general procedure A-2 was followed using *N*-(*tert*-butyl)-3-phenylpropan-1-amine **1x** (95.9 mg, 0.50 mmol) at 0 °C with 2.5 F/mol total charge. The combined organic solutions were washed the first time with sat. aq. $NaHCO_3$ (20 mL) instead of water. After conducting the NMR analysis, the solvents were removed in vacuo, and the residue was purified by column chromatography on partially neutralized silica (*n*hexane/acetone/NEt_3 = 30:4:1) to obtain the product **2x** as a colorless oil (37.9 mg, 0.18 mmol, 36%).

^{1}H-NMR (400 MHz, $CDCl_3$) δ = Calibrated ^{1}H-NMR yield from benzylic proton: 48%. **^{19}F{^{1}H}-NMR** (282 MHz, $CDCl_3$): δ = −175.6. Calibrated ^{19}F{^{1}H}-NMR yield from benzylic fluoride: 52%. **^{13}C-NMR** (101 MHz, $CDCl_3$): δ = 140.4 (d, $^2J_{C–F}$ = 19.7 Hz), 128.6 (CH), 128.4 (d, $^5J_{C–F}$ = 2.0 Hz, CH), 125.6 (d, $^3J_{C–F}$ = 6.9 Hz, CH), 93.5 (d, $^1J_{C–F}$ = 169.9 Hz, CH), 50.5 (C_q), 38.8 (d, $^3J_{C–F}$ = 4.0 Hz, CH_2), 38.6 (d, $^2J_{C–F}$ = 23.2 Hz, CH_2), 29.1 (CH_3). **IR** (ATR): $\tilde{\nu}$ = 2963, 2868, 1453, 1361, 1214, 1102, 972, 913, 758, 699, 552. **HR-MS** (EI) *m/z* calc. for $C_{13}H_{21}FN$ $[M+H]^+$: 210.1653, found: 210.1653.

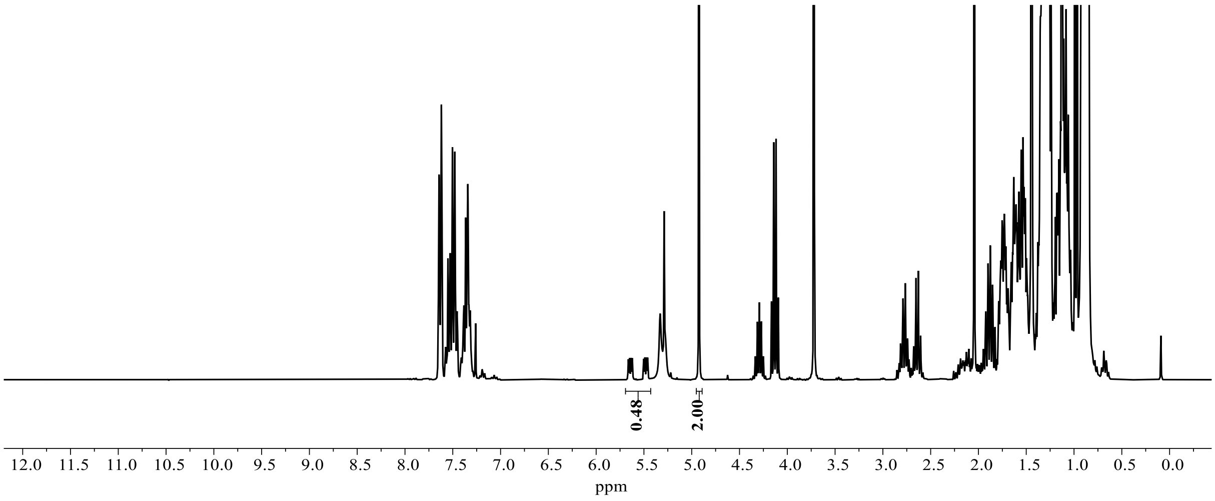

Figure 65 Crude ^{1}H-NMR Spectrum (300 MHz, $CDCl_3$) of the reaction mixture with CH_2Br_2 (36 µL, 0.50 mmol) as internal standard (4.93 ppm). The signals of the benzylic proton and internal standard are integrated.

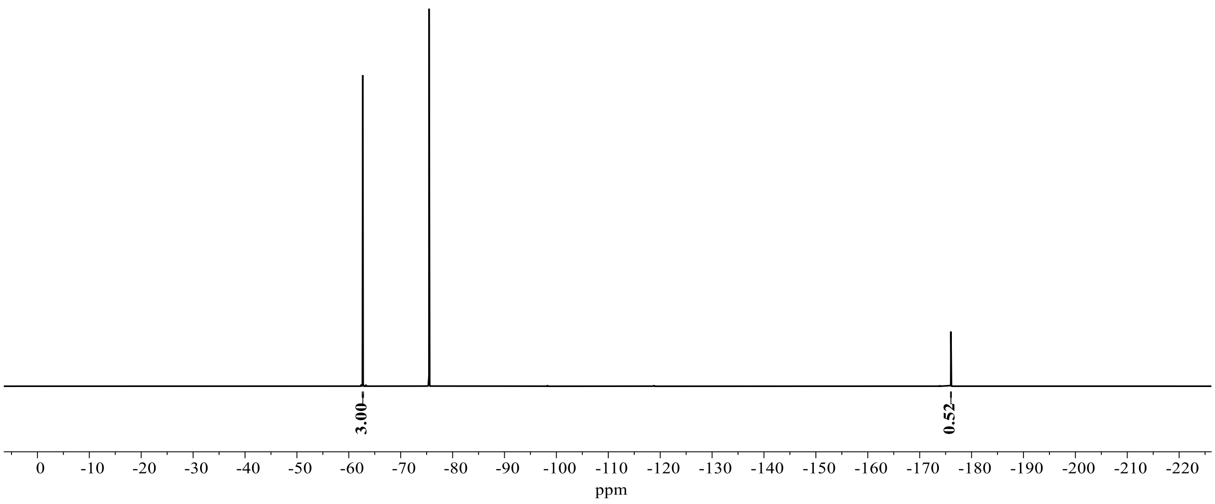

Figure 66 ^{19}F-NMR Spectrum (282 MHz, $CDCl_3$) of the reaction mixture with $PhCF_3$ (62 µL, 0.50 mmol) as internal standard (–62.7 ppm). The signals of the benzylic fluoride and internal standard are integrated.

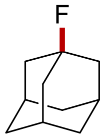

2y

1-Fluoroadamantane

The general procedure A-1 was followed using adamantane **1y** (68.1 mg, 0.50 mmol) at 0 °C.

Aliphatic Fluoride Shift: $^{19}F\{^{1}H\}$-NMR (282 MHz, C_6D_6) δ = –128.6. Calibrated $^{19}F\{^{1}H\}$-NMR yield from benzylic fluoride: 57%. **HR-MS** (EI) *m/z* calc. for $C_{10}H_{15}F$ $[M]^+$: 154.1152, found: 154.1152.

The spectral data are in accordance with those reported in literature.[15]

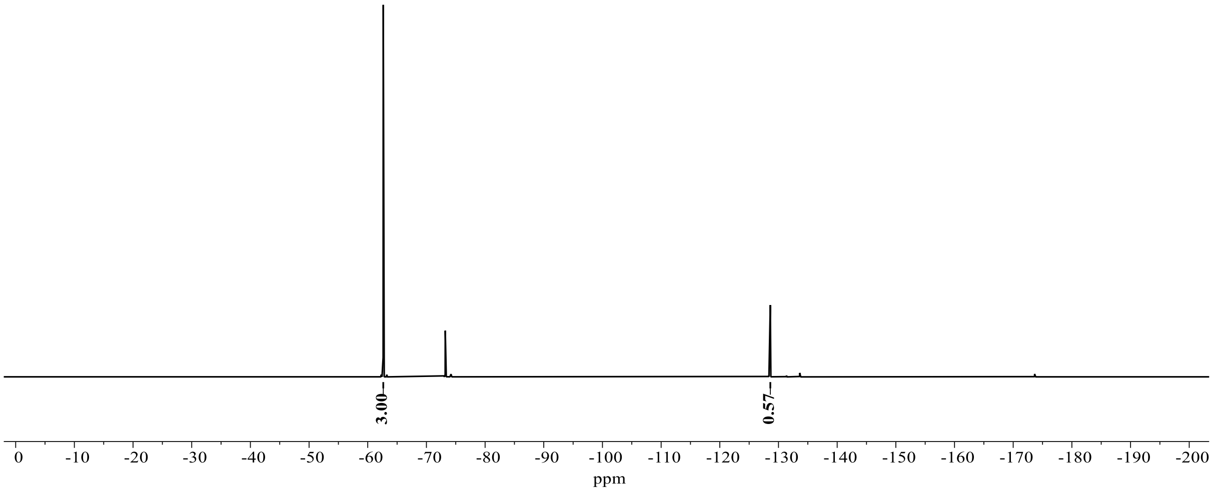

Figure 67 ^{19}F-NMR Spectrum (282 MHz, C_6D_6) of the reaction mixture with $PhCF_3$ (62 µL, 0.50 mmol) as internal standard (–62.6 ppm). The signals of the benzylic fluoride and internal standard are integrated.

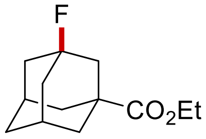

2z

Ethyl 3-fluoroadamantane-1-carboxylate

The general procedure A-2 was followed using ethyl adamantane-1-carboxylate **1z** (104 mg, 0.50 mmol) at −20 °C with 4.0 F/mol total charge. After conducting the NMR analysis, the solvents were removed in vacuo, and the residue was purified by column chromatography on partially neutralized silica (*n*hexane/EtOAc = 25:1) to obtain the product **2z** as a colorless oil (47.8 mg, 0.21 mmol, 42%).

^{1}H-NMR (300 MHz, $CDCl_3$): δ = 4.12 (q, J = 7.1 Hz, 2H), 2.39–2.29 (m, 2H), 2.02 (d, J = 5.8 Hz, 2H), 1.87 (dd, J = 5.7, 3.3 Hz, 4H), 1.83–1.73 (m, 4H), 1.60 (ddd, J = 3.0 Hz, 2H), 1.24 (t, J = 7.1 Hz, 3H). **^{19}F{^{1}H}-NMR** (282 MHz, $CDCl_3$): δ = −132.3. Calibrated ^{19}F{^{1}H}-NMR (282 MHz, CD_2Cl_2) yield from aliphatic fluoride: 48%. **^{13}C-NMR** (101 MHz, $CDCl_3$): δ = 175.9 (d, $^4J_{C-F}$ = 2.1 Hz, C_q), 92.4 (d, $^1J_{C-F}$ = 183.9 Hz, C_q), 60.6 (CH_2), 45.0 (d, $^3J_{C-F}$ = 10.2 Hz, C_q), 43.8 (d, $^2J_{C-F}$ = 19.9 Hz, CH_2), 42.0 (d, $^2J_{C-F}$ = 17.4 Hz, CH_2), 37.7 (d, $^4J_{C-F}$ = 2.0 Hz, CH_2), 35.0 (d, $^4J_{C-F}$ = 2.1 Hz), 31.0 (d, $^3J_{C-F}$ = 10.0 Hz, CH), 14.3 (CH_3). **IR** (ATR): $\tilde{\nu}$ = 2920, 2864, 1725, 1456, 1251, 1225, 1095, 1028, 939, 894, 548. **HR-MS** (EI) *m/z* calc. for $C_{13}H_{19}FO_2$ $[M]^+$: 226.1369, found: 226.1364.

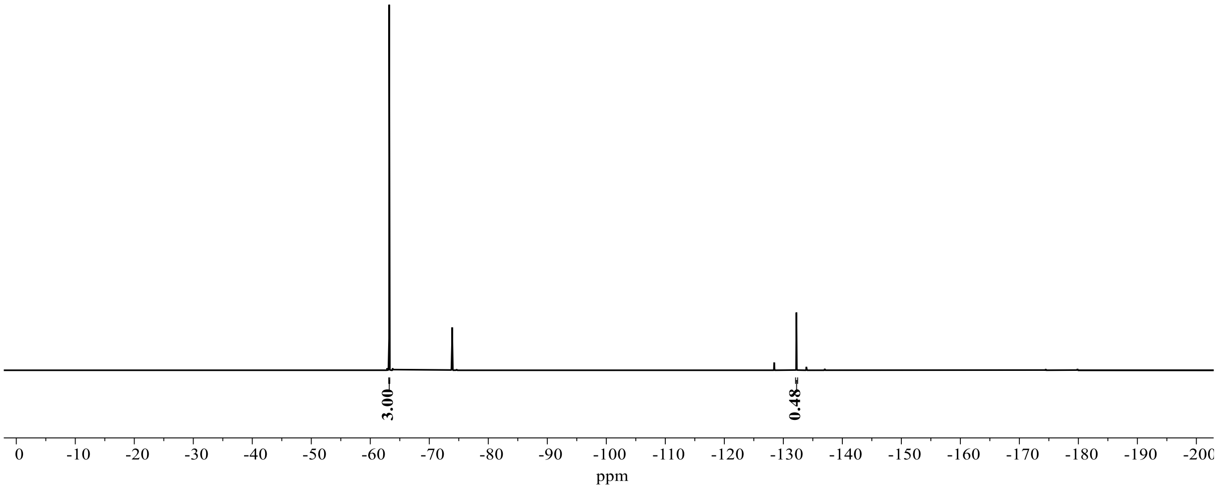

Figure 68 Crude ^{19}F{^{1}H}-NMR Spectrum (282 MHz, CD_2Cl_2) of the reaction mixture with $PhCF_3$ (62 µL, 0.50 mmol) as internal standard (–63.2 ppm). The signals of the benzylic fluoride and internal standard are integrated.

11.Late-Stage Fluorination

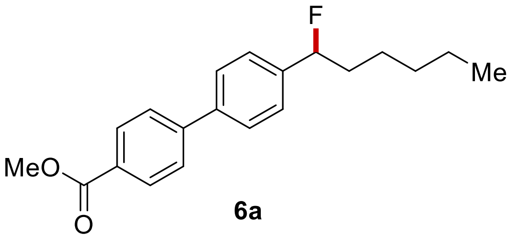

6a

Methyl-4'-(1-fluorohexyl)-[1,1'-biphenyl]-4-carboxylate

The general procedure A was followed using methyl-4'-hexyl-[1,1'-biphenyl]-4-carboxylate **5a** (148 mg, 0.50 mmol). The spectral data are in accordance with those reported in literature. After conducting the NMR analysis, the solvents were removed in vacuo, and the residue was purified by column chromatography (*n*hexane/EtOAc = 25/1) to obtain the product **6a** as a colorless solid (134 mg, 0.43 mmol, 85%).

M.p.: 198–200 °C. **^{1}H-NMR** (400 MHz, C_6D_6): δ = 8.19–8.15 (m, 2H), 7.44–7.33 (m, 4H), 7.25 (d, J = 8.0 Hz, 2H), 5.31 (ddd, J = 48.0, 5.0, 4.7 Hz, 1H), 3.57 (s, 3H), 1.96–1.81 (m, 1H), 1.75–1.58 (m, 1H), 1.49–1.25 (m, 2H), 1.19 (m, 4H), 0.85 (t, J = 6.8 Hz, 3H). Calibrated ^{1}H-NMR yield from benzylic proton: 83%. **^{13}C-NMR** (101 MHz, C_6D_6): δ = 166.6 (C_q), 145.2 (C_q), 141.2 (d, $^2J_{C–F}$ = 20.0 Hz, C_q), 140.0 (d, $^5J_{C–F}$ = 1.9 Hz, C_q), 130.5 (CH), 129.8 (C_q), 127.6 (CH), 127.3 (CH), 126.4 (d, $^3J_{C–F}$ = 6.9 Hz, CH), 94.3 (d, $^1J_{C–F}$ = 171.7 Hz, CH), 51.7 (CH_3), 37.7 (d, $^2J_{C–F}$ = 23.5 Hz, CH_2), 31.9 (CH_2), 25.2 (d, $^3J_{C–F}$ = 4.1 Hz, CH_2), 22.9 (CH_2), 14.2 (CH_3). **^{19}F{^{1}H}-NMR** (282 MHz, $CDCl_3$): δ = –175.3. Calibrated ^{19}F{^{1}H}-NMR yield from benzylic fluoride: 93%. **IR** (ATR): $\tilde{\nu}$ = 2956, 2923, 1719, 1432, 1282, 1222, 1187, 1109, 824, 767. **M.p.**: 89–91 °C. **HR-MS** (ESI) *m/z* calc. for $C_{20}H_{23}FO_2$ $[M+Na]^+$: 314.1677, found: 314.1677.

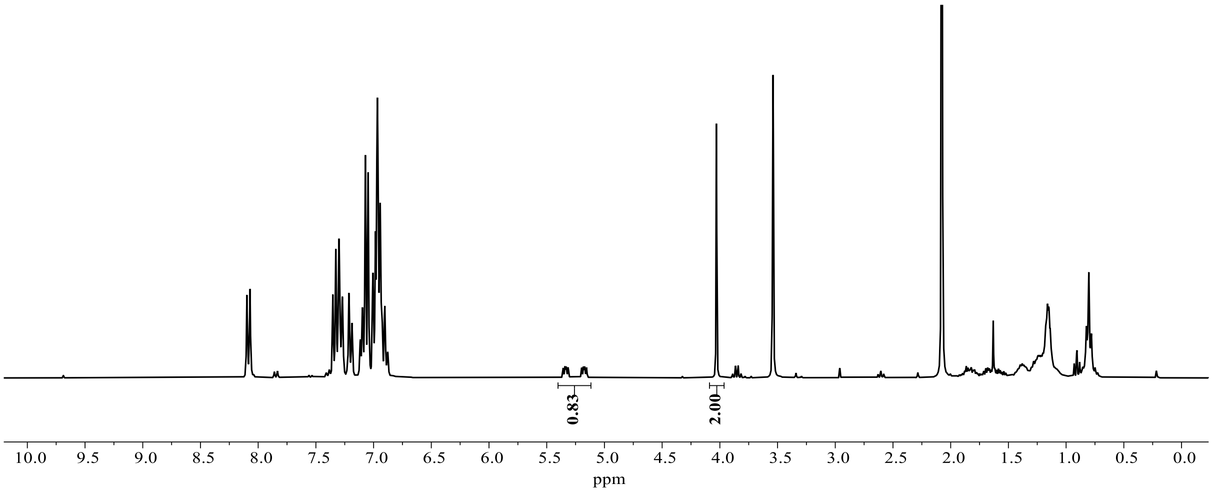

Figure 69 Crude ^{1}H-NMR Spectrum (300 MHz, C_6D_6) of the reaction mixture with CH_2Br_2 (36 µL, 0.50 mmol) as internal standard (4.03 ppm). The signals of the benzylic proton and internal standard are integrated.

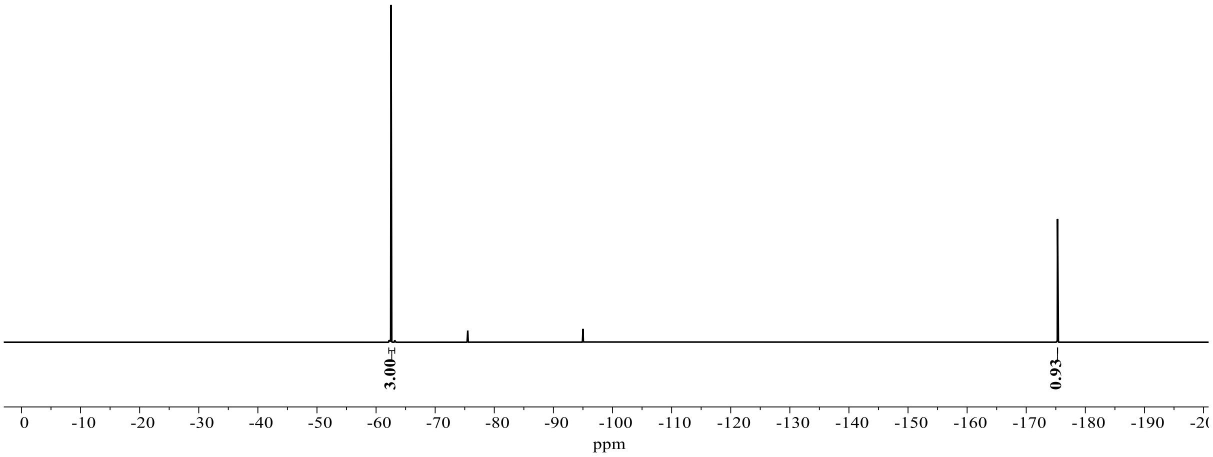

Figure 70 ^{19}F-NMR Spectrum (282 MHz, C_6D_6) of the reaction mixture with $PhCF_3$ (62 µL, 0.50 mmol) as internal standard (–62.5 ppm). The signals of the benzylic fluoride and internal standard are integrated.

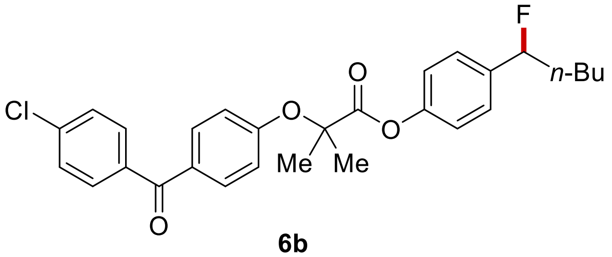

6b

4-(1-fluoropentyl)phenyl 2-(4-(4-chlorobenzoyl)phenoxy)-2-methylpropanoate

The general procedure A-2 was followed using ethyl 4-pentylphenyl 2-(4-(4-chlorobenzoyl)phenoxy)-2-methylpropanoate **5b** (232 mg, 0.50 mmol) at −20 °C and 20 mA with 4.0 F/mol total charge. After conducting the crude NMR analysis, the solvents were removed in vacuo, and the residue was purified by column chromatography (*n*hexane/EtOAc = 15/1), to obtain the product **6b** (102 mg, 0.21 mmol, 42%) of a colorless solid.

M.p.: 64–66 °C. **^{1}H-NMR** (400 MHz, $CDCl_3$)**:** δ = 7.81–7.76 (m, 2H), 7.74–7.70 (m, 2H), 7.48–7.44 (m, 2H), 7.35–7.30 (m, 2H), 7.02–6.97 (m, 4H), 5.41 (ddd, J = 47.7, 8.0, 4.9, 1H), 2.12–1.65 (m, 2H), 1.83 (s, 6H) 1.48–1.23 (m, 4H), 0.90 (t, J = 6.8, 3H). Calibrated ^{1}H-NMR yield from benzylic proton: 51%. **^{13}C-NMR** (126 MHz, $CDCl_3$): δ = 194.3 (C_q), 172.5 (C_q), 159.7 (C_q), 150.2 (C_q), 138.9 (d, $^2J_{C-F}$ = 20.2, C_q), 138.6 (C_q), 136.4 (C_q), 132.3 (CH), 131.3 (CH), 130.9 (C_q), 128.7 (CH), 126.9 (d, $^3J_{C-F}$ = 6.9, CH), 121.3 (CH), 117.5 (CH), 94.1 (d, $^1J_{C-F}$ = 171.1, CH), 79.6 (C_q), 37.1 (d, $^2J_{C-F}$ = 23.2, CH_2), 27.3 (d, $^3J_{C-F}$ = 4.2, CH_2), 25.6 (CH_3), 25.6 (CH_3), 22.6 (CH_2), 14.6 (CH_3). ^{19}F{^{1}H}-NMR (282 MHz, $CDCl_3$): δ = −174.1. Calibrated ^{19}F{^{1}H}-NMR yield from benzylic fluoride: 50%. **IR** (ATR): $\tilde{\nu}$ = 2529, 2873, 1771, 1645, 1596, 1594, 1193, 1166, 1087, 853, 477. **HR-MS** (ESI) *m/z* calc. for $C_{28}H_{28}ClFO_4Na$ $[M+Na]^+$: 505.1558, found: 505.1552.

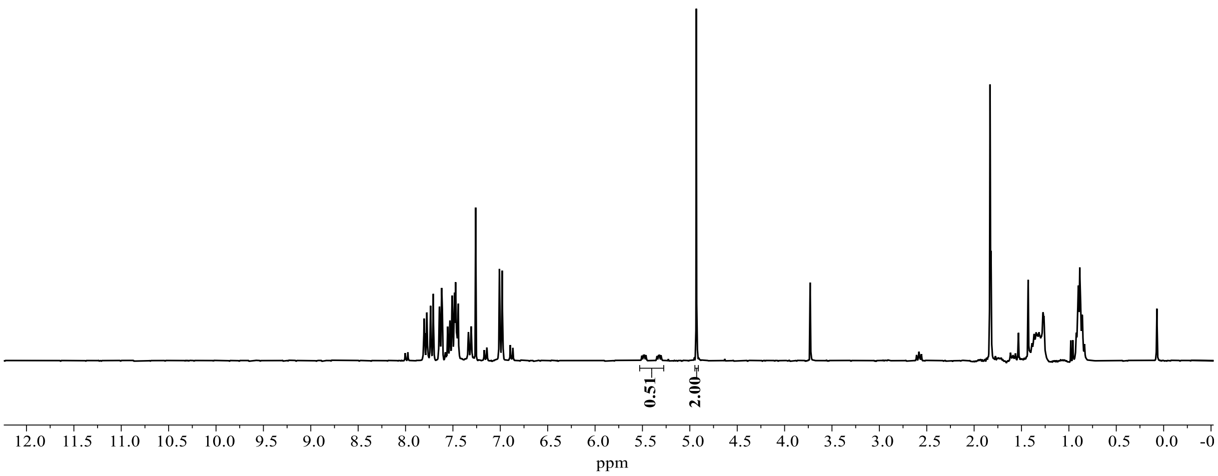

Figure 71 Crude ^{1}H-NMR Spectrum (300 MHz, $CDCl_3$) of the reaction mixture with CH_2Br_2 (36 µL, 0.50 mmol) as internal standard (4.93 ppm). The signals of the benzylic proton and internal standard are integrated.

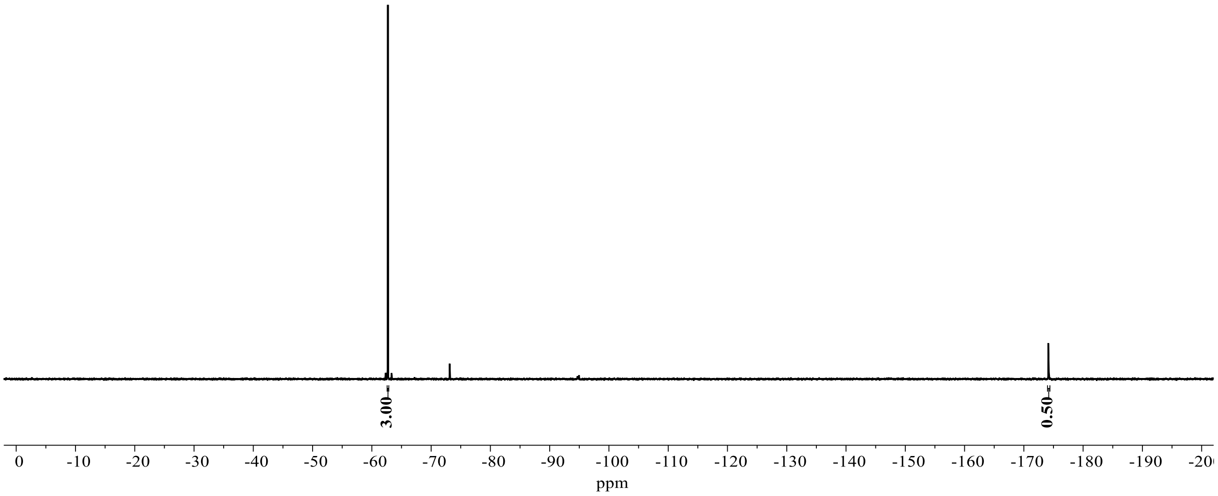

Figure 72 Crude $^{19}F\{^{1}H\}$-NMR Spectrum (282 MHz, $CDCl_3$) of the reaction mixture with $PhCF_3$ (62 µL, 0.50 mmol) as internal standard (–62.7 ppm). The signals of the benzylic fluoride and internal standard are integrated.

12. C(sp^3)–H Arylation

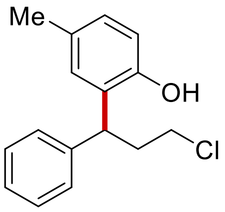

8ua

(2-(3-chloro-1-phenylpropyl)-4-methylphenol

The general procedure B was followed using (3-chloro-1-propyl)benzene **1u** (77.3 mg, 0.50 mmol) with 3.0 F/mol total charge and 4-methylphenol **7a** (270 mg, 2.50 mmol). After the mixture was stirred overnight another portion of HFIP (0.5 mL) was added, and the reaction was stirred for another 6 h at 50 °C. After the reaction was finished, the solvents were removed in vacuo and the residue was purified by column chromatography on silica gel (EtOAc/*n*hexane = 2:98) to furnish **6ua** (72.3 mg, 0.27 mmol, 55%) as a colorless oil.

^{1}H-NMR (400 MHz, $CDCl_3$): δ = 7.40–7.33 (m, 4H), 7.30–7.25 (m, 1H), 7.09 (d, J = 2.1 Hz, 1H), 6.97 (dd, J = 8.0, 2.1 Hz, 1H), 6.69 (d, J = 8.0 Hz, 1H), 4.68 (brs, 1H), 4.53 (t, J = 7.8 Hz, 1H), 3.56 (m, 2H), 2.66–2.50 (m, 2H), 2.35 (s, 3H). **^{13}C-NMR** (101 MHz, $CDCl_3$): δ = 151.2 (C_q), 143.0 (C_q), 130.3 (C_q), 129.5 (C_q), 128.8 (CH), 128.7 (CH), 128.3 (CH), 128.1 (CH), 126.8 (CH), 116.2 (CH), 43.4 (CH_2), 41.5 (CH), 37.3 (CH_2), 20.9 (CH_3). **IR** (ATR): $\tilde{\nu}$ = 1500, 1447, 1256, 1184, 1103, 811, 763, 734, 701, 518. **MS** (ESI) *m/z* (relative intensity): 225 (100) [M-Cl]$^+$, 283 (90) [M+Na]$^+$. **HR-MS** (ESI) *m/z* calc. for $C_{16}H_{17}O^{35}Cl$ [M+Na]$^+$: 283.0860, found: 283.0846.

The spectral data are in accordance with those reported in literature.[7]

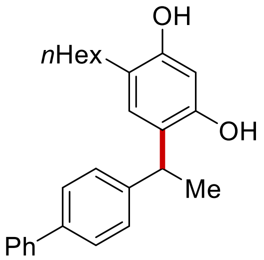

8ob

4-(1-([1,1'-biphenyl]-4-yl)ethyl)-6-hexylbenzene-1,3-diol

The general procedure B was followed using 4-ethyl-1,1'-biphenyl **1o** (91.2 mg, 0.50 mmol) and 4-hexylbenzene-1,3-diol **7b** (485 mg, 2.50 mmol). After the reaction was finished, the

solvents were removed in vacuo and the residue was purified by column chromatography on silica gel (EtOAc/*n*hexane = 1:8 to 1:2) to furnish **8ob** (106 mg, 0.28 mmol, 57%) as a colorless oil.

^{1}H-NMR (400 MHz, $CDCl_3$): δ = 7.40–7.33 (m, 4H), 7.30–7.25 (m, 1H), 7.09 (d, *J* = 2.1 Hz, 1H), 6.97 (dd, *J* = 8.0, 2.1 Hz, 1H), 6.69 (d, *J* = 8.0 Hz, 1H), 4.68 (brs, 1H), 4.53 (t, *J* = 7.8 Hz, 1H), 3.56 (m, 2H), 2.66–2.50 (m, 2H), 2.35 (s, 3H). **^{13}C-NMR** (101 MHz, $CDCl_3$): δ = 151.2 (C_q), 143.0 (C_q), 130.3 (C_q), 129.5 (C_q), 128.8 (CH), 128.7 (CH), 128.3 (CH), 128.1 (CH), 126.8 (CH), 116.2 (CH), 43.4 (CH_2), 41.5 (CH), 37.3 (CH_2), 20.9 (CH_3). **IR** (ATR): $\tilde{\nu}$ = 1500, 1447, 1256, 1184, 1103, 811, 763, 734, 701, 518. **MS** (ESI) *m/z* (relative intensity): 225 (100) $[M-Cl]^+$, 283 (90) $[M+Na]^+$. **HR-MS** (ESI) *m/z* calc. for $C_{16}H_{17}O^{35}Cl$ $[M+Na]^+$: 283.0860, found: 283.0846.

The spectral data are in accordance with those reported in literature.[7]

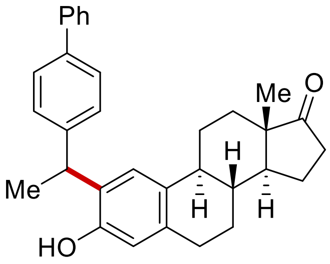

8oc

(8R,9S,13S,14S)-2-(1-([1,1'-biphenyl]-4-yl)ethyl)-3-hydroxy-13-methyl-6,7,8,9,11,12,13,14,15,16-decahydro-17H-cyclopenta[a]phenanthren-17-one

The general procedure B was followed using 4-ethyl-1,1'-biphenyl **1o** (91.2 mg, 0.50 mmol), estrone **7c** (540 mg, 2.00 mmol) and DCE/HFIP (3.5 mL, 6:1) for the benzylation reaction. After the reaction was finished, the solvents were removed in vacuo and the residue was purified by column chromatography on silica gel (EtOAc/*n*hexane = 1:6 to 1:3) to furnish **8oc** (153 mg, 0.34 mmol, 68%) as a colorless solid. **M.p.**: 198 – 199 °C **^{1}H-NMR** (600 MHz, $CDCl_3$): δ = 7.59–7.56 (m, 2H), 7.55–7.52 (m, 2H), 7.44–7.40 (m, 2H), 7.38–7.35 (m, 2H), 7.34–7.31 (m, 1H), 7.24 (s, 1H), 7.20 (d, *J* = 7.1 Hz, 1H), 6.54 (d, *J* = 6.3 Hz, 1H), 5.26–5.19 (m, 1H), 4.45 (dq, *J* = 12.0, 7.2 Hz, 1H), 2.91–2.78 (m, 2H), 2.55–2.48 (m, 1H), 2.44–2.39 (m, 1H), 2.28 (qd, *J* = 11.1, 4.4 Hz, 1H), 2.20–2.12 (m, 1H), 2.08–2.02 (m, 1H), 2.01–1.96 (m, 2H), 1.68 (dd, *J* = 7.3, 3.3 Hz, 3H), 1.65–1.57 (m, 2H), 1.57–1.51 (m, 2H), 1.46–1.39 (m, 1H), 0.93 (d, *J* = 5.7 Hz, 3H). **^{13}C-NMR** (151 MHz, $CDCl_3$, mixture of diastereomers): δ = 221.9 (C_q), 151.4 (C_q), 145.0 (C_q), 144.9 (C_q), 141.0 (C_q), 141.0 (C_q), 139.1 (C_q), 139.1 (C_q), 135.7 (C_q), 135.7 (C_q), 131.9 (C_q), 131.8 (C_q), 129.6 (C_q), 129.6 (C_q), 128.8 (CH), 128.0 (CH), 128.0 (CH),

127.3 (CH), 127.3 (CH), 127.2 (CH), 127.1 (CH), 125.0 (CH), 124.9 (CH), 116.0 (CH), 116.0 (CH), 50.5 (CH), 48.2 (CH_2), 48.2 (CH_2), 44.3 (CH), 44.2 (CH), 38.5 (CH), 38.5 (CH), 38.4 (CH), 38.2 (CH_2), 36.0 (CH_2), 31.7 (CH_2), 29.2 (CH_2), 29.1 (CH_2), 26.6 (CH_2), 26.6 (CH_2), 26.1 (CH_2), 26.1 (CH_2), 21.7 (CH_2), 21.7 (CH_2), 21.2 (CH_3), 21.1 (CH_3), 14.0 (CH_3), 14.0 (CH_3). **IR** (ATR): $\tilde{\nu}$ = 2928, 1724, 1508, 1488, 1420, 1254, 1216, 838, 755, 698. **MS** (ESI) *m/z* (relative intensity): 473 (100) $[M+Na]^+$, 923 (60) $[2M+Na]^+$, 1378 $[3M+Na]^+$. **HR-MS** (ESI) *m/z* calc. for $C_{32}H_{34}O_2$ $[M+Na]^+$: 473.2451, found: 473.2441.

13. Unsuccessful Substrates

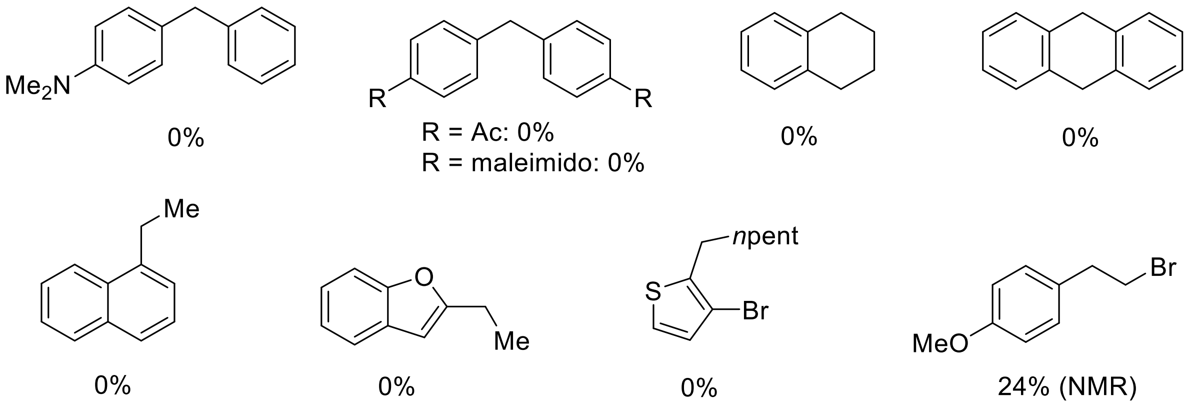

Figure 73 List of substrates which did not undergo the electrochemical benzylic fluorination or gave only poor yields.

14.NMR Spectra

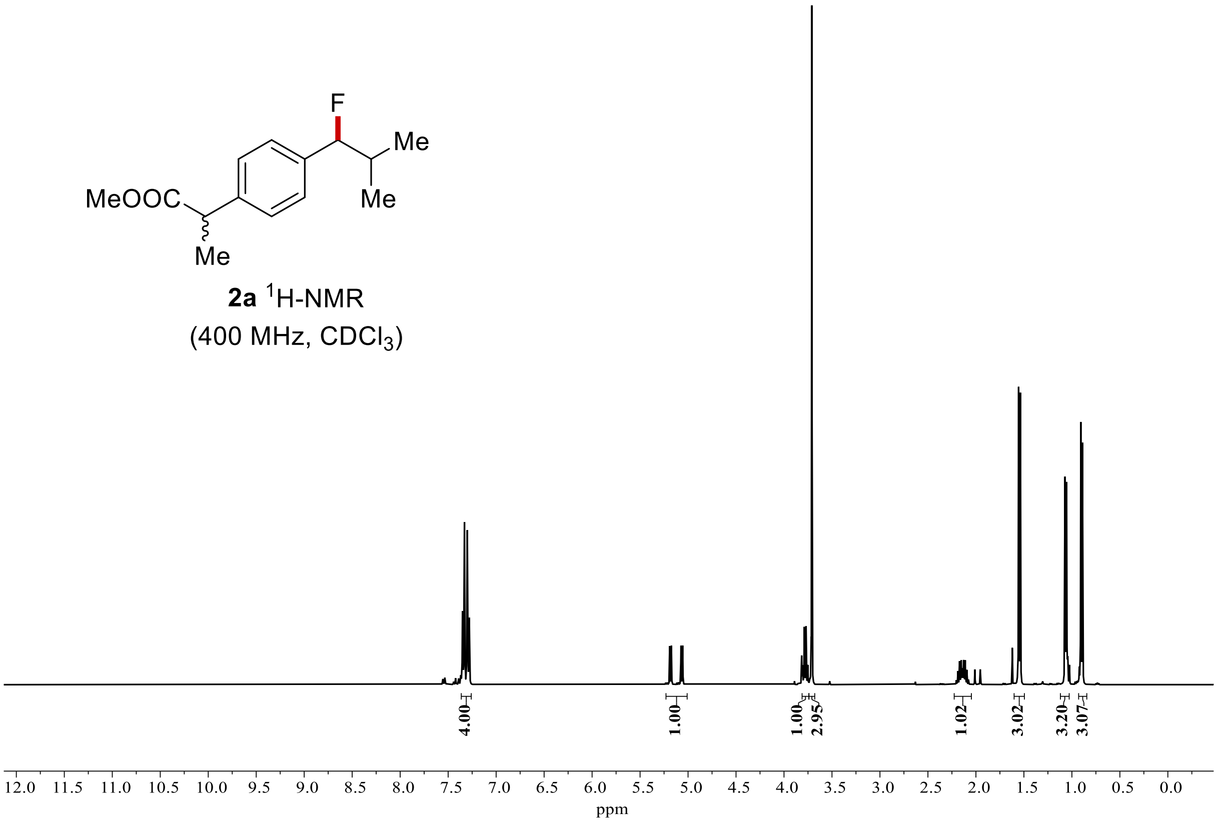

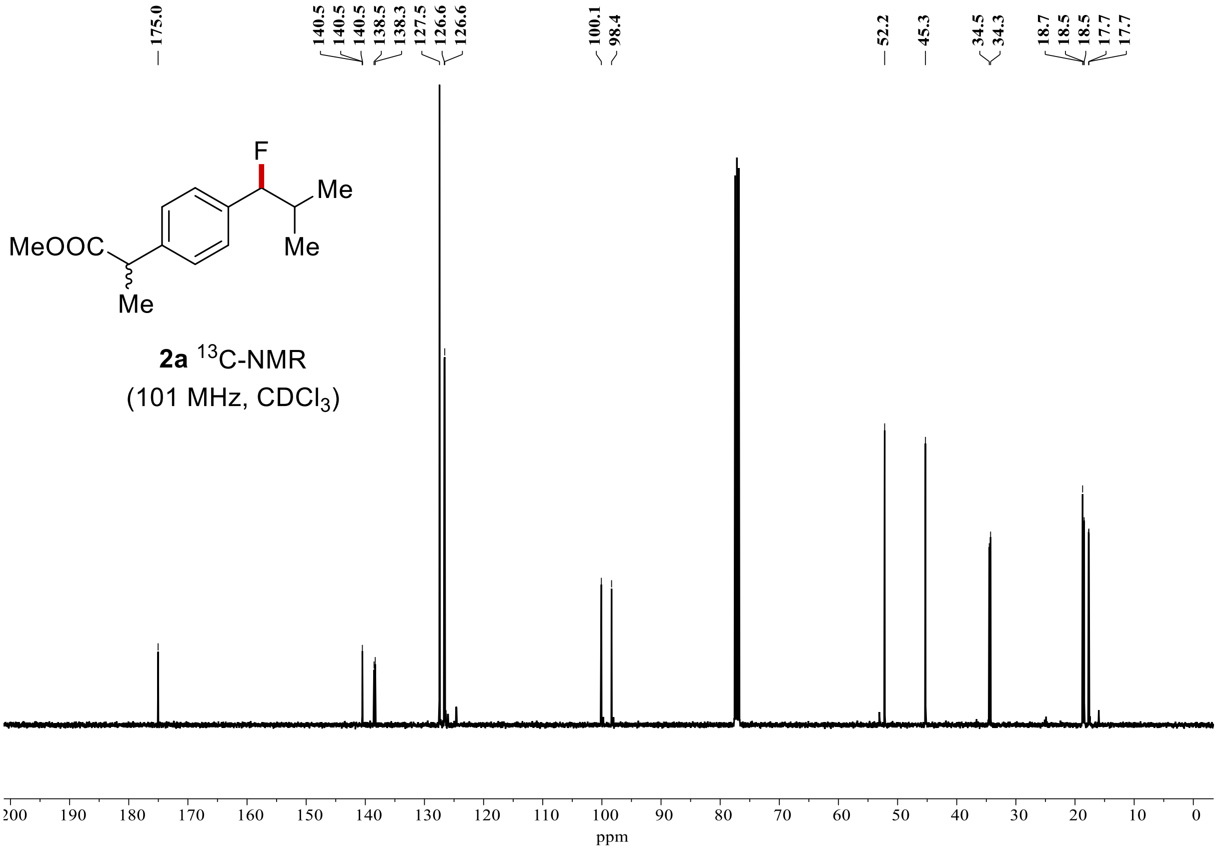

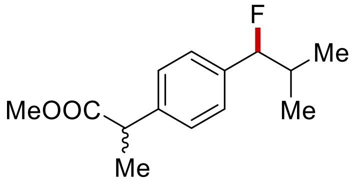

2a ^{19}F-NMR
(282 MHz, $CDCl_3$)

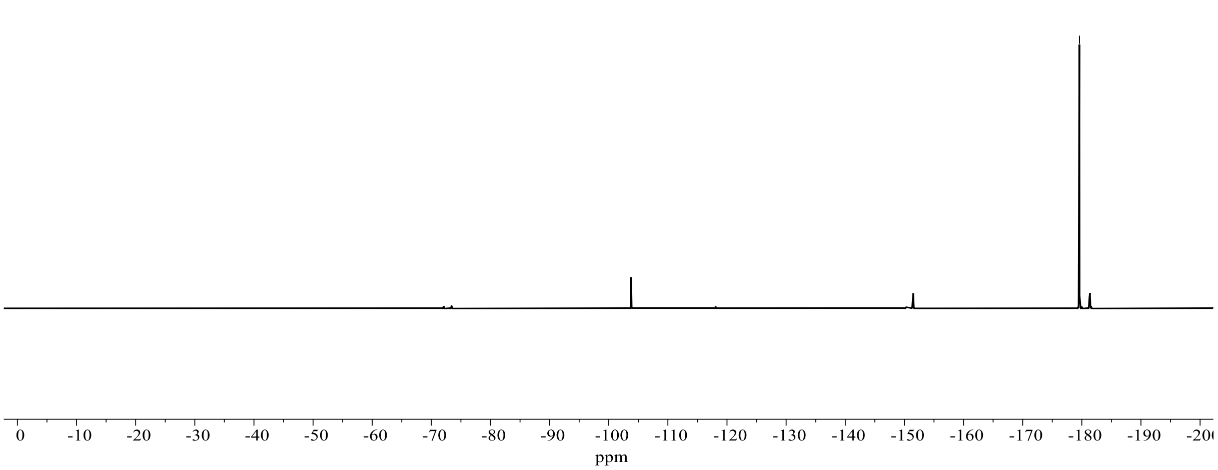

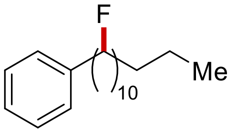

2b ^{1}H-NMR
($CDCl_3$, 400 MHz)

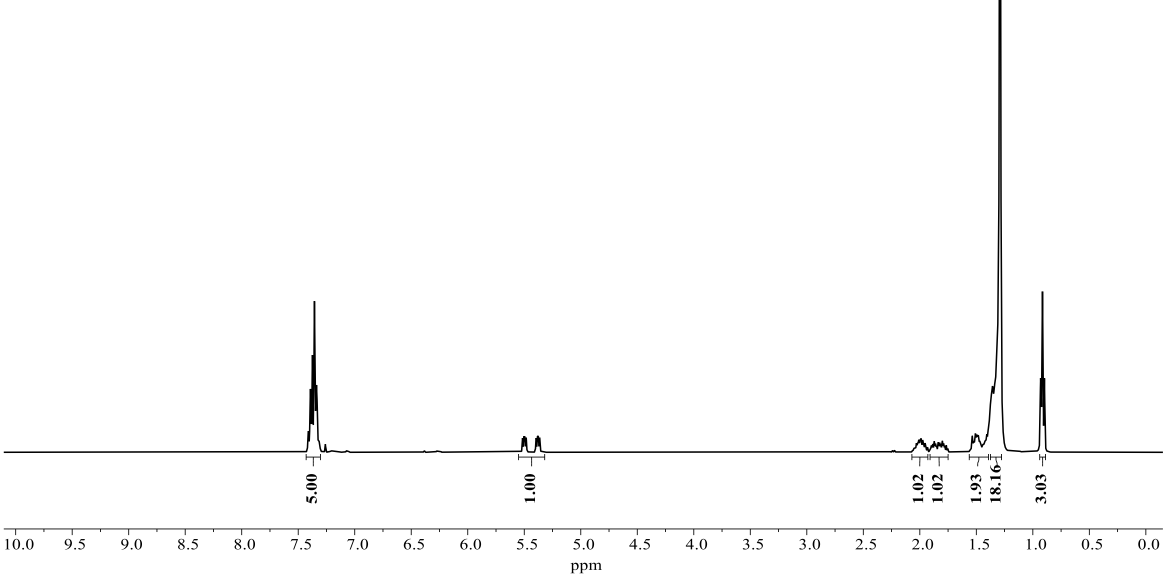

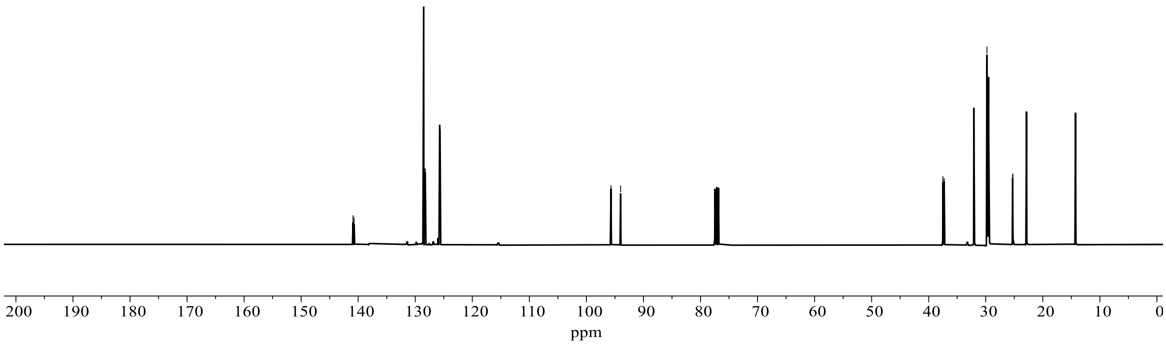

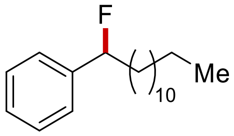

2b ^{13}C-NMR
($CDCl_3$, 101 MHz)

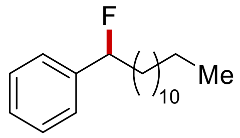

2b ^{19}F-NMR
($CDCl_3$, 282 MHz)

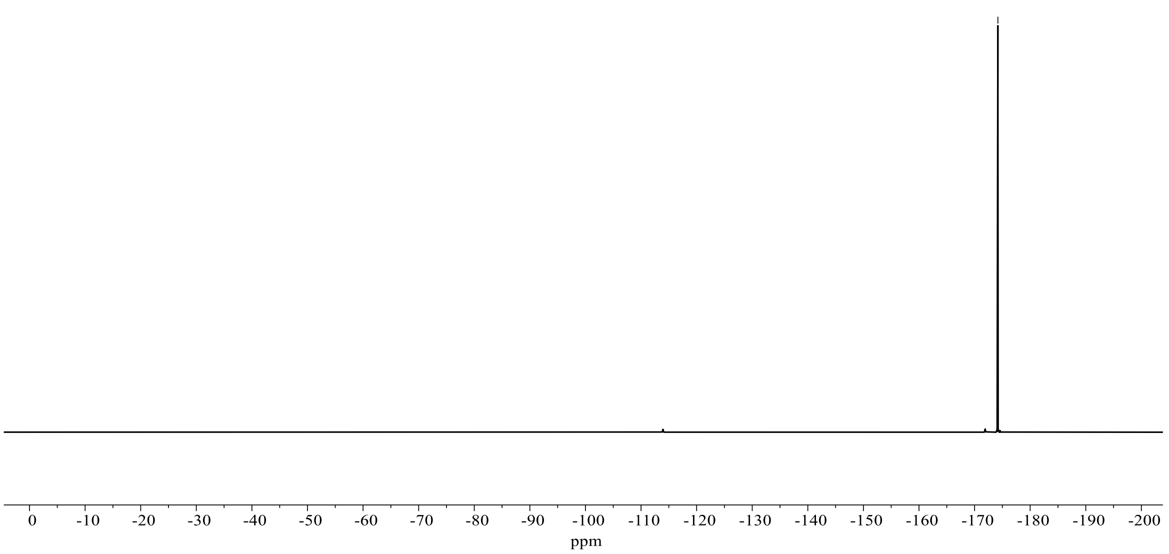

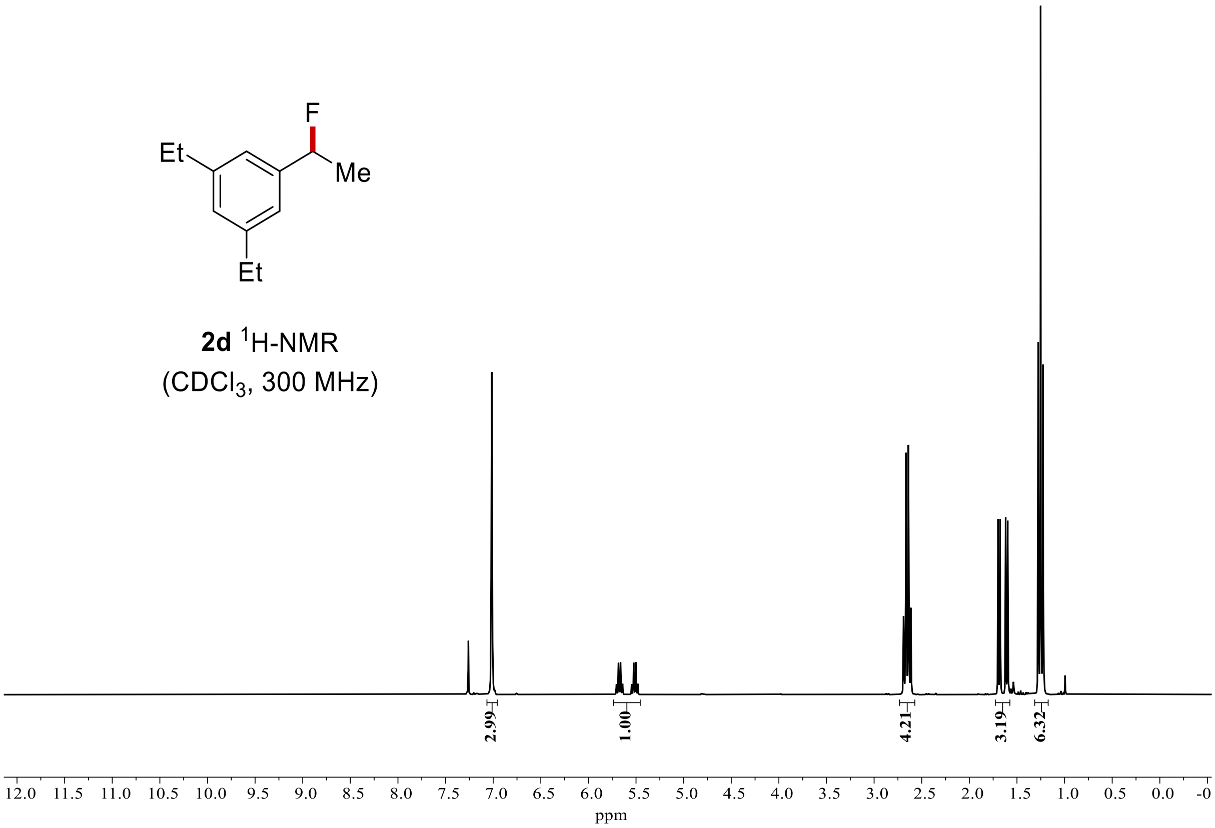
F
Et
Me
Et
2d 1H-NMR
(CDCl3, 300 MHz)
2.99
1.00
4.21
3.19
6.32
12.0 11.5 11.0 10.5 10.0 9.5 9.0 8.5 8.0 7.5 7.0 6.5 6.0 5.5 5.0 4.5 4.0 3.5 3.0 2.5 2.0 1.5 1.0 0.5 0.0 -0
ppm

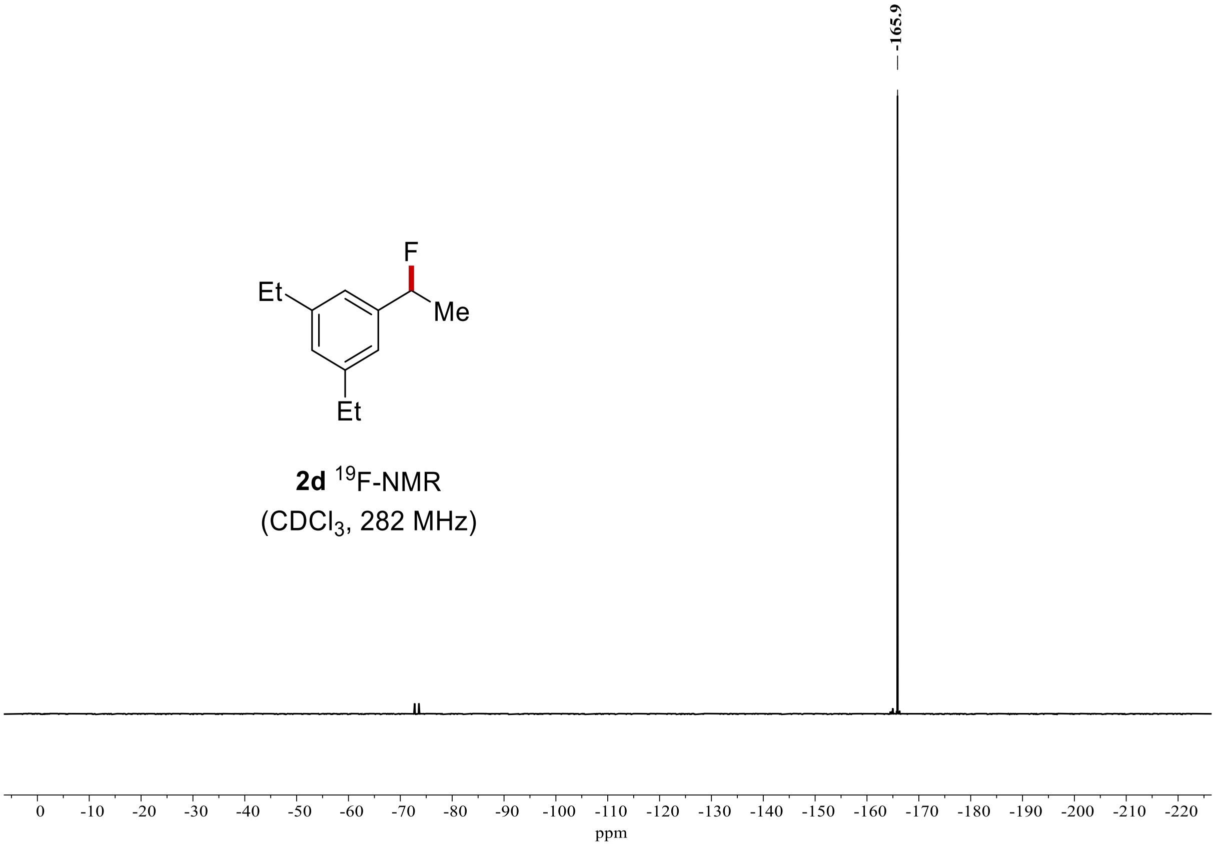
-165.9
F
Et
Me
Et
2d 19F-NMR
(CDCl3, 282 MHz)
0 -10 -20 -30 -40 -50 -60 -70 -80 -90 -100 -110 -120 -130 -140 -150 -160 -170 -180 -190 -200 -210 -220
ppm

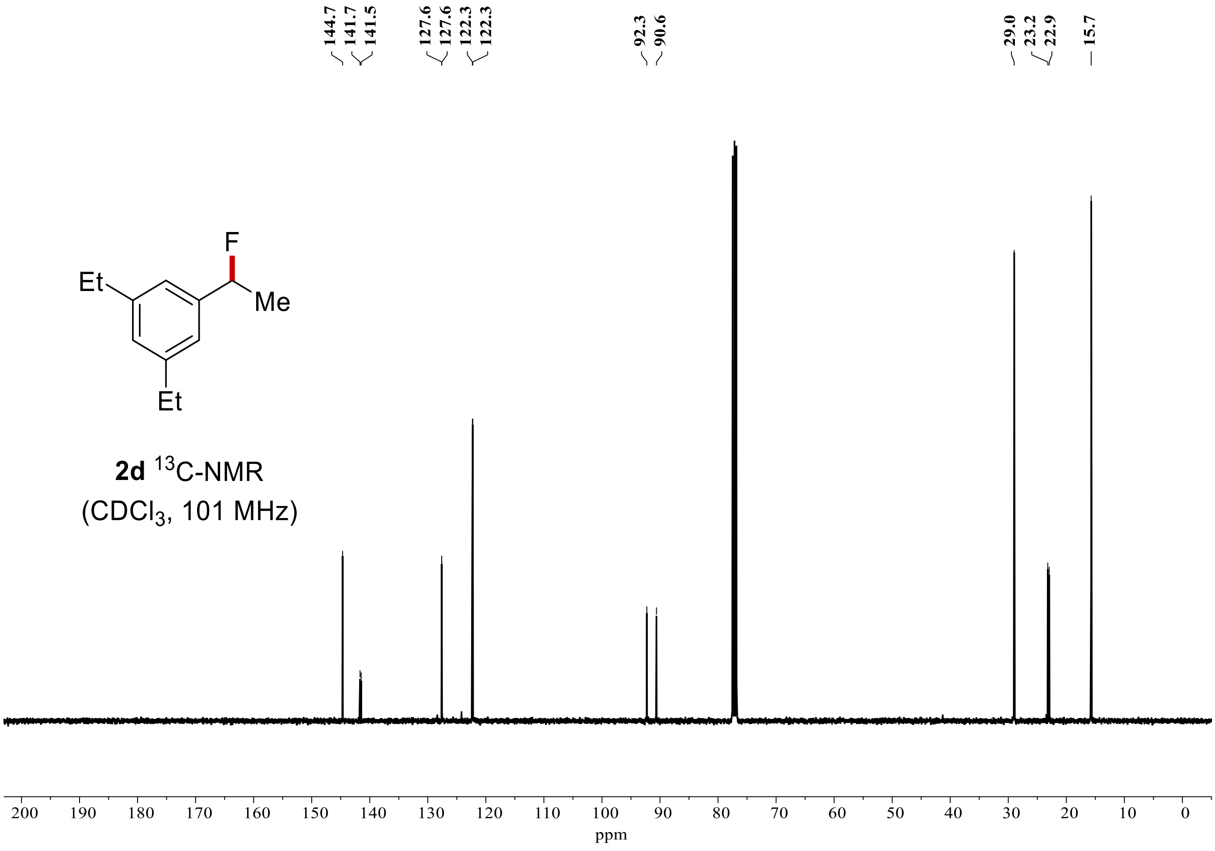

144.7
141.7
141.5
127.6
127.6
122.3
122.3
92.3
90.6
29.0
23.2
22.9
15.7
F
Et
Me
Et
2d 13C-NMR
(CDCl3, 101 MHz)
200 190 180 170 160 150 140 130 120 110 100 90 80 70 60 50 40 30 20 10 0
ppm

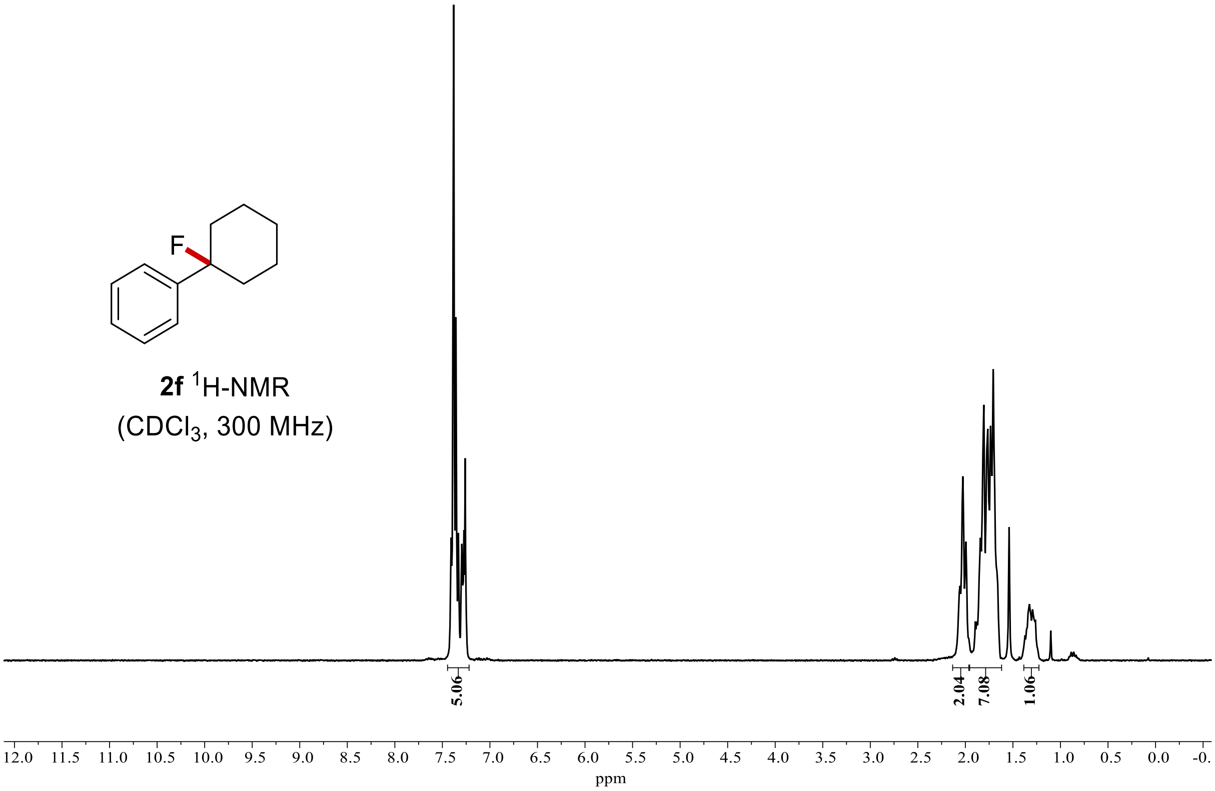

2f ^{1}H-NMR
($CDCl_3$, 300 MHz)

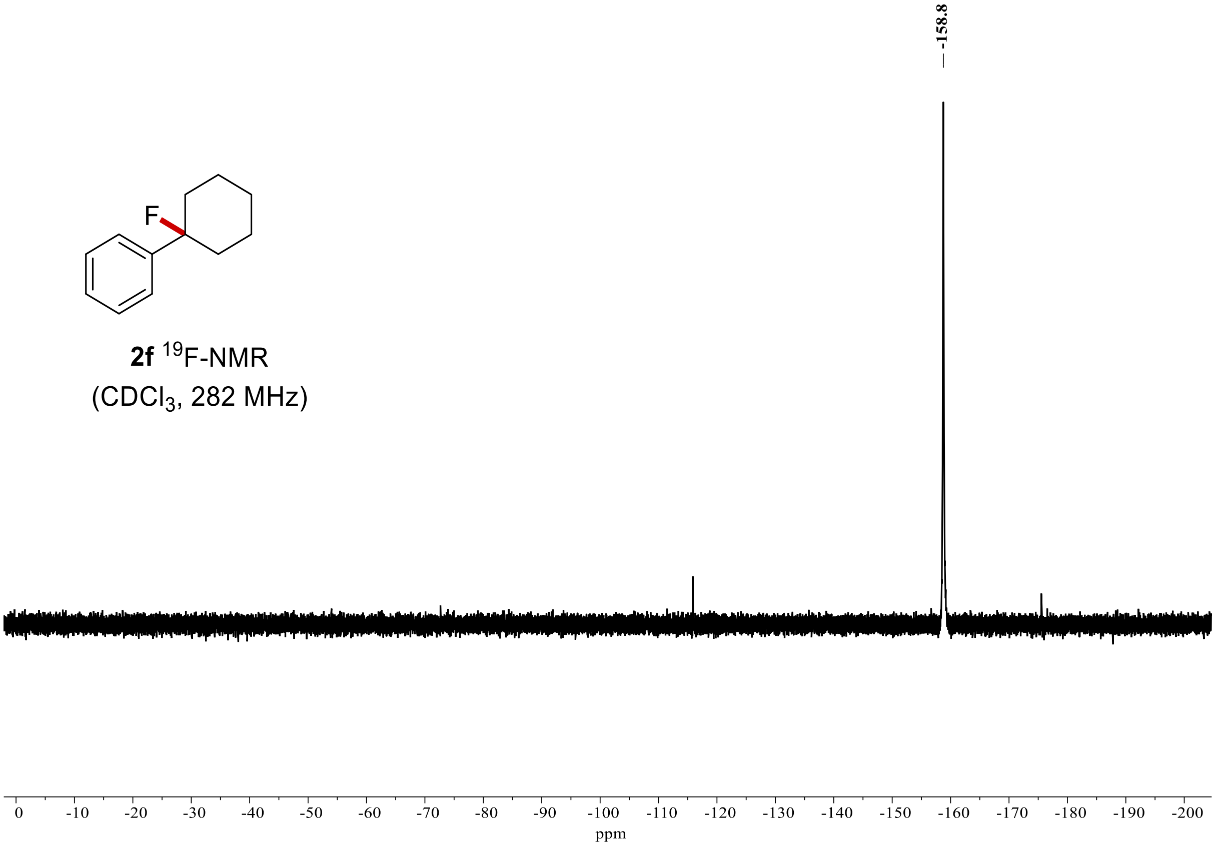

2f ^{19}F-NMR
($CDCl_3$, 282 MHz)

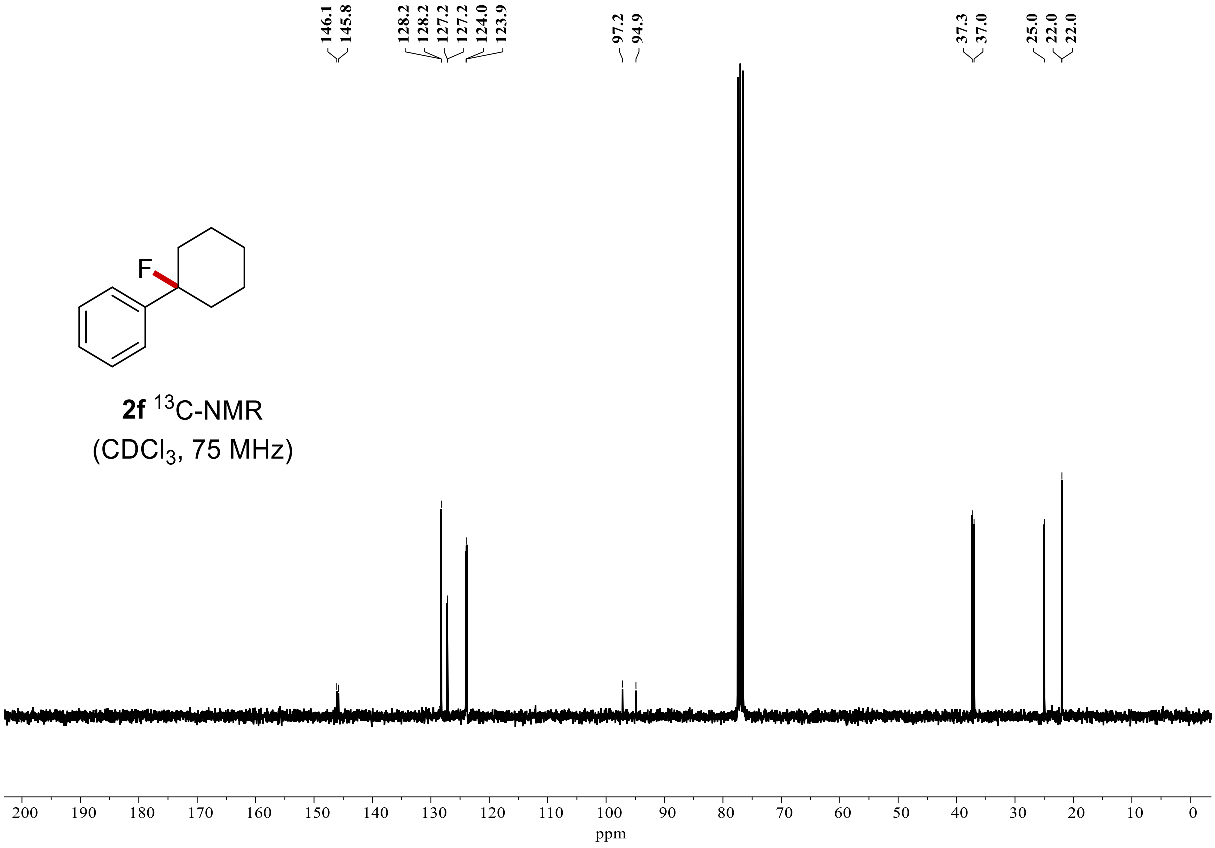
146.1
145.8
128.2
128.2
127.2
127.2
124.0
123.9
97.2
94.9
37.3
37.0
25.0
22.0
22.0
F
2f 13C-NMR
(CDCl3, 75 MHz)
200 190 180 170 160 150 140 130 120 110 100 90 80 70 60 50 40 30 20 10 0
ppm

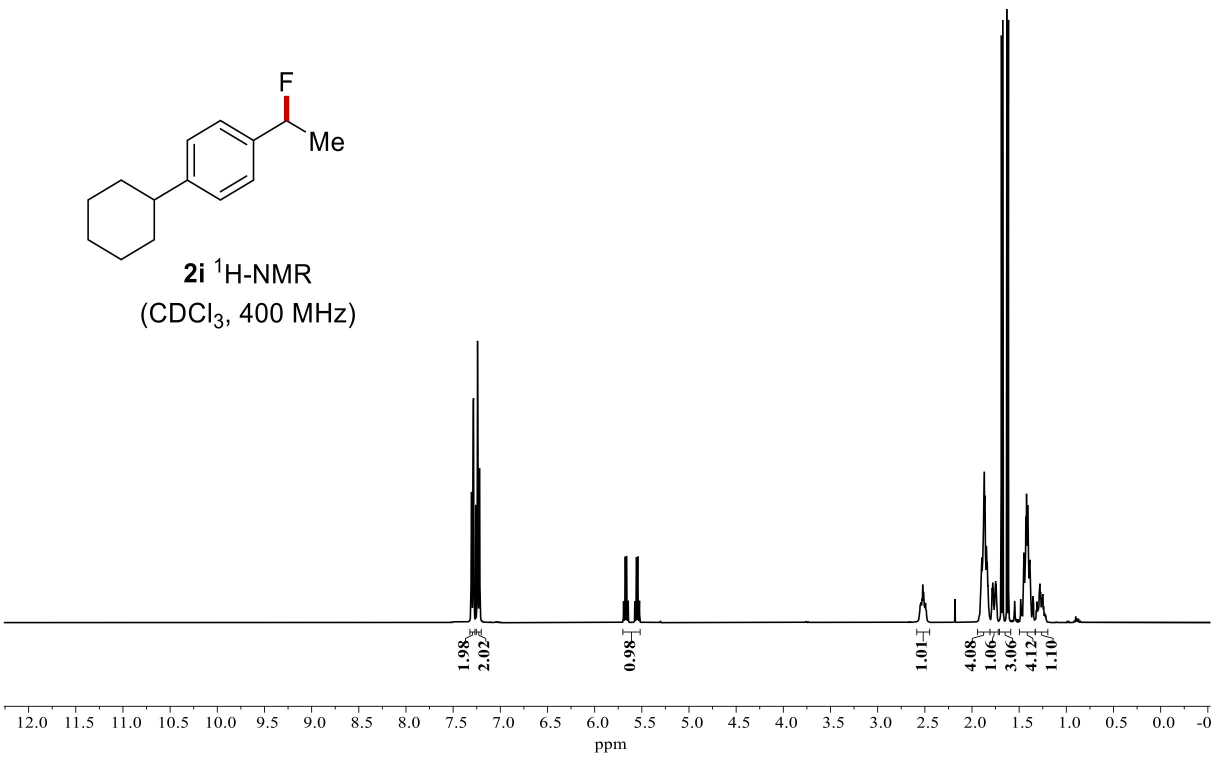
F
Me
2i ¹H-NMR
(CDCl3, 400 MHz)
1.98
2.02
0.98
1.01
4.08
1.06
3.06
4.12
1.10
12.0 11.5 11.0 10.5 10.0 9.5 9.0 8.5 8.0 7.5 7.0 6.5 6.0 5.5 5.0 4.5 4.0 3.5 3.0 2.5 2.0 1.5 1.0 0.5 0.0 -0
ppm

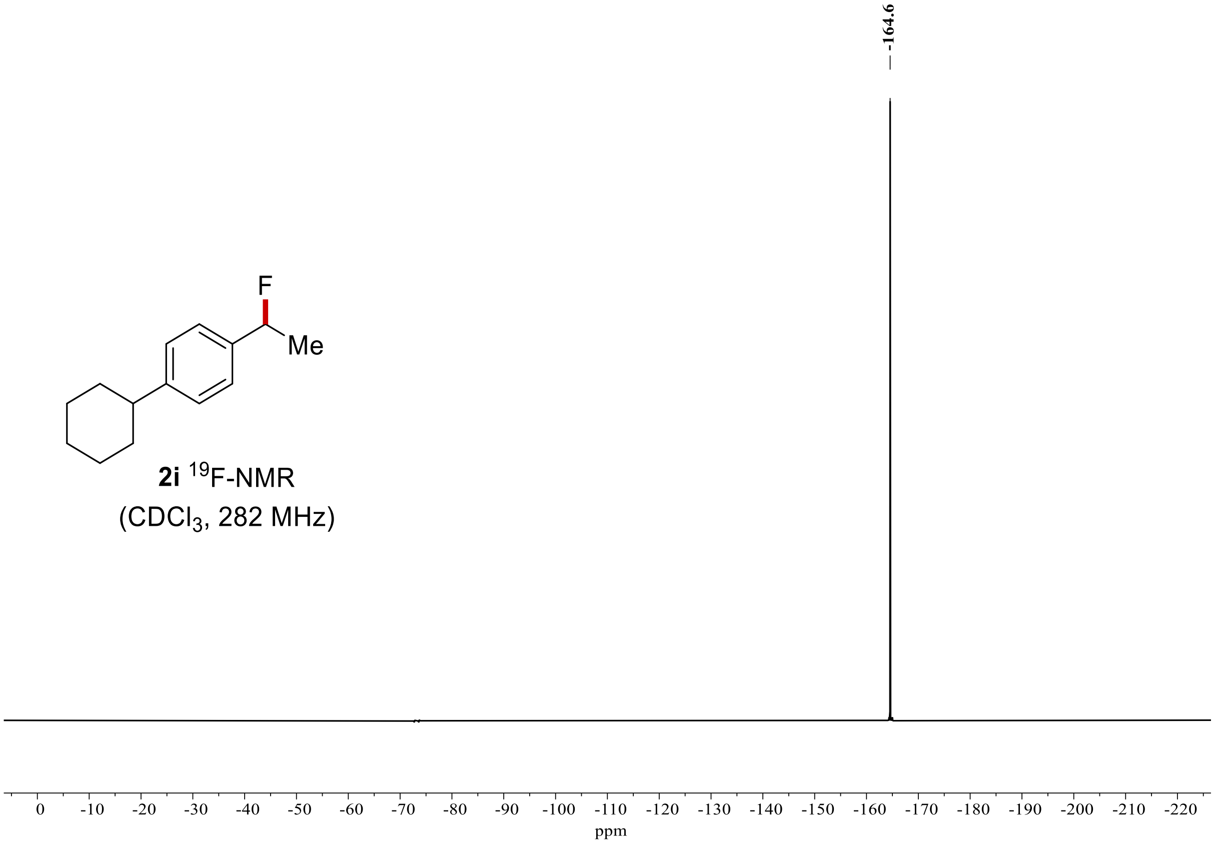
-164.6
F
Me
2i ¹⁹F-NMR
(CDCl3, 282 MHz)
0 -10 -20 -30 -40 -50 -60 -70 -80 -90 -100 -110 -120 -130 -140 -150 -160 -170 -180 -190 -200 -210 -220
ppm

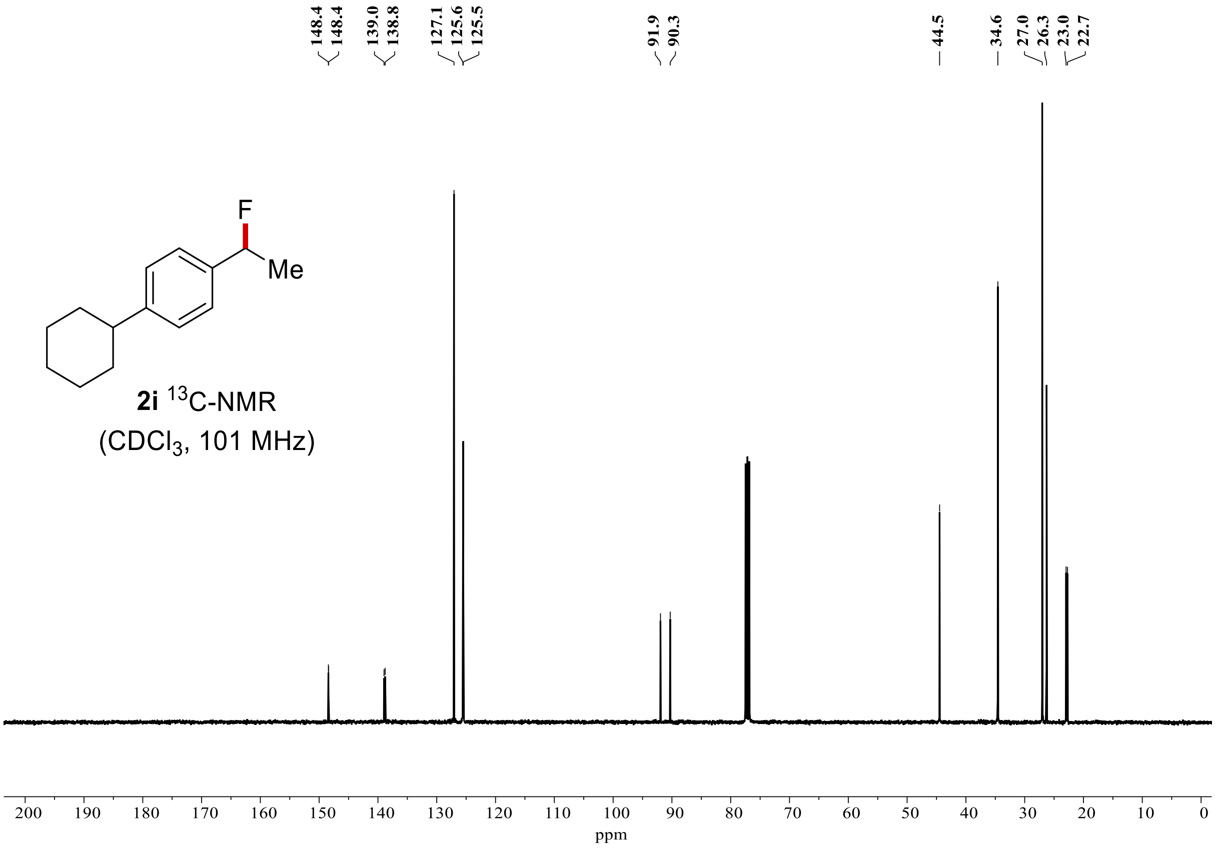
148.4
148.4
139.0
138.8
127.1
125.6
125.5
91.9
90.3
44.5
34.6
27.0
26.3
23.0
22.7
F
Me
2i 13C-NMR
(CDCl3, 101 MHz)
200 190 180 170 160 150 140 130 120 110 100 90 80 70 60 50 40 30 20 10 0
ppm

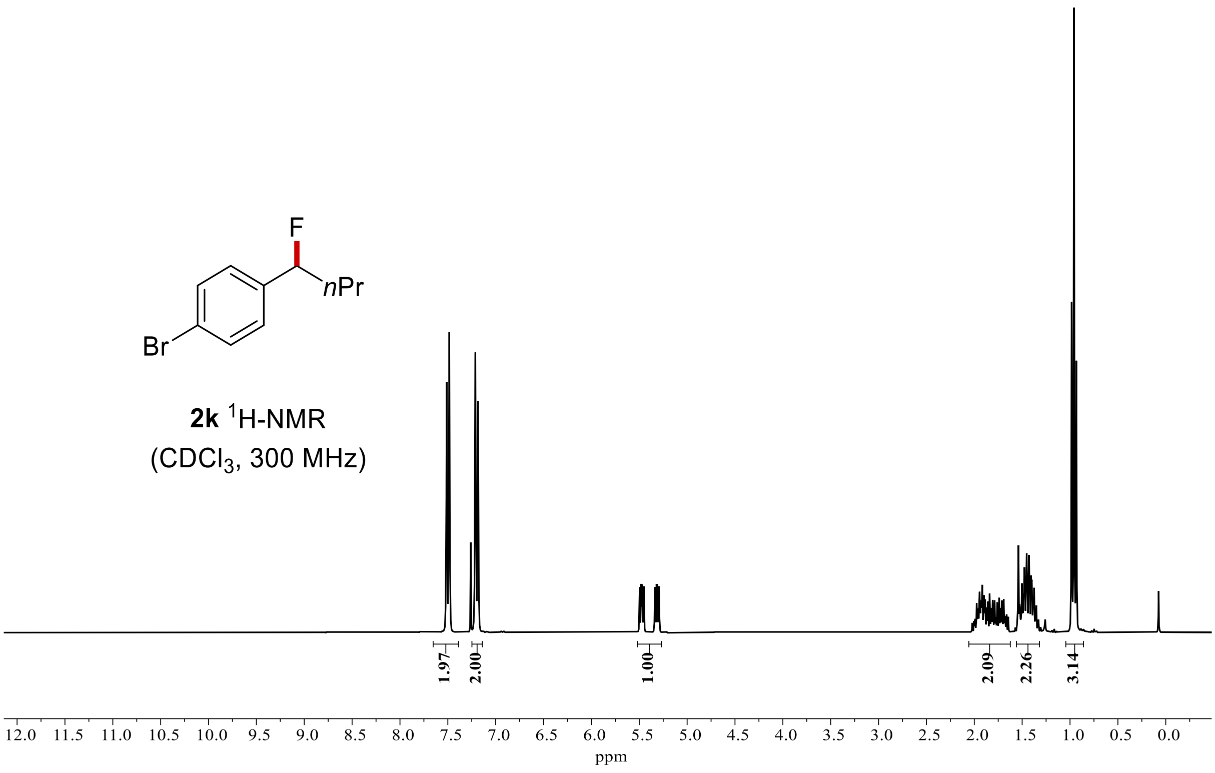

F
nPr
Br
2k ¹H-NMR
(CDCl3, 300 MHz)
1.97
2.00
1.00
2.09
2.26
3.14
12.0 11.5 11.0 10.5 10.0 9.5 9.0 8.5 8.0 7.5 7.0 6.5 6.0 5.5 5.0 4.5 4.0 3.5 3.0 2.5 2.0 1.5 1.0 0.5 0.0
ppm

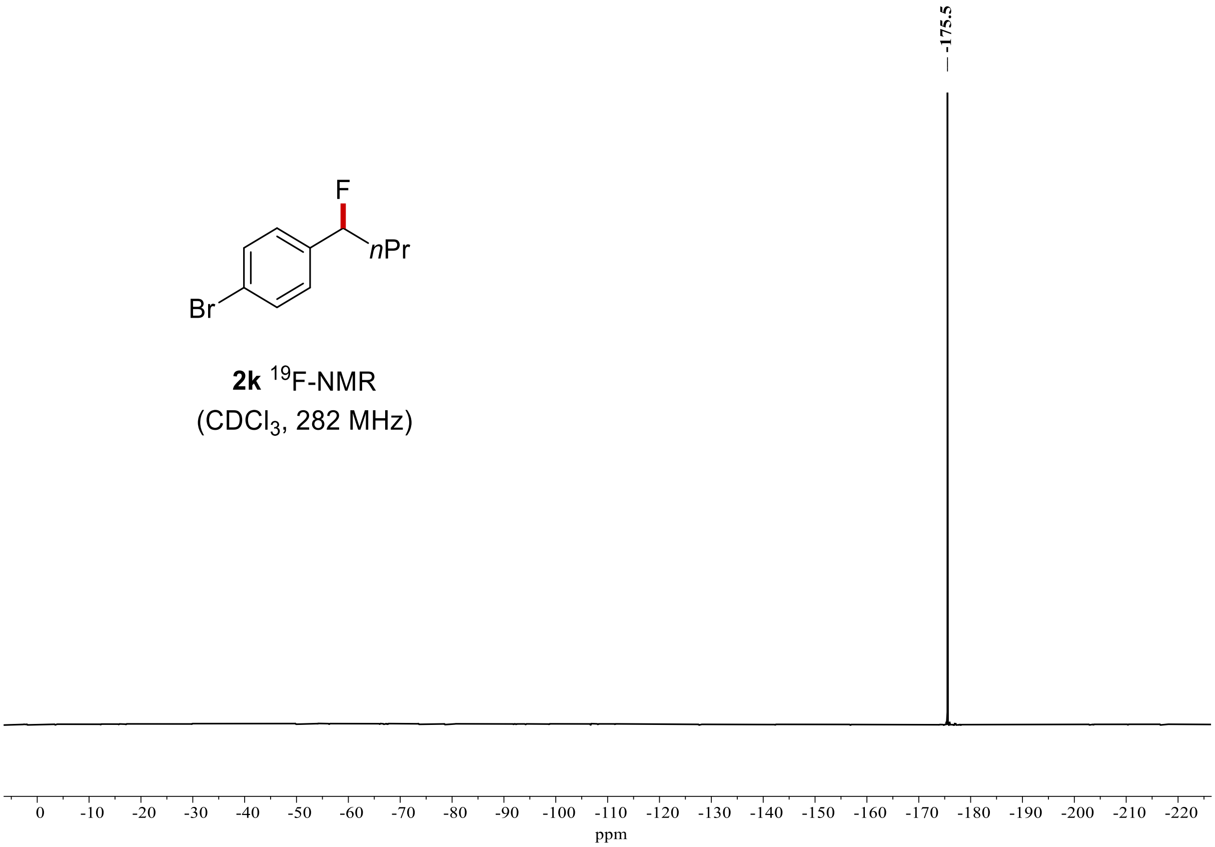

-175.5
F
nPr
Br
2k ¹⁹F-NMR
(CDCl3, 282 MHz)
0 -10 -20 -30 -40 -50 -60 -70 -80 -90 -100 -110 -120 -130 -140 -150 -160 -170 -180 -190 -200 -210 -220
ppm

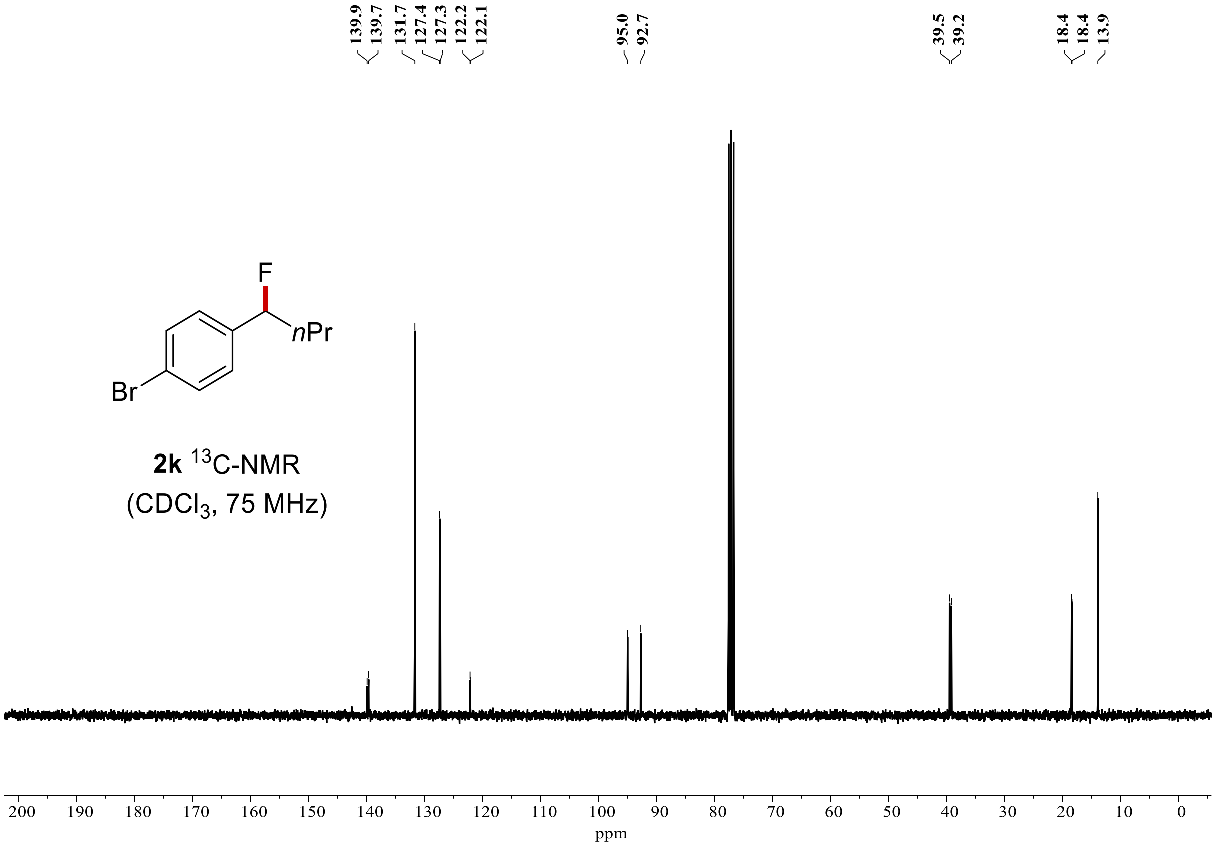

139.9
139.7
131.7
127.4
127.3
122.2
122.1
95.0
92.7
39.5
39.2
18.4
18.4
13.9
F
nPr
Br
2k 13C-NMR
(CDCl3, 75 MHz)
200 190 180 170 160 150 140 130 120 110 100 90 80 70 60 50 40 30 20 10 0
ppm

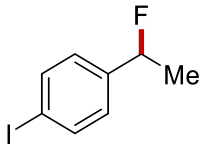

2l ^{1}H-NMR
(C_6D_6, 400 MHz)

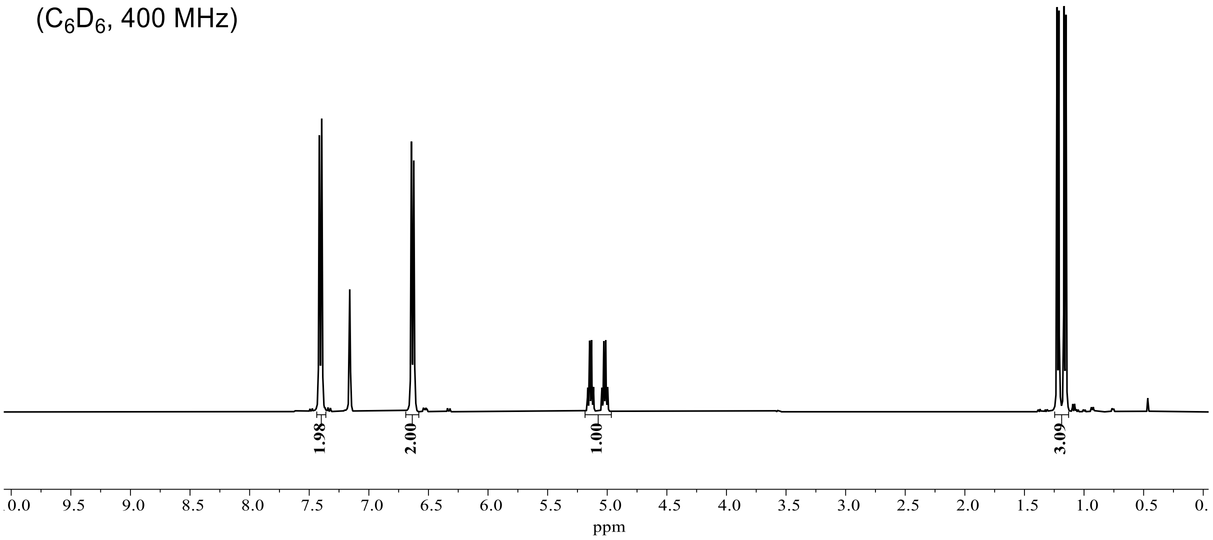

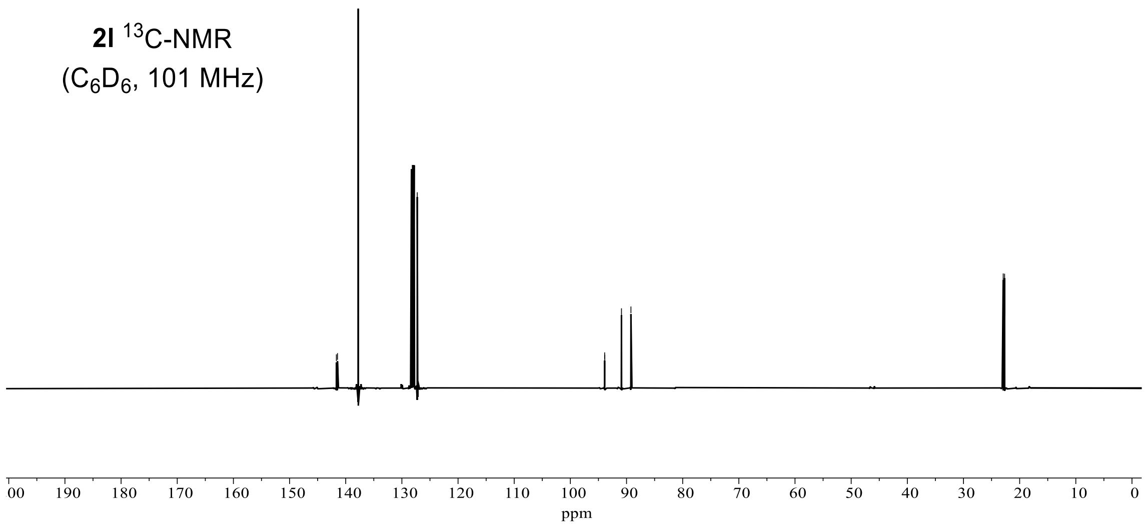

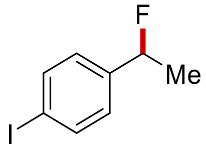

2l ^{13}C-NMR
(C_6D_6, 101 MHz)

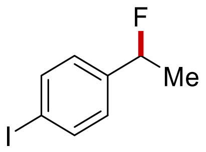

2l ^{19}F-NMR
(C_6D_6, 282 MHz)

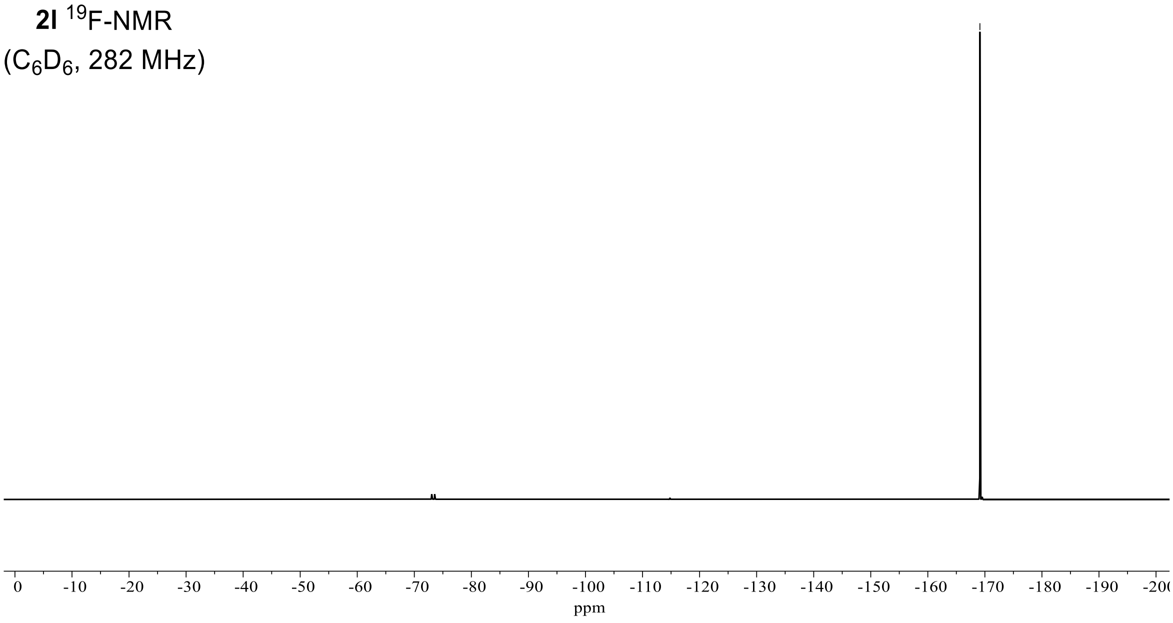

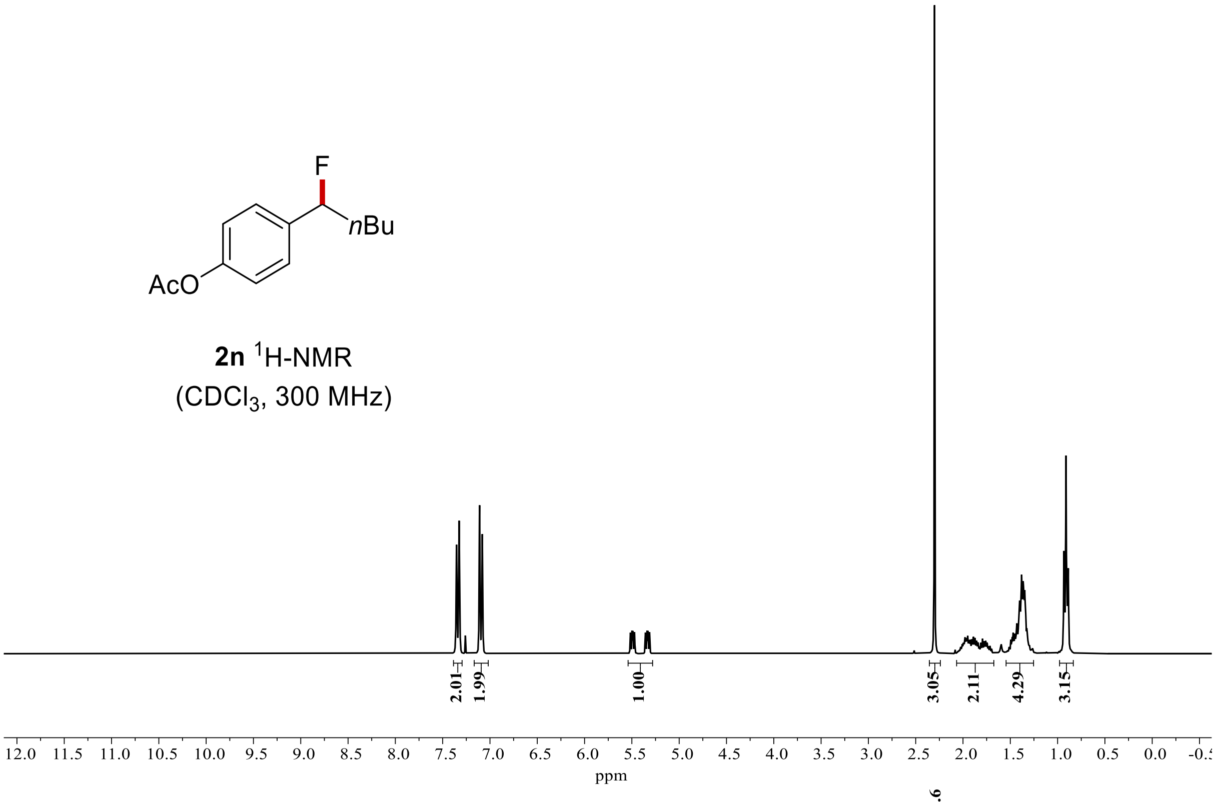
F
nBu
AcO
2n ¹H-NMR
(CDCl3, 300 MHz)
2.01
1.99
1.00
3.05
2.11
4.29
3.15
12.0 11.5 11.0 10.5 10.0 9.5 9.0 8.5 8.0 7.5 7.0 6.5 6.0 5.5 5.0 4.5 4.0 3.5 3.0 2.5 2.0 1.5 1.0 0.5 0.0
ppm

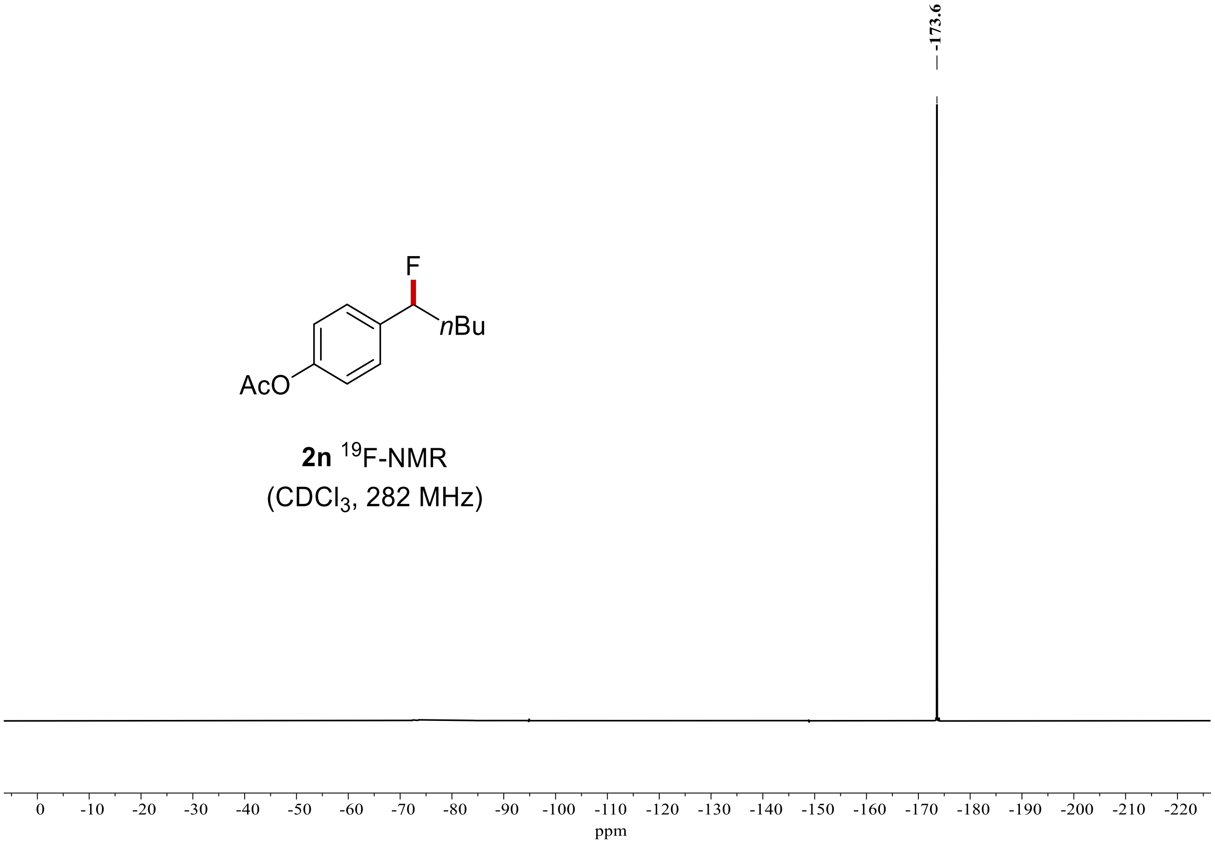
-173.6
F
nBu
AcO
2n ¹⁹F-NMR
(CDCl3, 282 MHz)
0 -10 -20 -30 -40 -50 -60 -70 -80 -90 -100 -110 -120 -130 -140 -150 -160 -170 -180 -190 -200 -210 -220
ppm

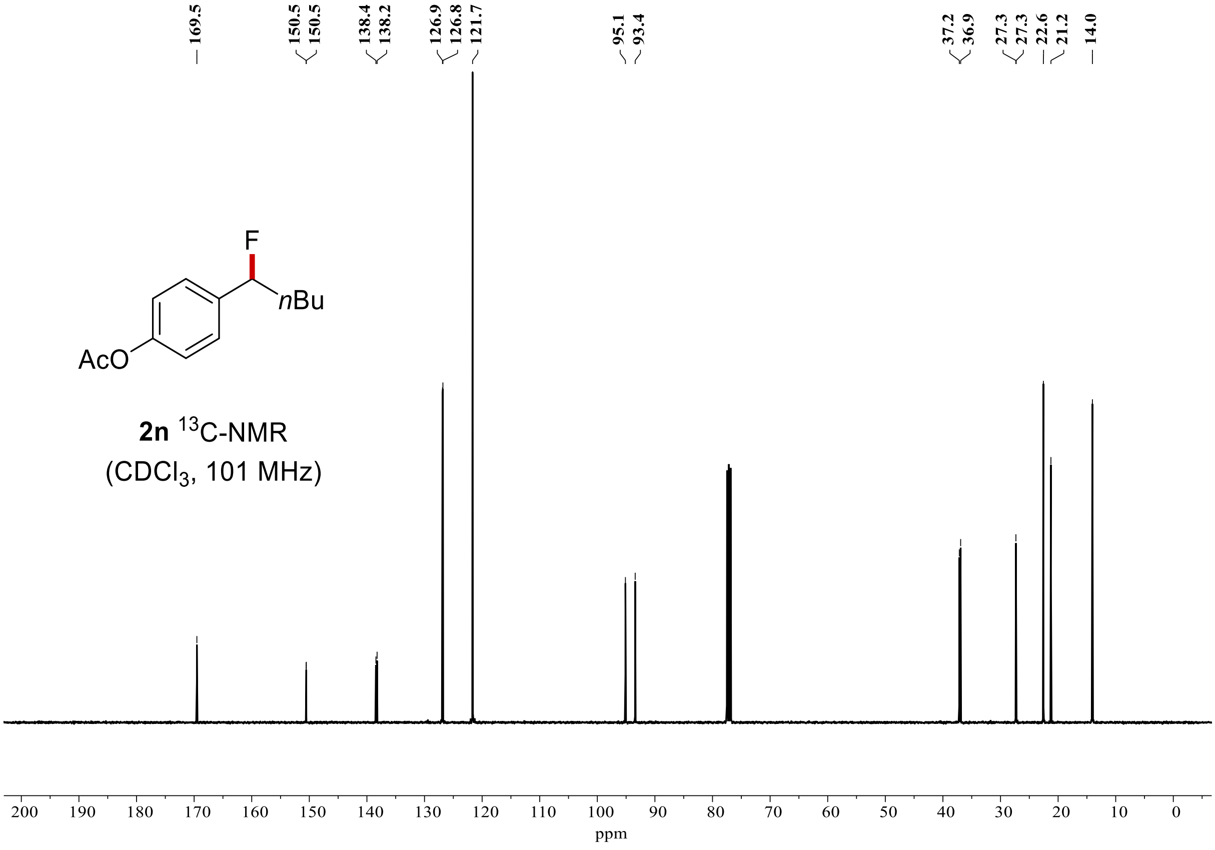
169.5
150.5
150.5
138.4
138.2
126.9
126.8
121.7
95.1
93.4
37.2
36.9
27.3
27.3
22.6
21.2
14.0
F
nBu
AcO
2n 13C-NMR
(CDCl3, 101 MHz)
200 190 180 170 160 150 140 130 120 110 100 90 80 70 60 50 40 30 20 10 0
ppm

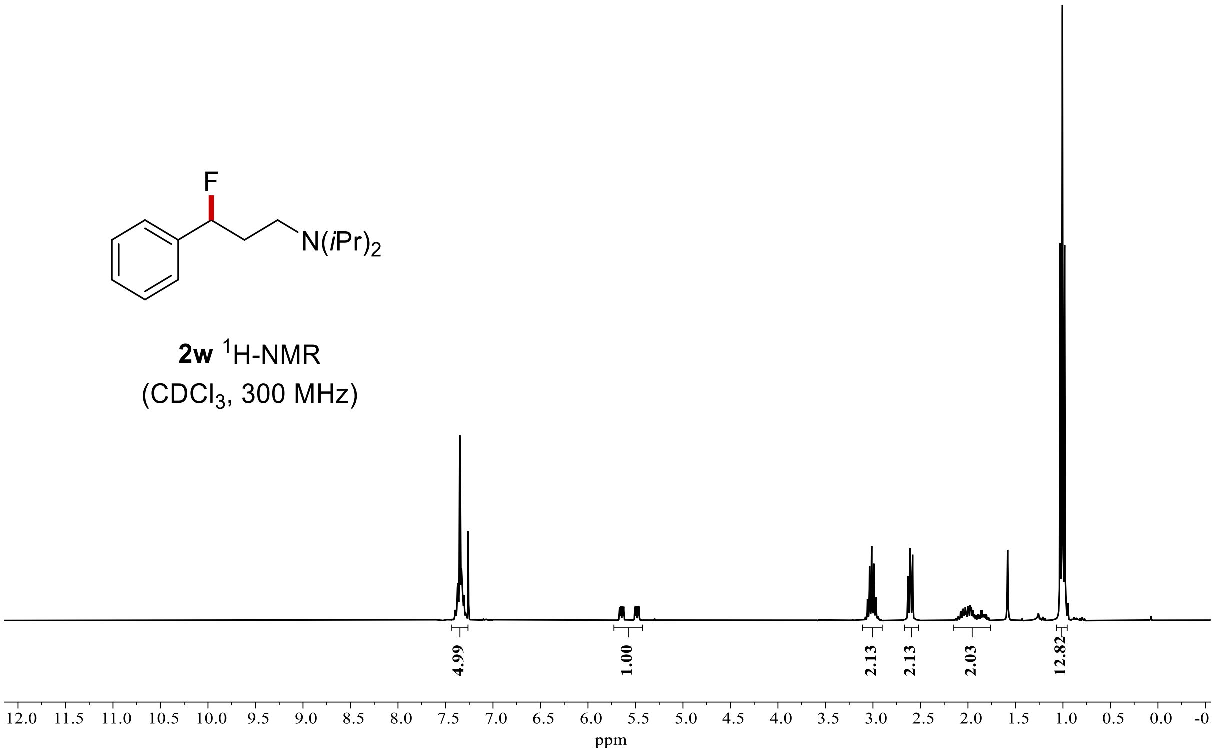
F
N(iPr)2
2w 1H-NMR
(CDCl3, 300 MHz)
4.99
1.00
2.13
2.13
2.03
12.82
12.0 11.5 11.0 10.5 10.0 9.5 9.0 8.5 8.0 7.5 7.0 6.5 6.0 5.5 5.0 4.5 4.0 3.5 3.0 2.5 2.0 1.5 1.0 0.5 0.0
ppm

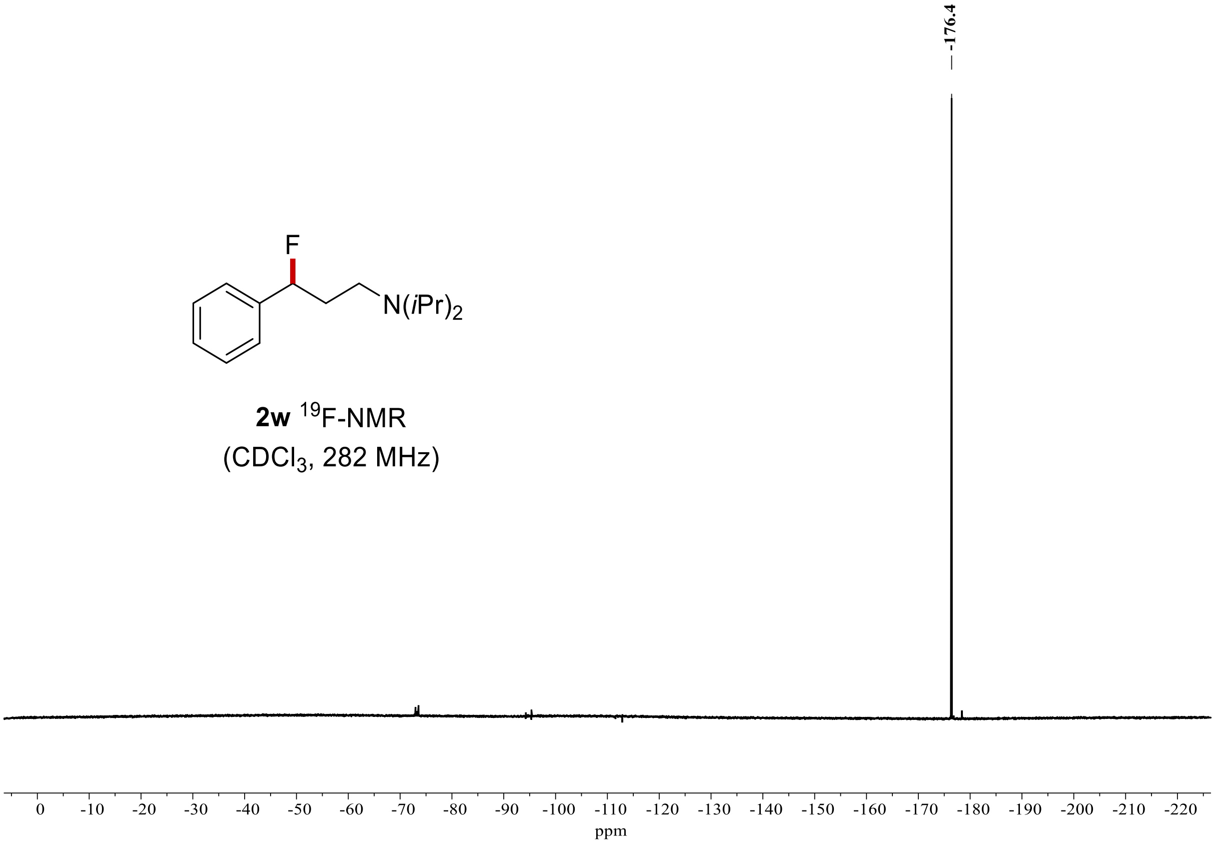
-176.4
F
N(iPr)2
2w 19F-NMR
(CDCl3, 282 MHz)
0 -10 -20 -30 -40 -50 -60 -70 -80 -90 -100 -110 -120 -130 -140 -150 -160 -170 -180 -190 -200 -210 -220
ppm

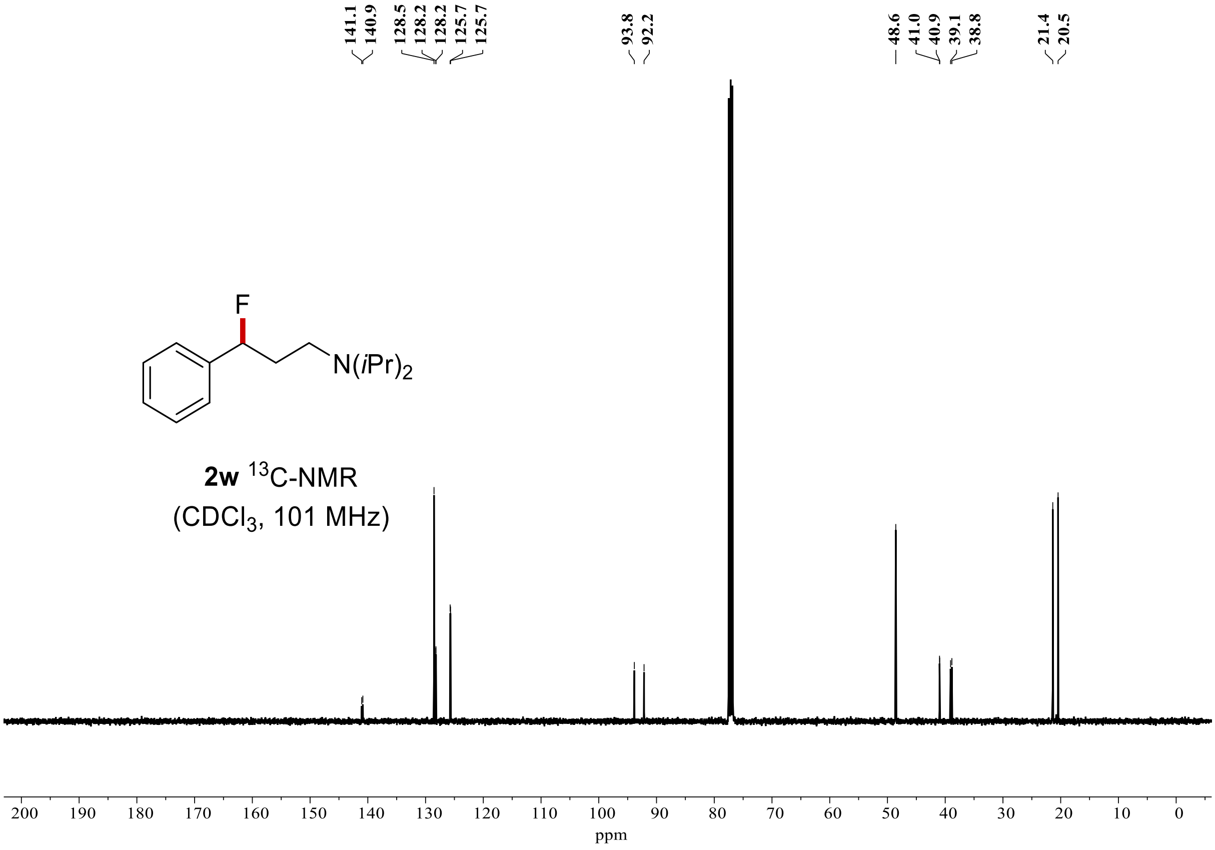
141.1
140.9
128.5
128.2
128.2
125.7
125.7
93.8
92.2
48.6
41.0
40.9
39.1
38.8
21.4
20.5
F
N(iPr)2
2w 13C-NMR
(CDCl3, 101 MHz)
200 190 180 170 160 150 140 130 120 110 100 90 80 70 60 50 40 30 20 10 0
ppm

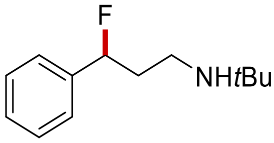

2x ^{1}H-NMR
($CDCl_3$, 400 MHz)

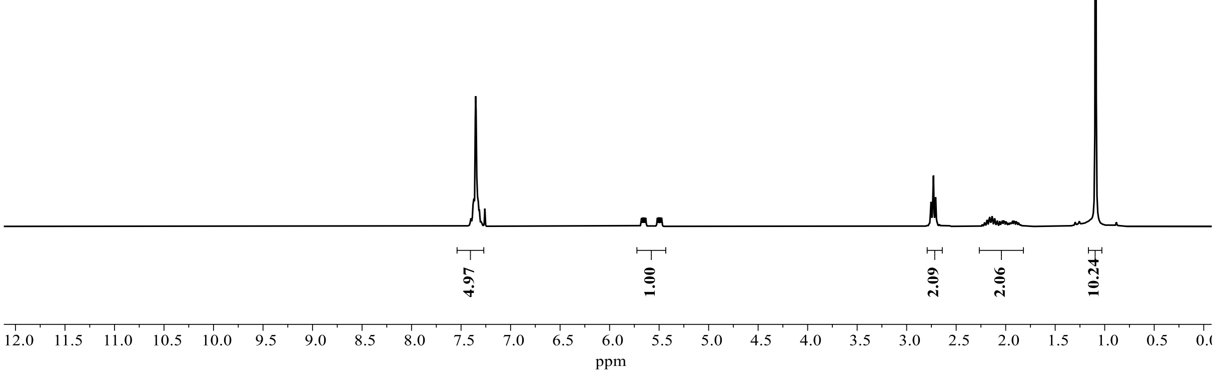

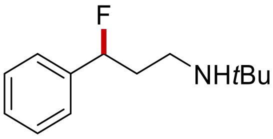

2x ^{19}F-NMR
($CDCl_3$, 282 MHz)

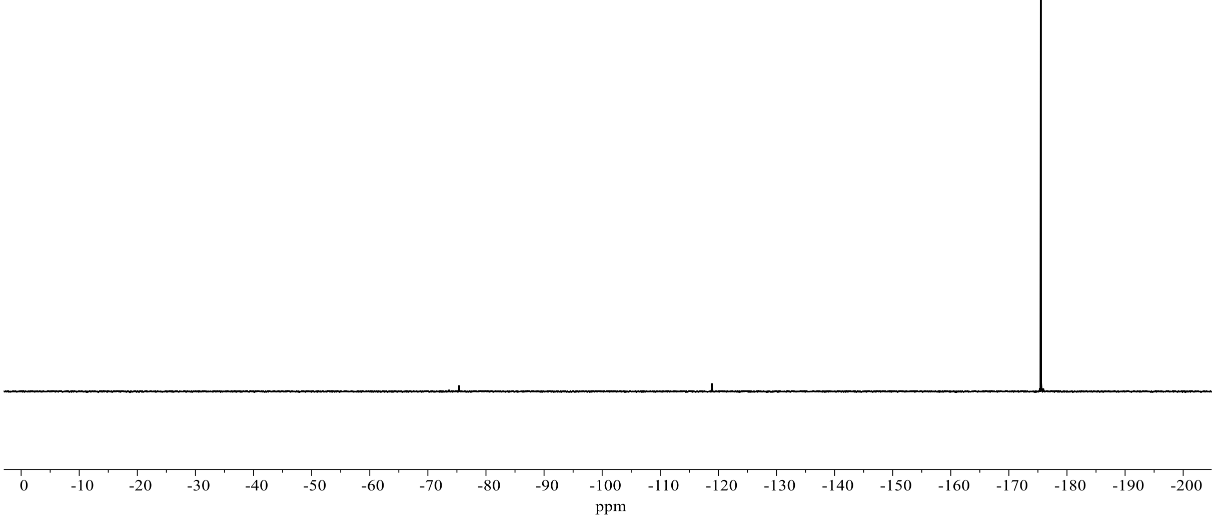

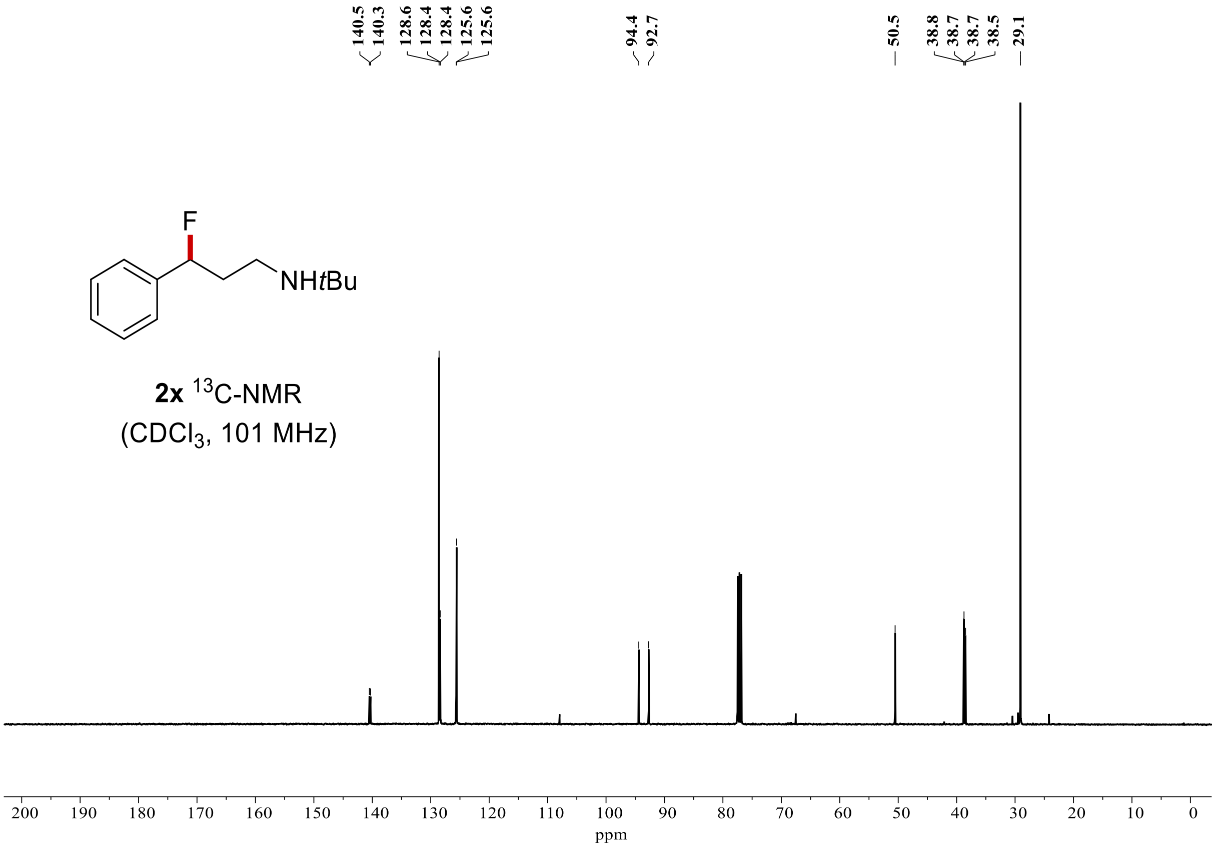
140.5
140.3
128.6
128.4
128.4
125.6
125.6
94.4
92.7
50.5
38.8
38.7
38.7
38.5
29.1
F
NHtBu
2x 13C-NMR
(CDCl3, 101 MHz)
200 190 180 170 160 150 140 130 120 110 100 90 80 70 60 50 40 30 20 10 0
ppm

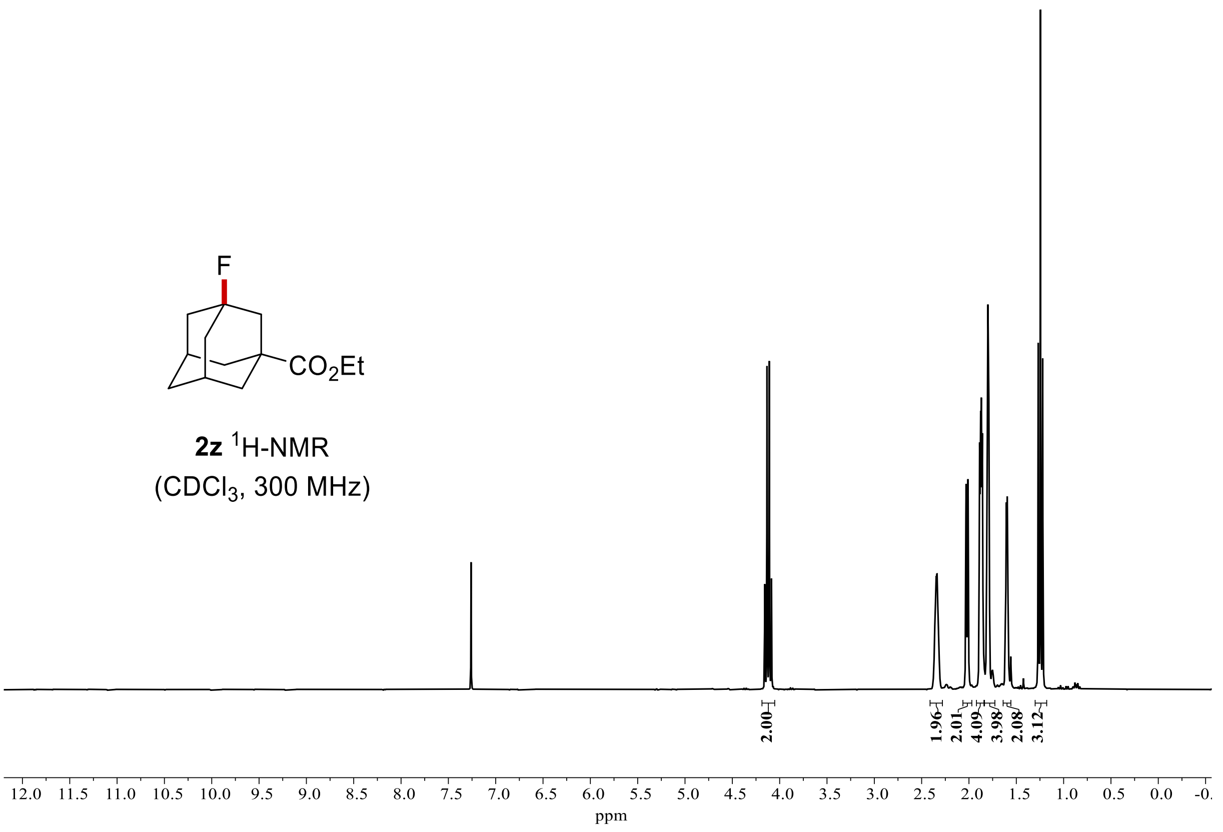

F
CO2Et
2z 1H-NMR
(CDCl3, 300 MHz)
2.00
1.96
2.01
4.09
3.98
2.08
3.12
12.0 11.5 11.0 10.5 10.0 9.5 9.0 8.5 8.0 7.5 7.0 6.5 6.0 5.5 5.0 4.5 4.0 3.5 3.0 2.5 2.0 1.5 1.0 0.5 0.0
ppm

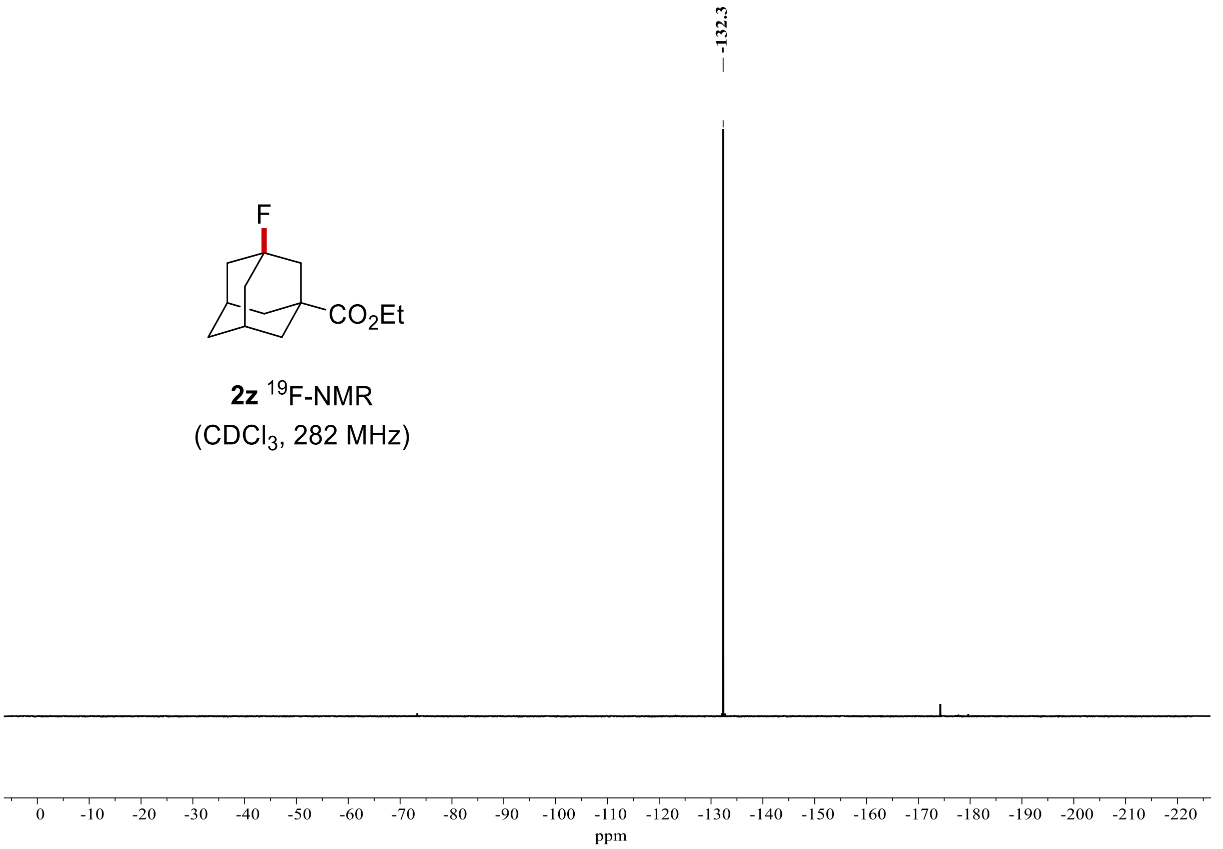

-132.3
F
CO2Et
2z 19F-NMR
(CDCl3, 282 MHz)
0 -10 -20 -30 -40 -50 -60 -70 -80 -90 -100 -110 -120 -130 -140 -150 -160 -170 -180 -190 -200 -210 -220
ppm

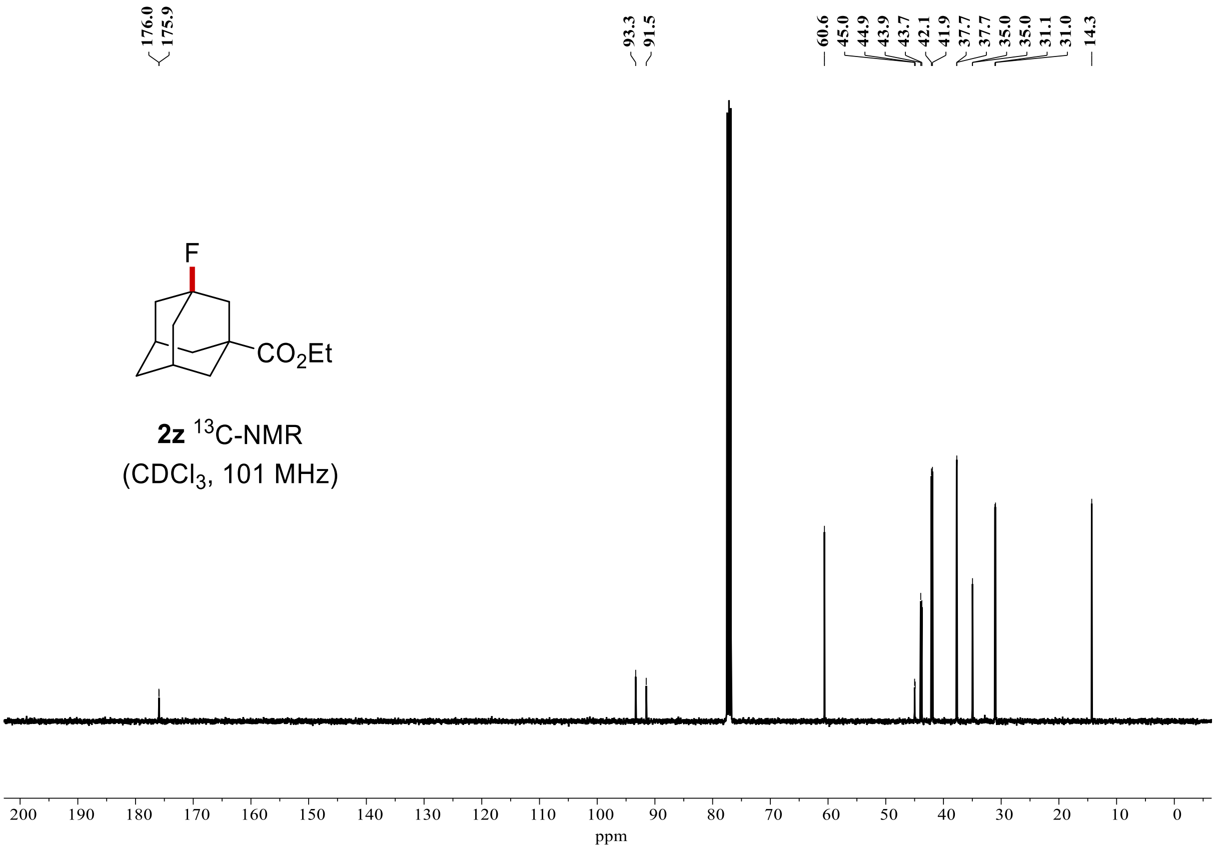
176.0
175.9
93.3
91.5
60.6
45.0
44.9
43.9
43.7
42.1
41.9
37.7
37.7
35.0
35.0
31.1
31.0
14.3
F
CO2Et
2z 13C-NMR
(CDCl3, 101 MHz)
200 190 180 170 160 150 140 130 120 110 100 90 80 70 60 50 40 30 20 10 0
ppm

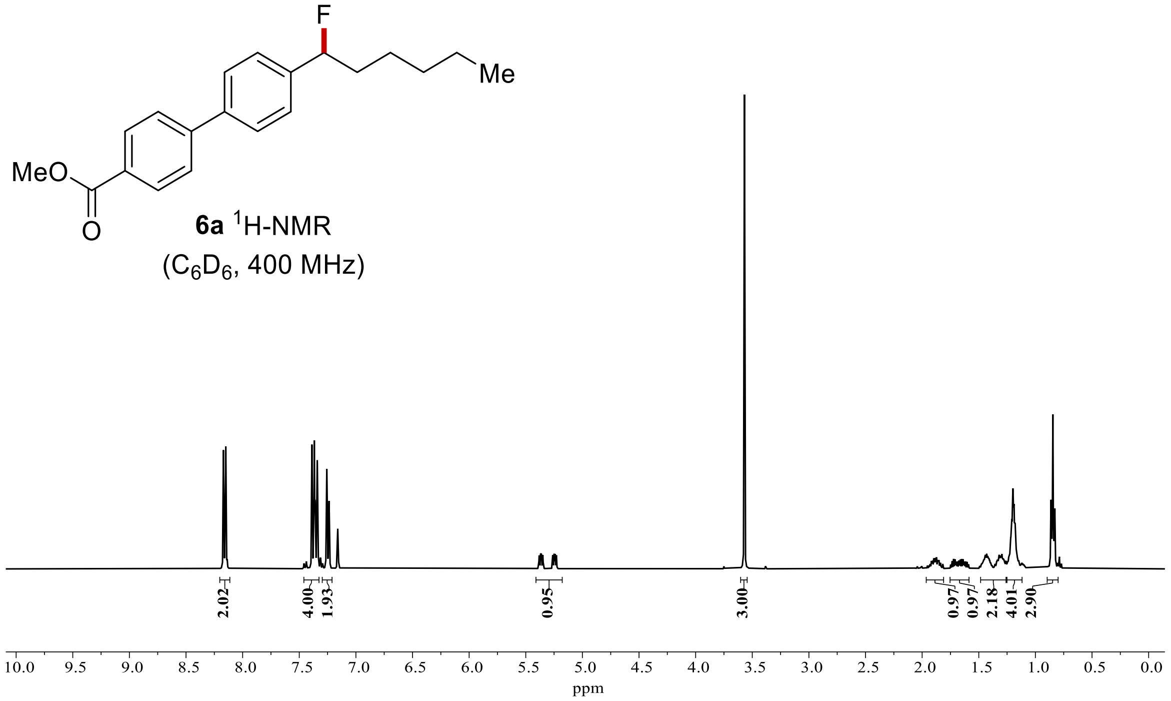
F
Me
MeO
O
6a 1H-NMR
(C6D6, 400 MHz)
2.02
4.00
1.93
0.95
3.00
0.97
0.97
2.18
4.01
2.90
10.0 9.5 9.0 8.5 8.0 7.5 7.0 6.5 6.0 5.5 5.0 4.5 4.0 3.5 3.0 2.5 2.0 1.5 1.0 0.5 0.0
ppm

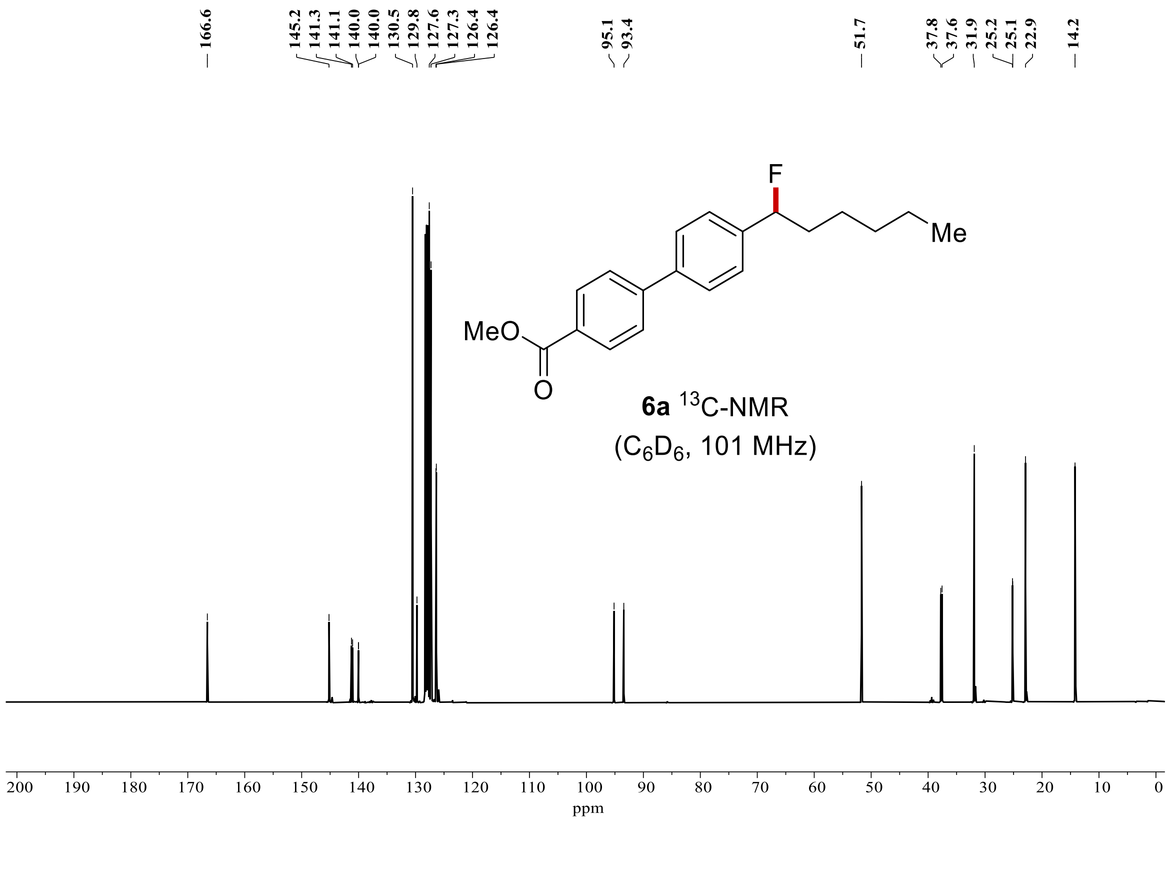
166.6
145.2
141.3
141.1
140.0
140.0
130.5
129.8
127.6
127.3
126.4
126.4
95.1
93.4
51.7
37.8
37.6
31.9
25.2
25.1
22.9
14.2
F
Me
MeO
O
6a 13C-NMR
(C6D6, 101 MHz)
200 190 180 170 160 150 140 130 120 110 100 90 80 70 60 50 40 30 20 10 0
ppm

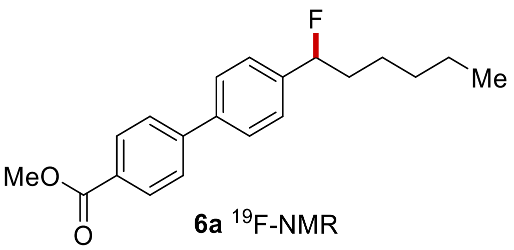

6a ^{19}F-NMR
(C_6D_6, 282 MHz)

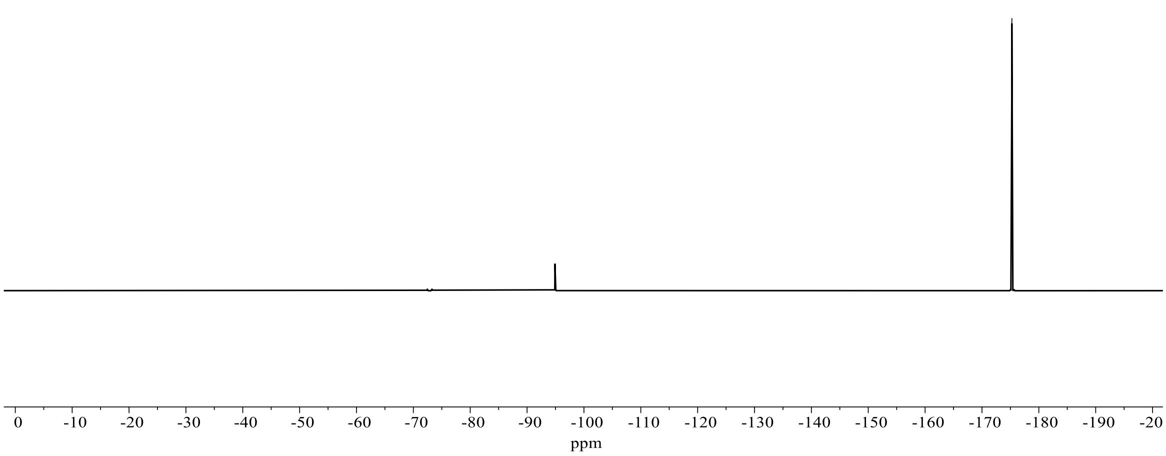

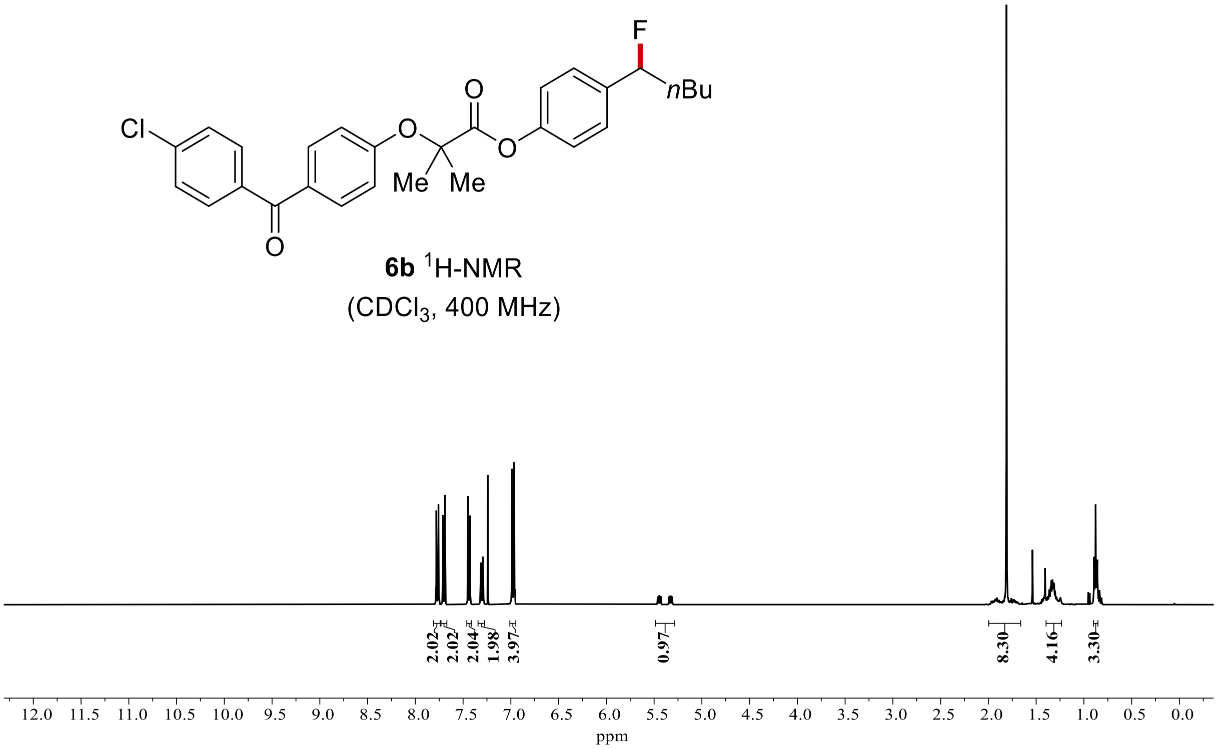
6b ¹H-NMR
(CDCl3, 400 MHz)
2.02
2.02
2.04
1.98
3.97
0.97
8.30
4.16
3.30
12.0 11.5 11.0 10.5 10.0 9.5 9.0 8.5 8.0 7.5 7.0 6.5 6.0 5.5 5.0 4.5 4.0 3.5 3.0 2.5 2.0 1.5 1.0 0.5 0.0
ppm

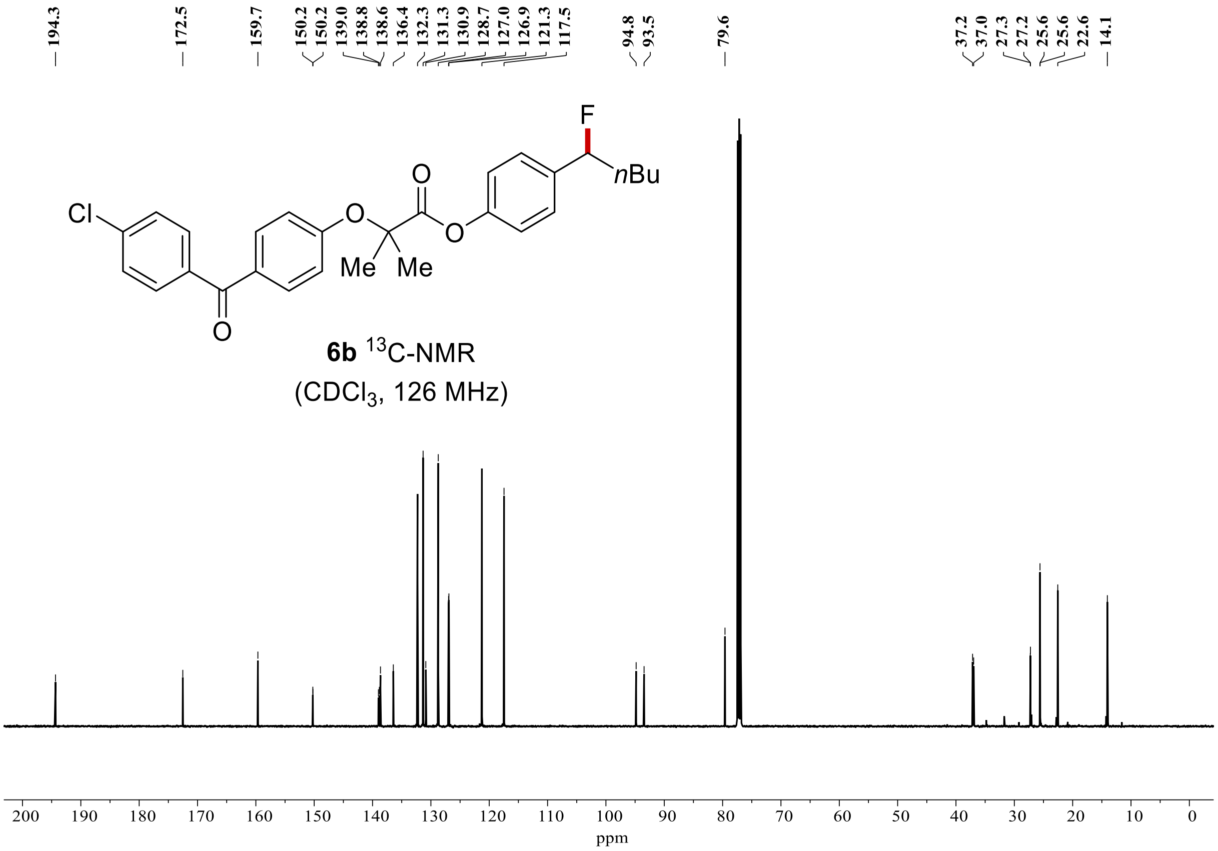
194.3
172.5
159.7
150.2
150.2
139.0
138.8
138.6
136.4
132.3
131.3
130.9
128.7
127.0
126.9
121.3
117.5
94.8
93.5
79.6
37.2
37.0
27.3
27.2
25.6
25.6
22.6
14.1
6b ¹³C-NMR
(CDCl3, 126 MHz)
200 190 180 170 160 150 140 130 120 110 100 90 80 70 60 50 40 30 20 10 0
ppm

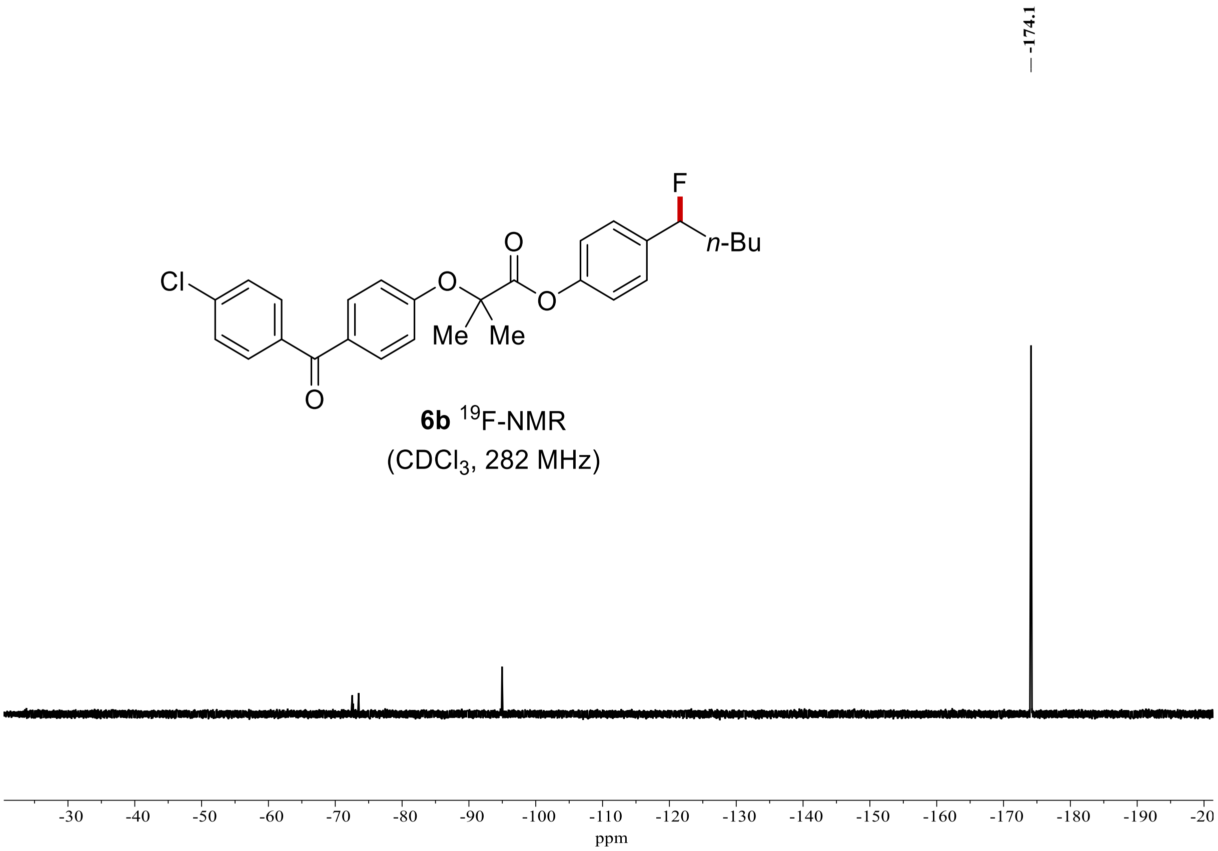

-174.1
F
O
n-Bu
Cl
O
O
Me
Me
O
6b ¹⁹F-NMR
(CDCl₃, 282 MHz)
ppm

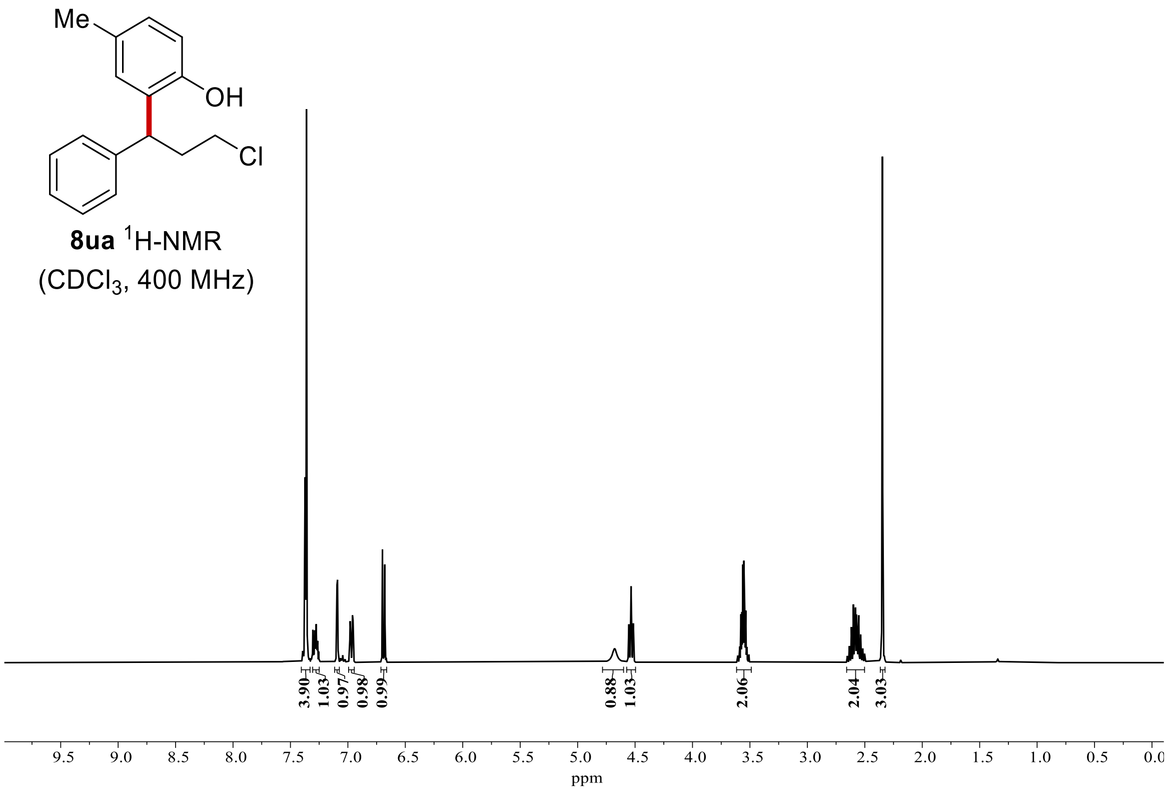

Me
OH
Cl
8ua ¹H-NMR
(CDCl₃, 400 MHz)
3.90
1.03
0.97
0.98
0.99
0.88
1.03
2.06
2.04
3.03
ppm

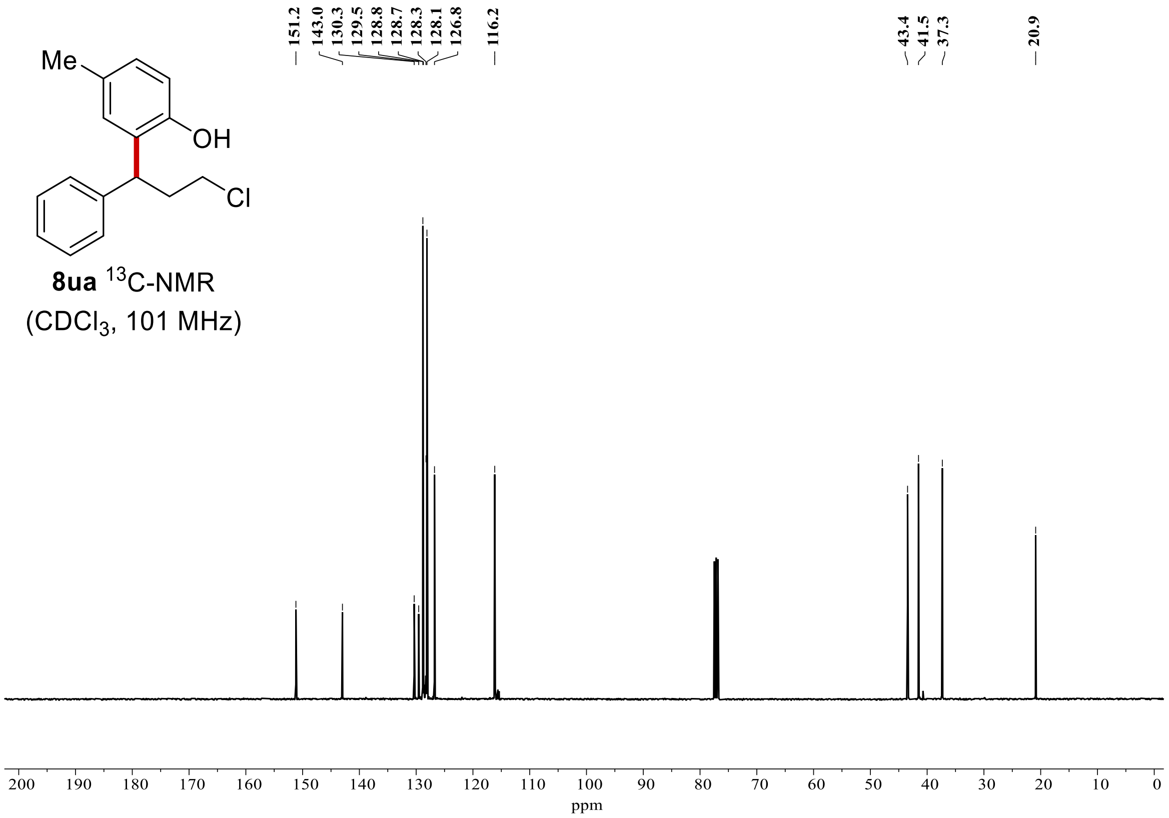
Me
OH
Cl
8ua 13C-NMR
(CDCl3, 101 MHz)
151.2
143.0
130.3
129.5
128.8
128.7
128.3
128.1
126.8
116.2
43.4
41.5
37.3
20.9
ppm

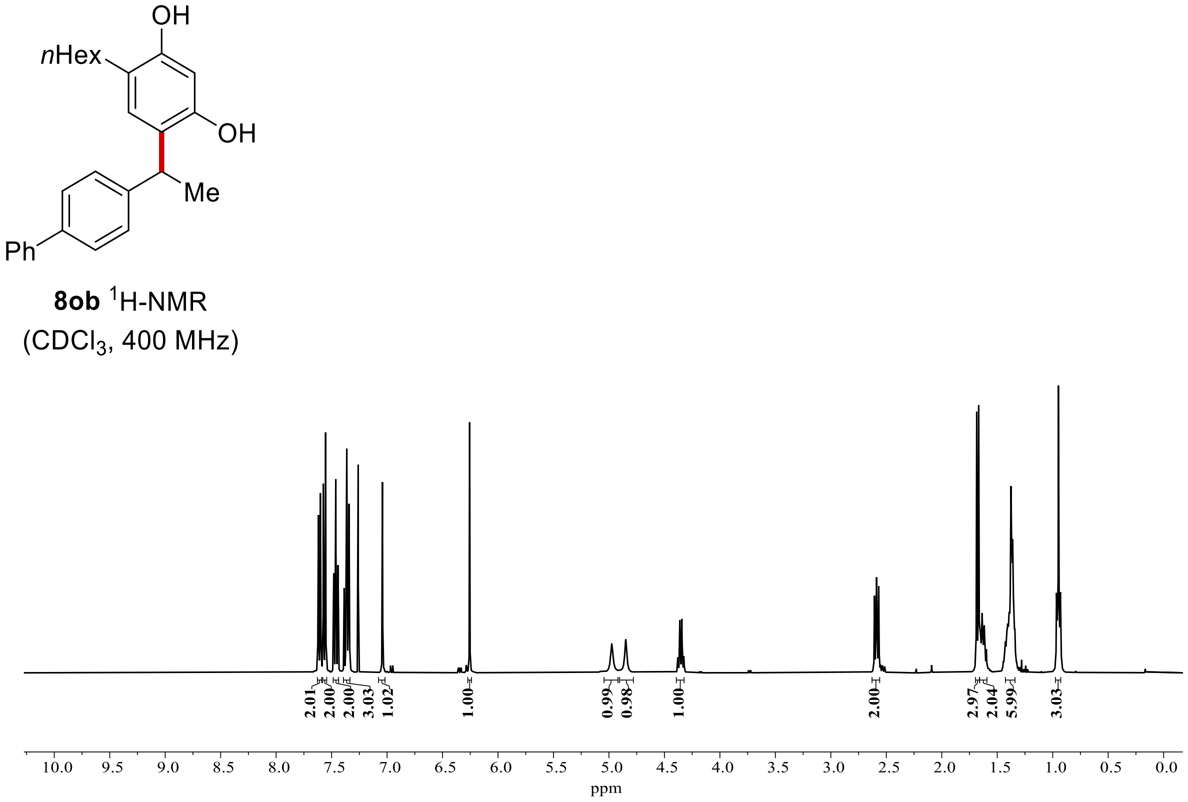
OH
nHex
OH
Me
Ph
8ob 1H-NMR
(CDCl3, 400 MHz)
2.01
2.00
2.00
3.03
1.02
1.00
0.99
0.98
1.00
2.00
2.97
2.04
5.99
3.03
ppm

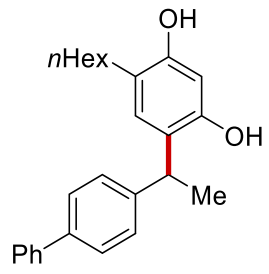

8ob ^{13}C-NMR
($CDCl_3$, 101 MHz)

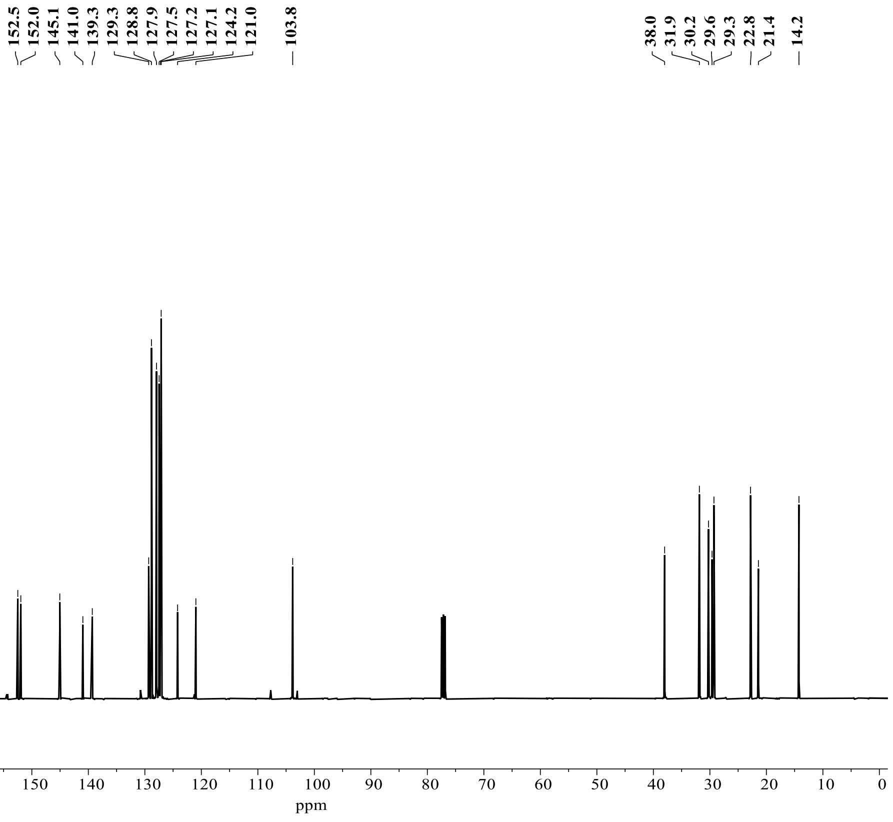

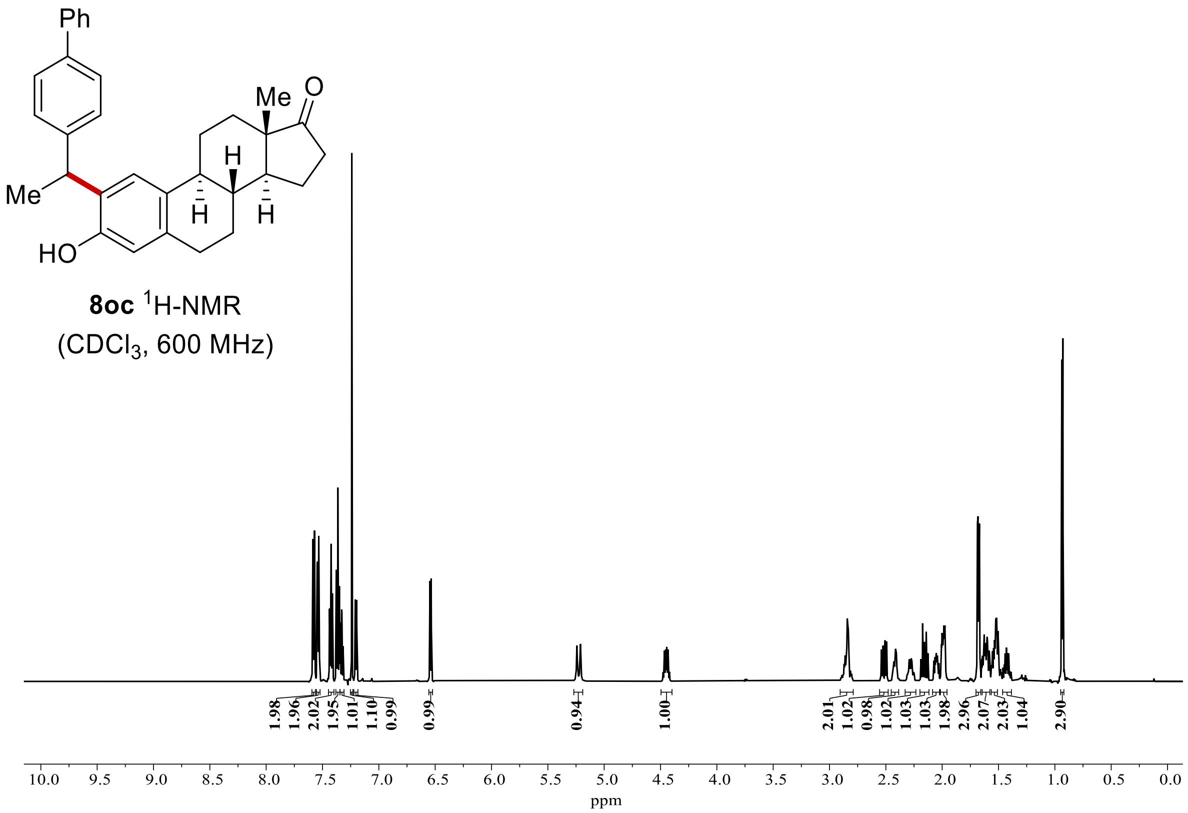
Ph
Me
O
H
Me
H
H
HO
8oc 1H-NMR
(CDCl3, 600 MHz)
1.98 1.96 2.02 1.95 1.01 1.10 0.99 0.99 0.94 1.00 2.01 1.02 0.98 1.02 1.03 1.03 1.98 2.96 2.07 2.03 1.04 2.90
10.0 9.5 9.0 8.5 8.0 7.5 7.0 6.5 6.0 5.5 5.0 4.5 4.0 3.5 3.0 2.5 2.0 1.5 1.0 0.5 0.0
ppm

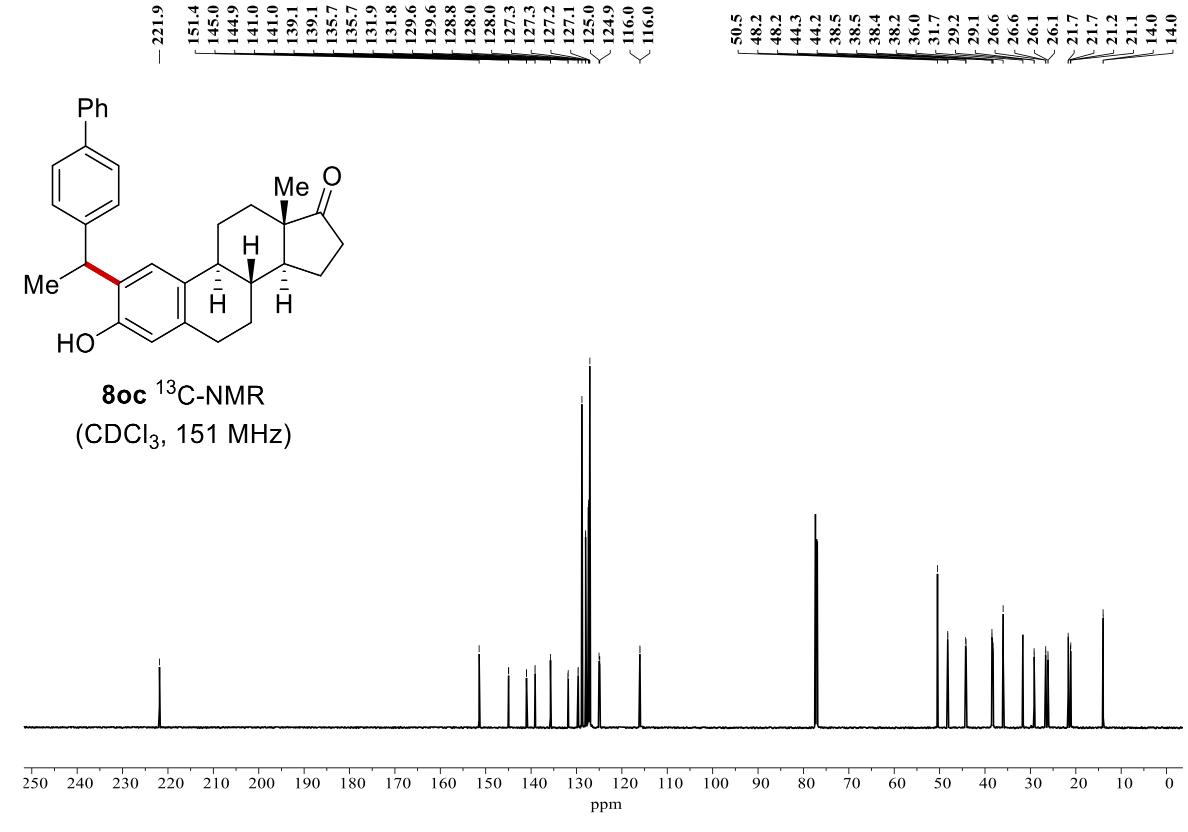
221.9
151.4 145.0 144.9 141.0 141.0 139.1 139.1 135.7 135.7 131.9 131.8 129.6 129.6 128.8 128.0 128.0 127.3 127.3 127.2 127.1 125.0 124.9 116.0 116.0
50.5 48.2 48.2 44.3 44.2 38.5 38.5 38.4 38.2 36.0 31.7 29.2 29.1 26.6 26.6 26.1 26.1 21.7 21.7 21.2 21.1 14.0 14.0
Ph
Me
O
H
Me
H
H
HO
8oc 13C-NMR
(CDCl3, 151 MHz)
250 240 230 220 210 200 190 180 170 160 150 140 130 120 110 100 90 80 70 60 50 40 30 20 10 0
ppm

15.References

[1] Y. Hu, L. Liang, W.-t. Wei, X. Sun, X.-j. Zhang, M. Yan, *Tetrahedron* **2015**, *71*, 1425-1430.

[2] W. Liu, X. Huang, J. T. Groves, *Nat. Protoc.* **2013**, *8*, 2348-2354.

[3] N. Shida, H. Takenaka, A. Gotou, T. Isogai, A. Yamauchi, Y. Kishikawa, Y. Nagata, I. Tomita, T. Fuchigami, S. Inagi, *J. Org. Chem.* **2021**, *86*, 16128–16133.

[4] J.-B. Xia, C. Zhu, C. Chen, *J. Am. Chem. Soc.* **2013**, *135*, 17494–17500.

[5] W. Liu, J. T. Groves, *Angew. Chem. Int. Ed.* **2013**, *52*, 6024–6027.

[6] W. Deng, W. Feng, Y. Li, H. Bao, *Org. Lett.* **2018**, *20*, 4245–4249.

[7] A. Vasilopoulos, D. L. Golden, J. A. Buss, S. S. Stahl, *Org. Lett.* **2020**, *22*, 5753–5757.

[8] I. N.-M. Leibler, M. A. Tekle-Smith, A. G. Doyle, *Nat. Commun.* **2021**, *12*, 6950.

[9] M. Haerter, P. Ellinghaus, K. Berhoerster, S. Greschat, K.-H. Thierauch (Bayer Schering Pharma AG), DE102008057344, **2010**.

[10] M. B. Nodwell, A. Bagai, S. D. Halperin, R. E. Martin, H. Knust, R. Britton, *Chem. Commun.* **2015**, *51*, 11783–11786.

[11] Q. Zhang, J. C. Mixdorf, G. J. Reynders, H. M. Nguyen, *Tetrahedron* **2015**, *71*, 5932-5938.

[12] J. Sheng, H.-Q. Ni, H.-R. Zhang, K.-F. Zhang, Y.-N. Wang, X.-S. Wang, *Angew. Chem. Int. Ed.* **2018**, *57*, 7634–7639.

[13] D. Cantillo, O. de Frutos, J. A. Rincon, C. Mateos, C. O. Kappe, *J. Org. Chem.* **2014**, *79*, 8486–8490.

[14] O. A. Wong, Y. Shi, *J. Org. Chem.* **2009**, *74*, 8377–8380.

[15] S. Zhao, Y. Guo, Z. Su, W. Cao, C. Wu, Q.-Y. Chen, *Org. Lett.* **2020**, *22*, 8634–8637.
